# Synthesis of withaferin a derivatives and exploratory evaluation of weight reduction and fasting blood glucose in DIO mice

**DOI:** 10.1039/d6ra01511e

**Published:** 2026-10-09

**Authors:** Zhiming Yan, Mingming Fan, Xinyi Liang, Changhong Wei, Yiheng Zhao, Hongyu Li, Tingzhuang Yi, Shixiong Yang

**Affiliations:** a Pharmaceutical College, Guangxi Medical University Nanning 530021 Guangxi PR China; b Department of Research & Clinical Laboratory, Department of Administrative Office, The Fifth Affiliated Hospital of Guangxi Medical University, The First People's Hospital of Nanning Nanning 530022 Guangxi PR China 258894453@qq.com 524651906@qq.com; c Department of Oncology, Affiliated Hospital of YouJiang Medical University For Nationalities, Guangxi Clinical Medical Research Center for Hepatobiliary Diseases, Guangxi Higher Education Engineering Research Center for Gastrointestinal Microecology and Health, Baise City Key Laboratory of Gastrointestinal Microecology and Health Baise 533000 Guangxi PR China yitingzhuang@ymun.edu.cn

## Abstract

Obesity and its cardiometabolic complications remain a major public-health burden. Leptin resistance is a common feature of hyperleptinemic obesity, but the molecular basis of leptin resensitization remains incompletely understood, making the rational optimization of leptin-sensitizing agents difficult. Withaferin A (1) has been reported to enhance leptin responsiveness and to reduce body weight and the fasting blood glucose in diet-induced obese (DIO) mice. In this study, a series of withaferin A derivatives (2–15) bearing modifications in the A/B-ring region and the C-27 side-chain/lactone moiety was synthesized and evaluated in DIO mice. The compounds were administered intraperitoneally at 1.2 mg kg^−1^ once daily for 21 days. Withaferin A produced sustained reductions in body weight, cumulative food intake, and fasting blood glucose. Most derivatives showed weaker or less consistent responses, although compounds 2, 6, 10, and 12 significantly reduced body weight relative to the vehicle group at day 21. Overall, withaferin A (1) showed the most consistent response across the measured endpoints. These results suggest that structural modifications in either the A/B-ring region or the C-27 side-chain/lactone moiety affect the *in vivo* phenotypic responses of withaferin A. This study provides preliminary phenotype-level *in vivo* SAR information for withaferin A derivatives and may assist the design of compounds for further evaluation.

## Abstract 2

Withaferin A and 14 derivatives were evaluated in diet-induced obese mice. Most derivatives led to smaller reductions in body weight and fasting blood glucose than the parent, providing preliminary *in vivo* structure–activity data.

## Graphical abstract

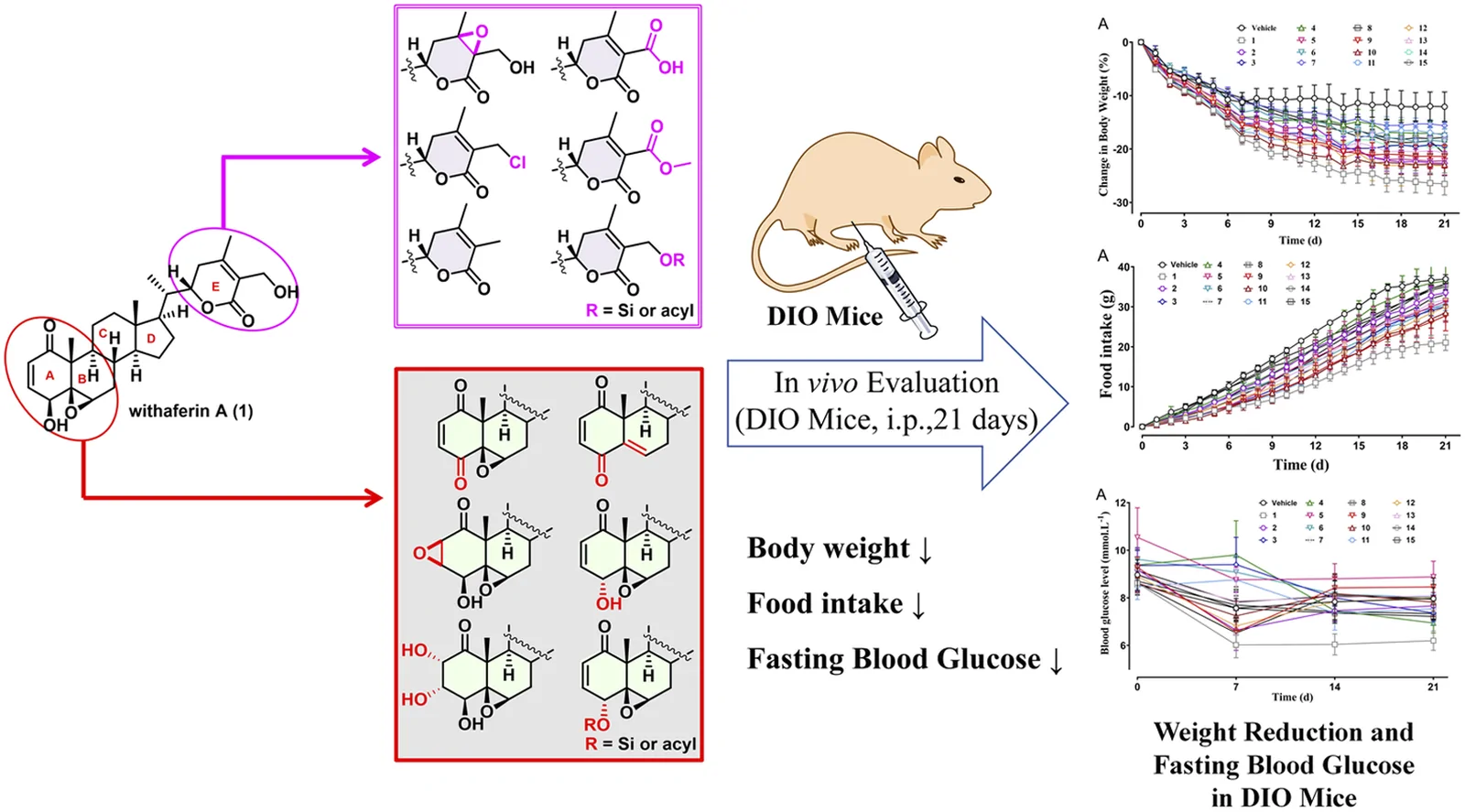

## 1. Introduction

Obesity remains a major public-health challenge, with prevalence continuing to rise in many low- and middle-income countries despite the increases slowing in some high-income countries.^1^ Its association with type 2 diabetes and cardiovascular disease,^2^ together with the growing global diabetes burden,^3^ highlights the need for treatments that reduce body weight and improve metabolic dysfunction. Semaglutide and tirzepatide have achieved substantial weight loss in clinical trials.^4,5^ Although oral semaglutide provides an alternative to injectable peptide therapy,^6^ its absorption and dosing constraints highlight the continued need for complementary therapeutic approaches, including small-molecule agents.^7^

Restoring leptin responsiveness offers a distinct approach. Leptin regulates appetite and energy expenditure,^8^ but common obesity is frequently accompanied by hyperleptinemia^9^ and leptin resistance.^10^ Leptin replacement is effective in rare leptin-deficient states but has limited efficacy in common obesity.^11^ Work linking hypothalamic endoplasmic reticulum (ER) stress to leptin resistance^12^ led to the identification of celastrol^13^ and withaferin A (1)^14^ as natural products with leptin-sensitizing and weight-reducing effects in hyperleptinemic diet-induced obese (DIO) mice. Withaferin A (1) was also reported to improve glucose metabolism through effects not fully explained by leptin sensitization.^14^

Mechanistic studies of celastrol have implicated IL1R1-dependent signaling,^15^ sympathetic nerve activity,^16^ and hypothalamic PTP1B/TCPTP inhibition.^17^ For withaferin A (1), incomplete knowledge of the relevant proximal targets and biomarkers limits target-guided optimization. Moreover, systematic comparisons of semisynthetic derivatives for *in vivo* metabolic endpoints in DIO mice remain scarce. It is therefore unclear which structural modifications preserve or diminish the responses observed with the parent compound. Phenotype-based evaluation provides an initial means of examining these relationships and obtaining preliminary metabolic structure–activity relationship (SAR) data.

Withaferin A (1) is a C28 steroidal lactone of the withanolide family found in *Withania somnifera*.^18^ The highly oxygenated, stereochemically complex structures of withanolides and the limited supply of individual congeners can hinder systematic SAR studies.^19,20^ Natural abundance and isolation yields vary with plant source and extraction conditions, making it difficult to obtain reproducible quantities for multigroup *in vivo* evaluation. Semisynthesis and late-stage diversification therefore provide practical routes to defined analogue sets for biological comparison.^21^ Withaferin A (1) derivatives have already been developed as apoptosis inducers,^22^ but the corresponding opportunities for metabolic SAR remain less explored.

Guided by these considerations, we pursued late-stage diversification of withaferin A (1) to prepare analogues 2–15, with modifications in the A/B-ring region and the C-27 side-chain/lactone region, for exploratory evaluation in DIO mice. The study compared body-weight, food-intake, and fasting-blood-glucose responses to examine the effects of these structural changes at the phenotypic level; it was not designed to test leptin sensitization or establish a molecular mechanism. The study was designed to generate preliminary phenotype-based *in vivo* SAR information rather than to establish exposure-normalized pharmacological potency.

## 2. Results and discussion

### 2.1. Design of withaferin a derivatives

Withaferin A (1) provides a densely functionalized withanolide scaffold that permits late-stage modification of both the A/B rings and the C-27 side chain and E-ring lactone. Guided by the reported reductions in body weight and fasting blood glucose produced by 1 in DIO mice,^14^ we assembled a focused panel of derivatives 2 to 15 to examine how defined structural changes at these regions affect the observed metabolic responses while preserving the core steroidal framework. The design also considered the electrophilic and stereochemically complex nature of withaferin A. The A-ring enone and 5,6-epoxide are prominent reactive features, whereas the unsaturated lactone and the C-4 and C-27 hydroxyl groups may influence molecular recognition, hydrogen-bonding capacity, and overall polarity.^17,23^ Previous studies have associated these structural features with the broad biological activities of withaferin A, although their roles in the metabolic effects observed in DIO mice have not been established.

Two principal regions were examined. First, modification of the A/B rings, represented mainly by compounds 2 to 5 and 14, altered the native enone and epoxide arrangement, the degree of oxygenation, or the local stereochemistry.^19,22^ This subset was designed to examine whether preservation of the native electrophilic geometry and stereochemical environment was important for the observed *in vivo* activity. Compounds 2 to 5 introduced different changes within the enone and epoxide region, whereas the C-4 epimer 14 served as a stereochemical comparator for evaluating the effect of a localized configurational change.

Second, modifications involving the C-27 side chain or E-ring lactone were introduced to examine the effects of hydrogen-bonding capacity, polarity, steric bulk, ionization state, and lipophilic character.^19,22^ Compounds 6 and 10 contain acylated C-4 and C-27 hydroxyl groups, thereby combining changes at the A-ring and side-chain positions. Compound 7 contains silyl ether groups at both C-4 and C-27, whereas compound 8 carries a bulky silyl substituent at C-27 while retaining the C-4 hydroxyl group. Replacement of the C-27 hydroxyl group with chloride afforded compound 9. Oxidation of the C-27 alcohol afforded the carboxylic acid 12, and subsequent methylation furnished the corresponding neutral ester 13. Compound 15, the naturally occurring C-27-deoxygenated analogue, served as an additional comparator for assessing the contribution of the native C-27 hydroxyl group. Compound 11 contains epoxide functionalities in both the A-ring region and the lactone ring, allowing the effect of simultaneous epoxidation at these two positions to be examined.

Because some analogues contain structural changes at more than one position, their effects were interpreted according to the overall modification pattern rather than being attributed to a single substituent. Overall, the derivative set was used to compare how changes in the A/B rings, the C-27 substituent, and the lactone region affected body weight, food intake, and fasting blood glucose in DIO mice.

### 2.2. Synthesis

As outlined in Scheme 1, withaferin A (1) served as the common starting material for the preparation of A/B-ring-modified withaferin A derivatives. Compounds 2 and 5, as well as the C-4 epimer 14 (4-*epi*-5,6-deoxywithaferin A), were prepared according to our previous report.^19^ Epoxidation of 1 under alkaline hydrogen peroxide conditions (NaOH/H_2_O_2_) afforded epoxide derivatives 3 and 11, which were isolated after chromatographic separation.^22^ In addition, Os-catalyzed *syn*-dihydroxylation of 1 using K_2_OsO_4_·2H_2_O/NMO in the presence of citric acid (*t*BuOH/H_2_O) delivered the corresponding vicinal diol 4.

**Scheme 1:**
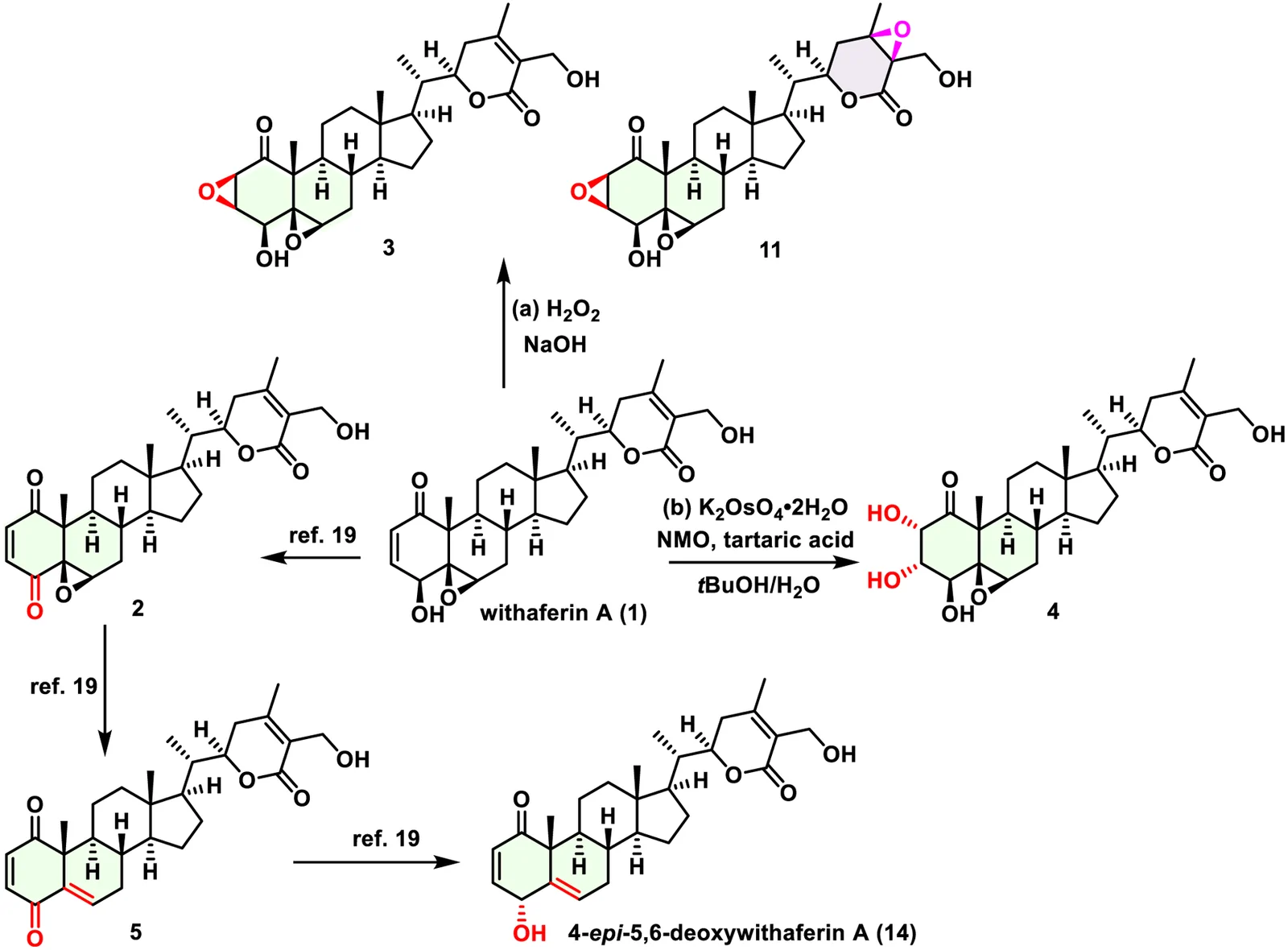
Synthetic route of withaferin A derivatives 2–5, 11 and 4-*epi*-5,6-deoxywithaferin A (14) from withaferin A (1), reagents and conditions: (a) H_2_O_2_, NaOH, CH_2_Cl_2_/MeOH; (b) K_2_OsO_4_·2H_2_O, NMO, citric acid, *t*BuOH/H_2_O. Compounds 2, 5, and 14 were prepared according to reported procedures.^19^

The synthetic sequences summarized in Scheme 2 enabled rapid access to a set of side-chain/lactone-modified analogues derived from withaferin A (1). Dual *O*-acylation of the C-4 and C-27 hydroxyl groups using (PhCO)_2_O or Ac_2_O in the presence of Et_3_N afforded the corresponding diesters 6 and 10, respectively. Global *O*-silylation of 1 with (CH_3_CH_2_)_3_SiCl/Et_3_N gave the bis-silyl ether 7, whereas installation of a bulkier silyl group at the side chain using (C_6_H_13_)_3_SiCl/Et_3_N provided the C-27 silyl ether 8. The side-chain chloride 9 was prepared according to a reported procedure.^22^ Oxidation of the C-27 alcohol of 1 with Jones reagent (acetone) furnished the carboxylic acid 12, which was subsequently converted to the corresponding methyl ester 13 by treatment with Me_2_SO_4_/K_2_CO_3_ in acetone. The naturally occurring 27-deoxywithaferin A (15) was obtained according to our previous report.^19^

**Scheme 2:**
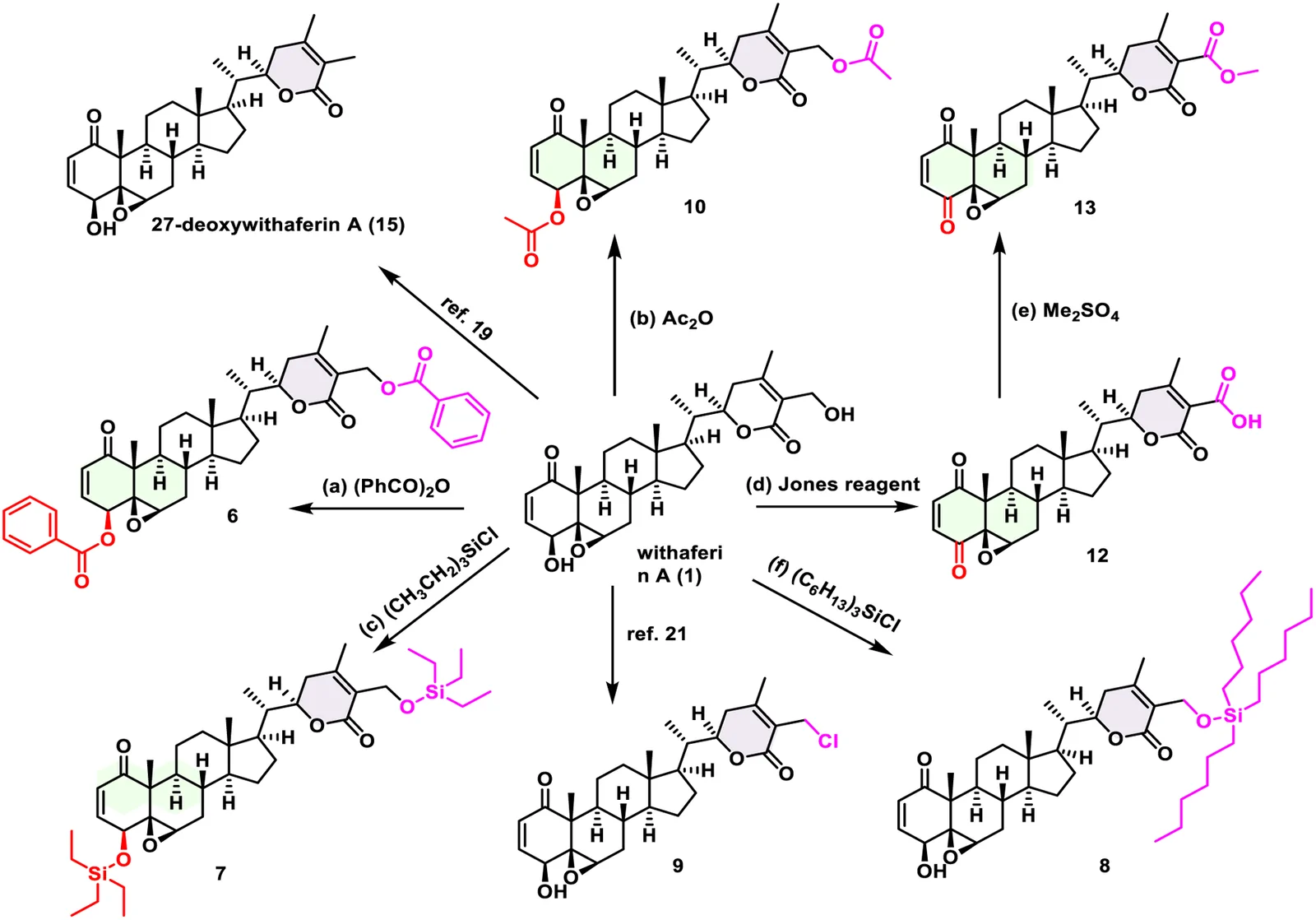
Synthetic route of withaferin A derivatives 6–10, 12–13, and 27-deoxywithaferin A (15) from withaferin A (1). Reagents and conditions: (a) (PhCO)_2_O, Et_3_N,CH_2_Cl_2_; (b) Ac_2_O, Et_3_N,CH_2_Cl_2_; (c) (CH_3_CH_2_)_3_SiCl, Et_3_N,CH_2_Cl_2_; (d) Jones reagent, acetone; (e) Me_2_SO_4_, K_2_CO_3_, acetone; (f) (C_6_H_13_)_3_SiCl, Et_3_N,CH_2_Cl_2_. Compounds 9 and 15 were prepared according to reported procedures.^19,22^

### 2.3. Animal experiment

#### 2.3.1 Body weight

To assess how structural modification of withaferin A affected body-weight responses *in vivo*, withaferin A (1) and derivatives 2–15 were evaluated in DIO mice under the same dosing regimen (see Experimental section). Consistent with previous reports of body-weight reduction by 1 in hyperleptinemic DIO mice,^14^ withaferin A (1) produced the largest and most sustained mean decrease in body weight during the 21 days treatment period (Fig. 2A). The vehicle group also showed a measurable decrease in body weight; therefore, the effects of the tested compounds were interpreted relative to the concurrent vehicle control. At day 21, withaferin A (1) showed the greatest mean body-weight reduction among the compounds tested under the present exploratory conditions (Fig. 2B).

**Fig. 1:**
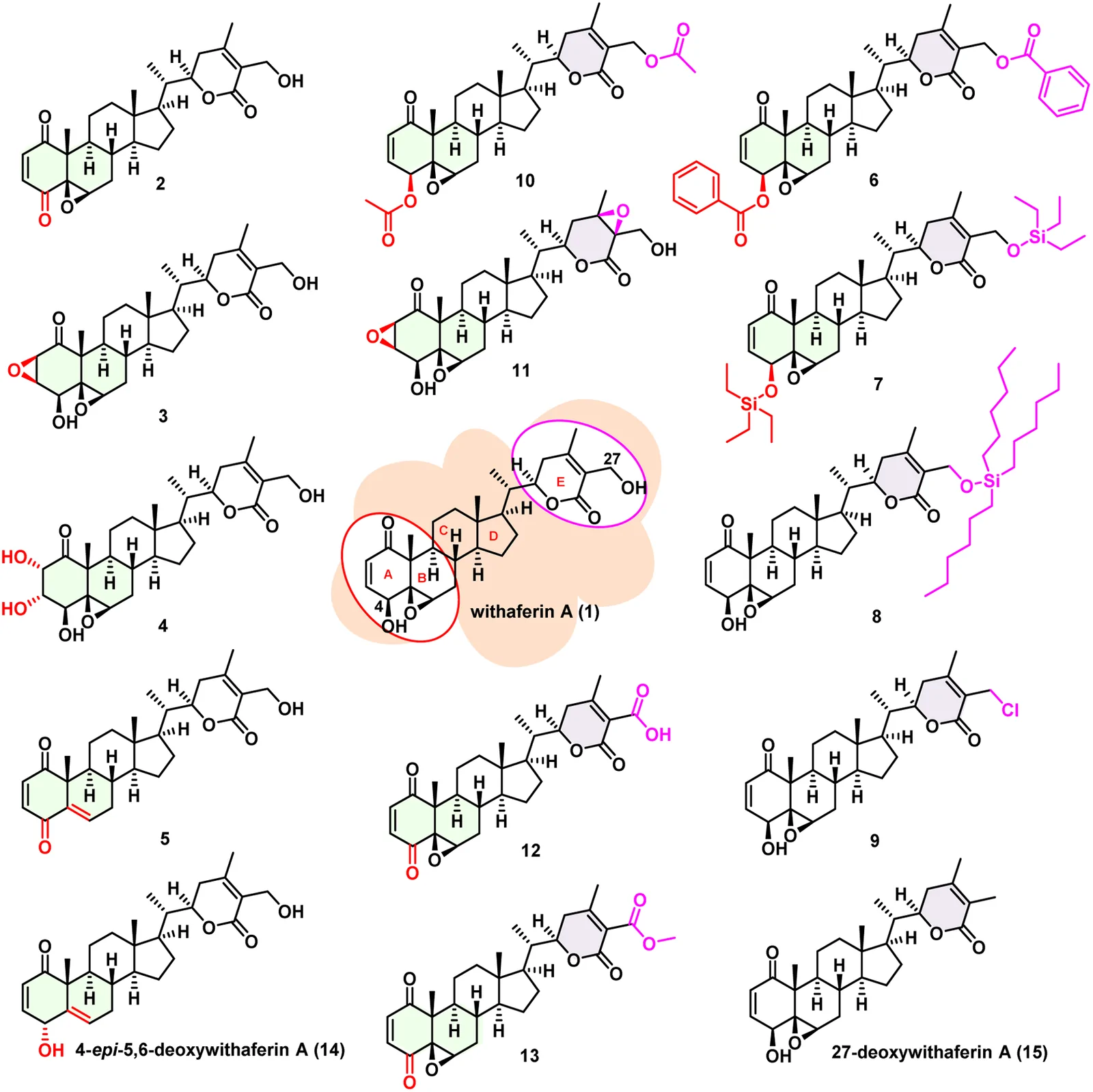
Overview of withaferin A derivatives: modification sites and derivative classes.

**Fig. 2:**
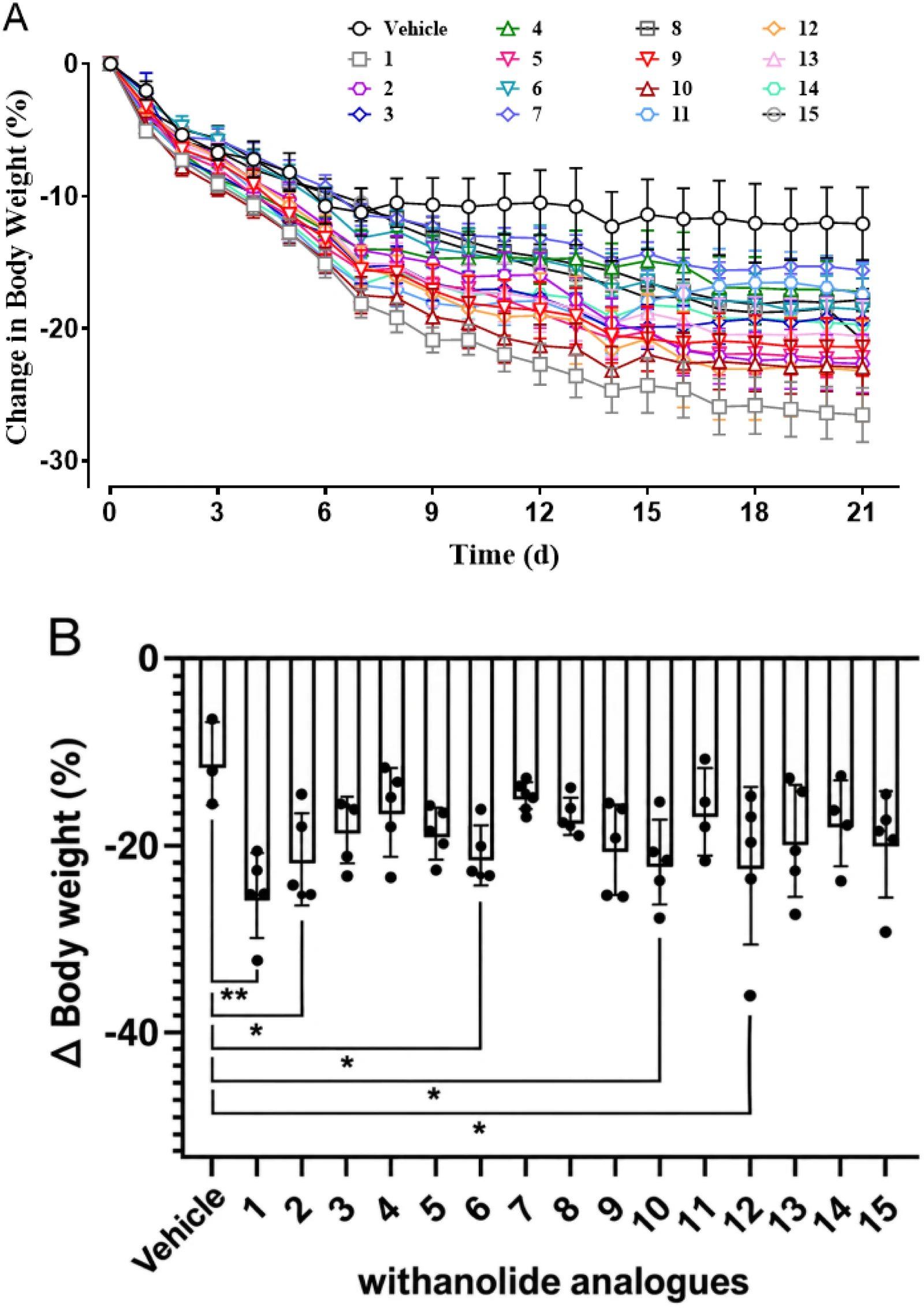
Effects of withaferin A derivatives on body weight in DIO mice. (A) Time course of body-weight change during the treatment period in DIO mice treated with vehicle (DMSO), withaferin A (1), or derivatives 2–15. The injection volume was adjusted according to body weight, as described in Section 4.3. Body weight is expressed as the percentage change from baseline on day 0. Data are presented as mean ± SEM (vehicle, *n* = 3; each compound-treated group, *n* = 5). (B) Body-weight change at day 21. Data are presented as mean ± SD, with individual values shown (vehicle, *n* = 3; each compound-treated group, *n* = 5). Statistical significance *versus* vehicle was determined using one-way ANOVA followed by Dunnett's multiple-comparisons test (*p < 0.05, **p < 0.01, ***p < 0.001; ns, not significant). Owing to the small group sizes, the endpoint comparisons are considered exploratory. Compound structures are shown in Fig. 1.

Across the derivatives, body-weight responses were generally smaller and more variable than that observed with 1. In addition to the parent compound, only derivatives 2, 6, 10, and 12 showed statistically significant reductions relative to vehicle at day 21 (Fig. 2B). Among the compounds primarily modified in the A/B-ring region, 2 was the only derivative to retain a significant effect, whereas compounds 3–5 and 14 did not. Compound 11, which contains epoxide functionalities in both the A-ring region and the lactone ring, also showed no significant reduction relative to vehicle. These findings suggest that the body-weight response is sensitive to changes in the native electrophilic and stereochemical environment of the withaferin A scaffold.

The modifications involving the C-27 side chain showed partial tolerance. Acylation of the C-4 and C-27 hydroxyl groups in compounds 6 and 10, as well as oxidation of the C-27 and C-4 alcohol to the carboxylic acid in compound 12, retained statistically significant effects relative to vehicle, although their mean reductions were smaller than that observed with 1. In contrast, the bis-silylated analogue 7, the C-27-silylated analogue 8, chloride 9, methyl ester 13, and C-27-deoxygenated analogue 15 were not statistically different from vehicle at day 21. The different responses of carboxylic acid 12 and methyl ester 13 suggest that the ionization state and hydrogen-bonding properties of the C-27 substituent may influence the *in vivo* response. The lack of significant effects for compounds 7 and 8 may also be associated with their increased steric bulk and lipophilic character. However, the present data cannot distinguish changes in intrinsic pharmacological activity from differences in systemic exposure.

Overall, both the A/B-ring architecture and the C-27 substituent influenced body-weight responses in DIO mice, although the small cohorts and multiple structural changes in some analogues limit these observations to preliminary *in vivo* SAR trends. Because safety parameters were not assessed and the vehicle group also showed a decrease in body weight, nonspecific or treatment-related effects cannot be excluded. These findings should therefore be interpreted as exploratory responses relative to the concurrent vehicle group rather than evidence of therapeutic weight loss.

#### 2.3.2 Food intake

Previous studies reported that the body-weight reduction produced by withaferin A (1) in hyperleptinemic DIO mice was accompanied by reduced food intake.^14^ We therefore monitored cumulative food intake as a secondary exploratory endpoint. Food consumption was measured at the cage level and expressed as the average intake per mouse, with each cage treated as an independent experimental unit.

Withaferin A (1) produced the clearest and most sustained reduction in cumulative food intake relative to vehicle during the 21-days treatment period (Fig. 3A). At day 21, only compound 1 showed a statistically significant reduction relative to vehicle, whereas none of the derivatives reached statistical significance (Fig. 3B). The reduction in food intake produced by compound 1 was consistent with its pronounced body-weight effect, suggesting that decreased food consumption contributed to the response of the parent compound. In contrast, compounds 2, 6, 10, and 12 significantly reduced body weight but did not significantly reduce cumulative food intake, indicating the available data do not demonstrate that reduced food consumption alone accounted for their body-weight responses. Most A/B-ring-modified derivatives and the bulky silylated analogues 7 and 8 also did not show a statistically significant food-intake response under the present exploratory conditions. These structural changes may reduce intrinsic activity or alter physicochemical properties and systemic exposure, although these possibilities cannot be distinguished without mechanistic and pharmacokinetic data. Given the small number of independent cages, the food-intake results should be regarded as supportive and exploratory and do not establish appetite suppression as the mechanism of body-weight reduction.

**Fig. 3:**
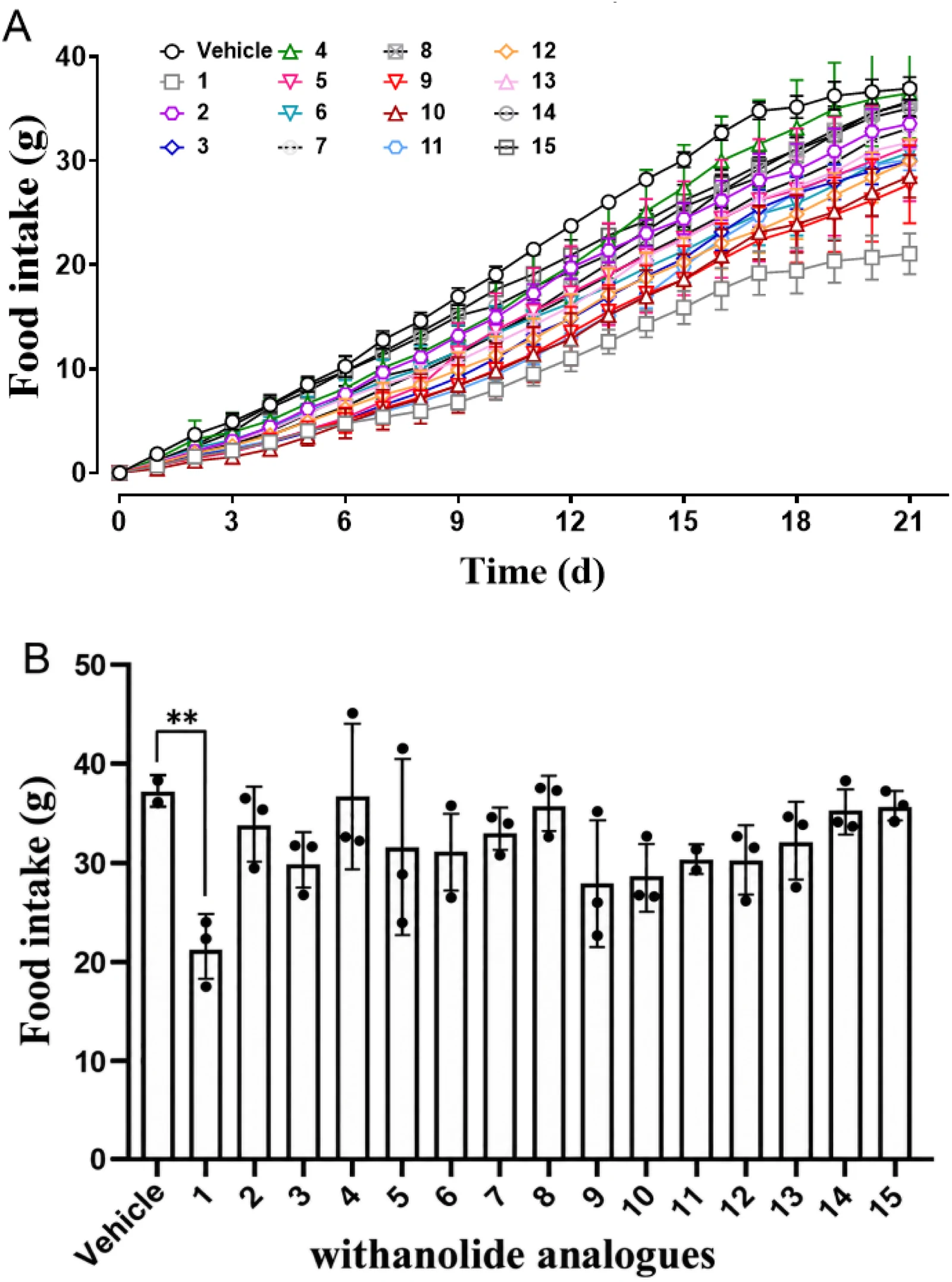
Effects of withaferin A derivatives on food intake in DIO mice. (A) Cumulative food intake during the 21-days treatment period in DIO mice treated with vehicle, withaferin A (1), or derivatives 2–15. Food consumption was recorded for each cage and divided by the number of mice housed in that cage to obtain the mean intake per mouse for each cage. Data are presented as mean ± SEM of the cage-normalized values. The vehicle group comprised two cages with two mice per cage (*n* = 2 cages), and each compound-treated group comprised five mice housed across three cages with cage occupancies of 2, 2, and 1 mouse (*n* = 3 cages). (B) Cumulative food intake per mouse at day 21, calculated from cage-level measurements. Data are presented as mean ± SD, with individual cage values shown. Statistical significance *versus* vehicle was determined using one-way ANOVA followed by Dunnett's multiple-comparisons test (*p < 0.05, **p < 0.01, ***p < 0.001; ns, not significant). Each cage was treated as an independent experimental unit. Owing to the small number of independent cages, these comparisons are considered exploratory. Compound structures are shown in Fig. 1.

#### 2.3.3 Fasting blood glucose

Fasting blood glucose was measured on days 0, 7, 14, and 21 and summarized at the day-21 endpoint (Fig. 4). Among the tested groups, withaferin A (1) showed the most consistent downward trajectory in fasting blood glucose, with a decrease evident by day 7 that was generally maintained through day 21. Most derivatives showed smaller or more variable changes relative to vehicle, and several trajectories approached the vehicle group by the endpoint (Fig. 4A and B). Because statistical testing was limited to day-21 comparisons with vehicle, differences between compound 1 and the individual derivatives are interpreted descriptively.

**Fig. 4:**
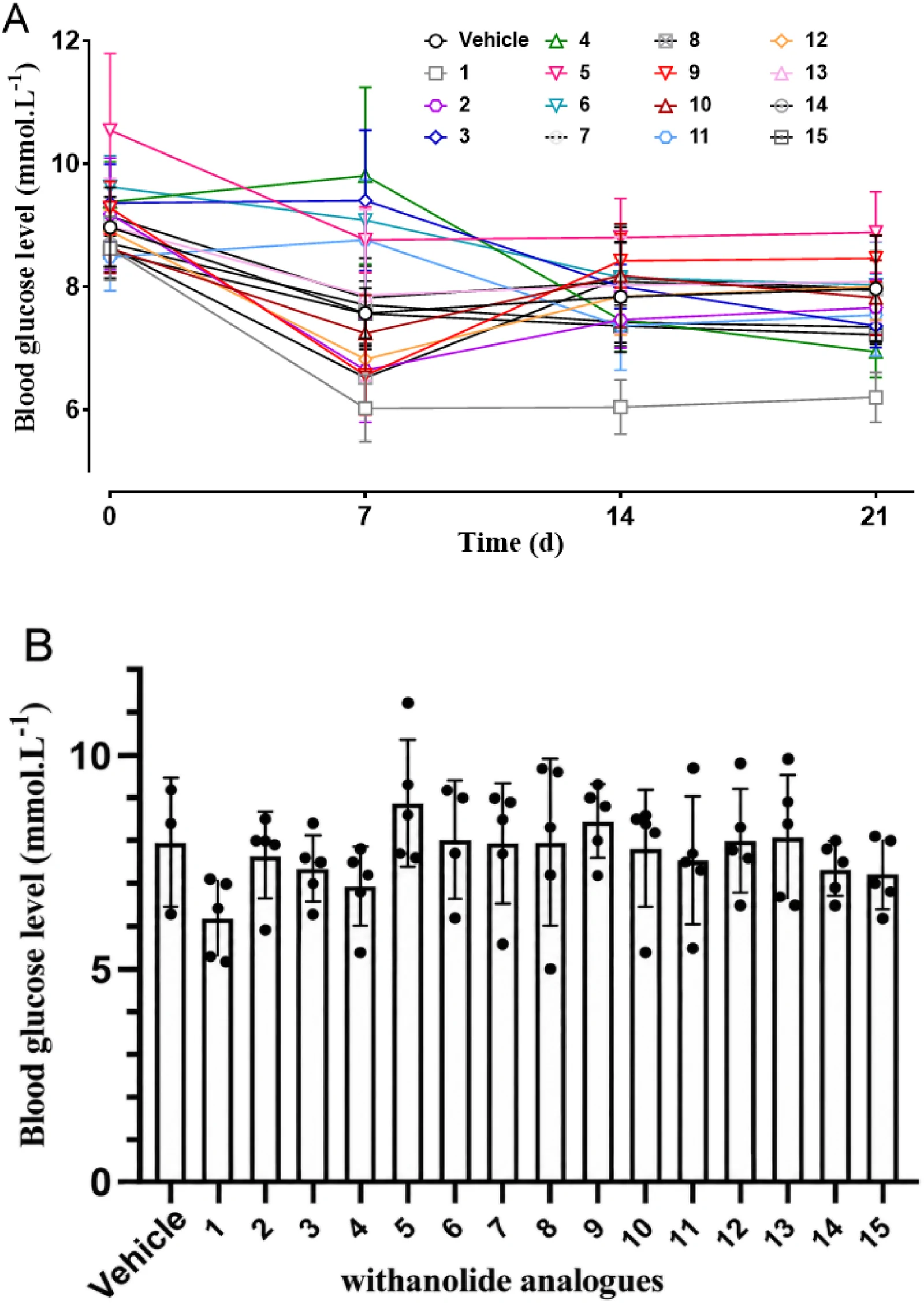
Effects of withaferin A derivatives on fasting blood glucose in DIO mice. (A) Fasting blood glucose concentrations on days 0, 7, 14, and 21 in DIO mice treated with vehicle, withaferin A (1), or derivatives 2–15. Data are presented as mean ± SEM (vehicle, *n* = 3 mice; each compound-treated group, *n* = 5 mice). (B) Fasting blood glucose concentrations at the day-21 endpoint. Data are presented as mean ± SD, with individual mouse values shown (vehicle, *n* = 3 mice; each compound-treated group, *n* = 5 mice). Statistical significance for the day-21 endpoint was determined by comparing each treatment group with vehicle using one-way ANOVA followed by Dunnett's multiple-comparisons test (*p < 0.05, **p < 0.01, ***p < 0.001; ns, not significant). Given the small group sizes, the endpoint comparisons are considered exploratory. Compound numbering follows Fig. 1.

The fasting-glucose profiles suggest that modifications in both the steroidal core and the C-27 region can influence this response. Compounds 3, 4, and 14, which alter the oxidation pattern or stereochemistry of the A/B-ring region, did not reproduce the sustained profile observed with compound 1. Compound 11, which contains epoxide functionalities in both the A-ring and lactone regions, likewise showed a less persistent response. Dual C-4/C-27 acylation in compounds 6 and 10, C-4/C-27 bis-silylation in compound 7, C-27 silylation in compound 8, and replacement of the C-27 hydroxyl with chloride in compound 9 were also associated with more variable or attenuated trajectories. The differing profiles of the C-27 carboxylic acid 12 and its methyl ester 13 are consistent with previous semisynthetic studies showing that modification of the side-chain/lactone region of withaferin A can alter biological activity.^22^ This contrast suggests that ionization and polarity at C-27 may contribute to the observed response. However, no direct pairwise statistical comparison or pharmacokinetic analysis was performed, and the present data cannot distinguish altered intrinsic activity from differences in exposure, clearance, or distribution. Although serial fasting-blood-glucose measurements provided a temporal view of the glucose response during treatment, this endpoint alone does not establish comprehensive glycemic improvement. Glucose-tolerance and insulin-tolerance tests, plasma insulin measurements, and tissue-signaling analyses were not performed. Therefore, the present findings are limited to changes in fasting blood glucose and should not be interpreted as evidence of improved insulin sensitivity, glucose tolerance, or overall glycemic control.

Previous studies have linked the metabolic effects of withaferin A and related leptin-sensitizing natural products to relief of hypothalamic endoplasmic-reticulum stress and modulation of leptin-associated signaling pathways, including IL1R1- and PTP1B/TCPTP-related mechanisms.^12–16^ These pathways could indirectly influence fasting blood glucose through changes in food intake, body weight, insulin sensitivity, hepatic glucose production, or peripheral glucose utilization. However, none of these mechanisms was examined in the present study. Because plasma insulin, leptin, adiponectin, glucose tolerance, insulin tolerance, and tissue-signaling markers such as pAKT and pSTAT3 were not measured, the current findings establish only a phenotype-level fasting-blood-glucose response and do not identify a specific molecular mechanism.

#### 2.3.4 Summary of exploratory SAR

The structural modifications and their associated phenotypic responses are summarized in Fig. 5. C-4 oxidation (2), C-4/C-27 diacylation (6 and 10), and combined C-4 oxidation/C-27 carboxylic acid formation (12) retained significant body-weight reductions relative to vehicle at day 21. All four derivatives retained the native A-ring enone and 5,6-epoxide. In contrast, derivatives involving modification of either of these structural regions (3–5, 11, and 14) did not show a significant body-weight reduction relative to vehicle at day 21. On the common C-4-oxo scaffold, acid 12 and methyl ester 13 showed numerically different mean body-weight responses, making the C-27 alcohol/acid/ester series (2, 12 and 13) useful for further comparison. Mean fasting blood glucose at day 21 was numerically higher for all derivatives than for 1. These observations suggest that C-4/C-27 substitution may warrant further exploration, particularly while retaining the native A-ring enone and 5,6-epoxide. Direct comparisons between compounds, together with matched exposure measurements, are needed to determine whether these preliminary patterns reflect differences in intrinsic pharmacological activity.

**Fig. 5:**
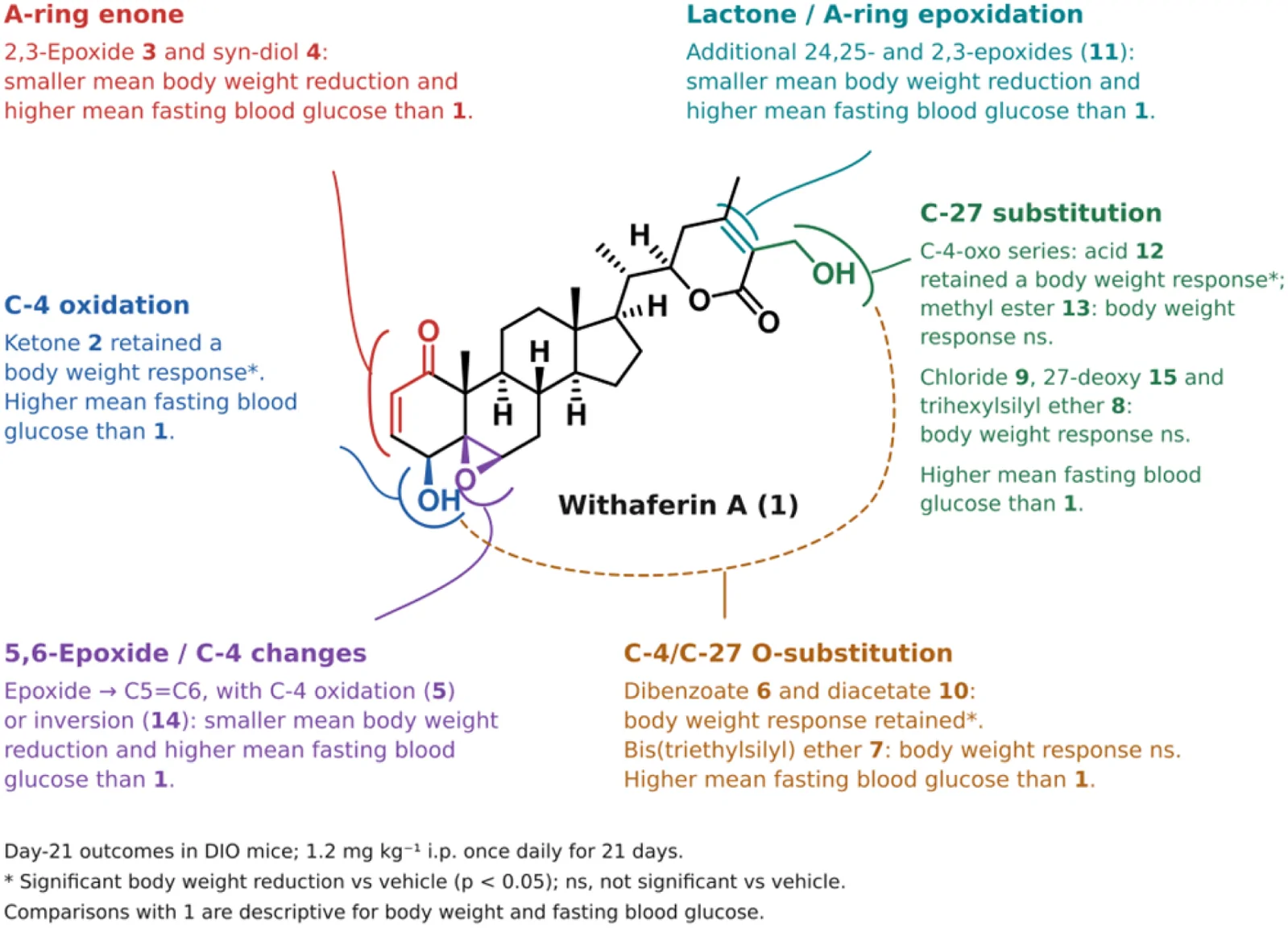
Exploratory *in vivo* SAR summary of withaferin A (1) and its derivatives (2–15) in DIO mice.

## 3. Conclusions

In summary, late-stage semisynthetic modification of withaferin A (1) afforded a panel of derivatives (2–15) containing changes in the A/B-ring region and the C-27 side-chain/lactone region. In this exploratory DIO mouse study, withaferin A (1) showed the largest and most consistent mean reductions in body weight, food intake, and fasting blood glucose. Most derivatives produced weaker or more variable responses. Compounds 2, 6, 10, and 12 retained significant body-weight reductions relative to vehicle at day 21, but their food-intake and fasting-glucose profiles were less consistent. These results suggest that retention of the *in vivo* phenotype may depend on several native functional groups in the parent scaffold.

Because the statistical analyses compared each treatment group with vehicle rather than directly comparing the derivatives with withaferin A (1), the results should be interpreted as preliminary phenotype-level SAR. In addition, fasting blood glucose alone does not establish comprehensive glycemic improvement. The small cohort sizes, the limited number of independent cages used for food-intake analysis, and the absence of standard comparators, mechanistic biomarkers, safety measurements, and PK/ADME data further limit interpretation. Larger, well-powered studies incorporating appropriate positive controls, metabolic and signaling markers, safety assessment, and compound-specific exposure measurements will be required to validate these findings and guide further optimization.

## 4. Experimental

### 4.1. General experimental procedures

Reagents and solvents were purchased from commercial suppliers and used as received (typical stated purity >95%) unless otherwise noted. Reactions were carried out with magnetic stirring. Moisture- or air-sensitive transformations were conducted under an atmosphere of nitrogen using oven-dried glassware and anhydrous solvents. Temperatures refer to the external bath. Reactions at 0 °C were performed in a Dewar bath containing an ice water mixture. Reaction progress was monitored by TLC on silica gel plates (Tsingdao, 60F-254, 0.25 mm, glass-backed), with visualization under UV light (254 nm). Flash column chromatography was performed on silica gel (Tsingdao, 60; particle size 0.040–0.063 mm). Reported yields correspond to isolated products purified by column chromatography unless otherwise stated. NMR spectra were recorded on a Bruker Avance 600 spectrometer (^1^H, 600 MHz; ^13^C, 150 MHz). Chemical shifts are reported in ppm and referenced to residual solvent signals of CDCl_3_ (*δ*_H_ = 7.26 ppm, *δ*_C_ = 77.16 ppm) unless otherwise indicated. HRMS (High-Resolution Mass Spectrometry) data were recorded on a Thermo Ultimate 3000 Q-Exactive Focus mass spectrometer.

### 4.2. Chemistry

#### 4.2.1 Preparation of compounds 3 and 11

To a solution of withaferin A (50.0 mg, 0.106 mmol, 1.0 equiv.) in CH_2_Cl_2_ (2.5 mL)/MeOH (5 mL) was added 30% H_2_O_2_ (w/w, 0.3 mL), followed by the addition of NaOH (4 N, 0.1 mL). The reaction mixture was stirred at room temperature for 2 min. H_2_O (5 mL) was added and extracted with CH_2_Cl_2_ (3 × 5 mL), The combined organic extracts were dried over anhydrous Na_2_SO_4_, filtered, and concentrated. The resulting residue was purified by silica gel flash chromatography (eluent: EtOAc/PE, 1/6 to 1/1) to afford compound 3 (22 mg, yield: 43%) and 11 (12 mg, yield: 23%) as a light yellow solid.

#### 4.2.2 Preparation of compound 4

To a stirred solution of withaferin A (50.0 mg, 0.106 mmol, 1.0 equiv.) in *t*BuOH (0.8 mL) and H_2_O (0.4 mL) were added 4-methylmorpholine *N*-oxide (NMO, 18.0 mg, 0.159 mmol, 1.5 equiv.) and citric acid (40.7 mg, 0.212 mmol, 2.0 equiv). After being stirred for 5 min at room temperature, K_2_OsO_4_·2H_2_O (0.8 mg, 0.002 mmol, 0.02 equiv.) was added, and the reaction mixture was stirred at room temperature for 2 h. Upon completion, the reaction was quenched by addition of saturated aqueous Na_2_S_2_O_3_ (0.5 mL) and stirred for an additional 30 min. The mixture was then diluted with EtOAc (3 mL) and partitioned. The aqueous layer was extracted with EtOAc (3 × 3 mL). The combined organic extracts were dried over anhydrous Na_2_SO_4_, filtered, and concentrated under reduced pressure. The crude residue was purified by flash column chromatography on silica gel (EtOAc) to afford compound 4 (35.8 mg, yield: 67%) as a white solid.

#### 4.2.3 Preparation of compound 6

To a stirred solution of withaferin A (50.0 mg, 0.106 mmol, 1.0 equiv.) in anhydrous CH_2_Cl_2_ (2.5 mL) was added triethylamine (0.29 mL, 2.12 mmol, 20 equiv.), followed by benzoic anhydride (52.8 mg, 0.233 mmol, 2.2 equiv.) at room temperature. The reaction mixture was stirred at room temperature for 4 h. The solvent was removed directly under reduced pressure, and the crude residue was purified by flash column chromatography on silica gel (eluent: EtOAc/PE, 1 : 15 to 1 : 8) to afford compound 6 (45.3 mg, yield: 63%) as a light yellow solid.

#### 4.2.4 Preparation of compound 7

To a stirred solution of withaferin A (50.0 mg, 0.106 mmol, 1.0 equiv.) in anhydrous CH_2_Cl_2_ (2.5 mL) was added triethylamine (0.30 mL, 2.12 mmol, 20 equiv.), followed by triethylchlorosilane (35.1 mg, 0.233 mmol, 2.2 equiv.) at room temperature. The reaction mixture was stirred at room temperature for 3 h. The solvent was removed directly under reduced pressure, and the crude residue was purified by flash column chromatography on silica gel (eluent: EtOAc/PE, 1 : 15 to 1 : 10) to afford compound 7 (52.5 mg, yield: 71%) as a light yellow solid.

#### 4.2.5 Preparation of compound 8

To a stirred solution of withaferin A (50.0 mg, 0.106 mmol, 1.0 equiv.) in anhydrous CH_2_Cl_2_ (2.5 mL) at room temperature was added triethylamine (0.15 mL, 1.06 mmol, 10 equiv.). Chlorotrihexylsilane (43.9 mg, 0.138 mmol, 1.3 equiv.) was then added dropwise, and the reaction mixture was stirred at room temperature until completion (TLC monitoring). The solvent was removed directly under reduced pressure, and the crude residue was purified by flash column chromatography on silica gel (eluent: EtOAc/petroleum ether, 1 : 30 to 1 : 10) to afford compound 8 (43.7 mg, yield: 54%) as a pale yellow oil.

#### 4.2.6 Preparation of compound 10

To a stirred solution of withaferin A (50.0 mg, 0.106 mmol, 1.0 equiv.) in anhydrous CH_2_Cl_2_ (2.5 mL) was added triethylamine (0.30 mL, 2.12 mmol, 20 equiv.), followed by acetic anhydride (54.0 mg, 0.530 mmol, 5.0 equiv.) at room temperature. The reaction mixture was stirred at room temperature for 2 h. The solvent was removed directly under reduced pressure, and the crude residue was purified by flash column chromatography on silica gel (eluent: EtOAc/PE, 1 : 15 to 1 : 6) to afford compound 10 (48.0 mg, yield: 81%) as a white solid.

#### 4.2.7 Preparation of compound 12

To a stirred solution of withaferin A (50.0 mg, 0.106 mmol, 1.0 equiv.) in acetone (3.0 mL) at 0 °C was added Jones reagent (CrO_3_ in H_2_SO_4_, 2.0 M, 0.32 mL, 0.636 mmol, 6.0 equiv.) dropwise. The reaction mixture was allowed to warm to room temperature and stirred for 72 h. The reaction was quenched by careful addition of *i*-PrOH until the orange color disappeared, and the mixture was filtered through a short pad of Celite. The filtrate was concentrated under reduced pressure, and the residue was purified by flash column chromatography on silica gel (eluent: MeOH/CH_2_Cl_2_, 1 : 50 to 1 : 25) to afford compound 12 (16.9 mg, yield: 33%) as a light yellow solid.

#### 4.2.8 Preparation of compound 13

To a stirred solution of compound 12 (23.0 mg, 0.048 mmol, 1.0 equiv.) in acetone (1.5 mL) at room temperature was added K_2_CO_3_ (19.9 mg, 0.144 mmol, 3.0 equiv.). Dimethyl sulfate (9.1 mg, 0.072 mmol, 1.5 equiv.) was then added dropwise, and the reaction mixture was stirred at room temperature until completion (TLC monitoring). The reaction mixture was filtered to remove inorganic salts and concentrated under reduced pressure. The residue was diluted with EtOAc, and the aqueous layer was extracted with EtOAc (3 × 5 mL). The combined organic extracts were dried over anhydrous Na_2_SO_4_, filtered, and concentrated under reduced pressure. The crude product was purified by flash column chromatography on silica gel (eluent: EtOAc/petroleum ether, 1 : 3 to 2 : 1) to afford compound 13 (11.2 mg, yield: 47%) as a light yellow solid.

### 4.3. Biological activities

All animal procedures were approved by the Ethics Professional Committee of the Academic Committee at Jiangsu Normal University (approval no. JSNU-IACUC-2025057) and were performed in accordance with the Guide for the Care and Use of Laboratory Animals.

Male C57BL/6J mice weighing 18–22 g at the initiation of high-fat-diet feeding were maintained under controlled conditions with a 12 h light/dark cycle at 22–25 °C and had free access to water and diet unless otherwise stated. Diet-induced obesity was established by feeding the mice a high-fat diet providing 60% of energy from fat (Research Diets, D12492) for 18–20 weeks. Mice weighing 40–50 g were subsequently enrolled in the efficacy study and allocated to treatment groups after stratification according to baseline body weight and, where applicable, fasting blood glucose.

Withaferin A (1) and derivatives 2–15 were prepared in DMSO at 2.5 mg mL^−1^ and administered intraperitoneally at 1.2 mg kg^−1^ once daily for 21 consecutive days. No additional cosolvent or aqueous diluent was used, and the final DMSO concentration was therefore 100% (v/v). The injection volume was calculated according to the body weight of each mouse and corresponded to 0.48 mL kg^−1^. Mice in the vehicle group received DMSO at the same volume as the treatment groups.

For food-intake measurements, food consumption was recorded at the cage level. The vehicle group comprised four mice housed in two cages, with two mice per cage. Each compound-treated group comprised five mice housed in three cages, with cage occupancies of 2, 2, and 1 mouse. Pre-weighed food was supplied to each cage. At each scheduled measurement, the amount consumed per cage was calculated by subtracting the remaining food and collected visible spillage from the amount supplied. This value was divided by the number of mice housed in the cage to obtain the mean food intake per mouse for that cage. Cumulative food intake was calculated over the 21-days treatment period. Each cage was treated as an independent experimental unit; thus, the statistical sample sizes were *n* = 2 cages for the vehicle group and *n* = 3 cages for each compound-treated group.

Fasting blood glucose was measured on days 0, 7, 14, and 21. Mice were fasted for 14–16 h before each measurement, with free access to water. The mice were gently restrained, the tail tip was nicked using sterile scissors, and the first drop of blood was discarded. Glucose was measured from the second drop using a GA-3 handheld glucometer (Sinocare, China). Each mouse was treated as an independent experimental unit for the body-weight and fasting-glucose analyses.

Data are presented as mean ± SEM for time-course plots and mean ± SD for day-21 endpoint comparisons. For Fig. 2 and 4, the reported *n* values represent individual mice (vehicle, *n* = 3 mice; each compound-treated group, *n* = 5 mice). For Fig. 3, the reported *n* values represent independent cages (vehicle, *n* = 2 cages, four mice in total; each compound-treated group, *n* = 3 cages, five mice in total). Day-21 endpoint comparisons were performed using one-way ANOVA followed by Dunnett's multiple-comparisons test, with each treatment group compared with vehicle. No formal statistical comparisons were performed between withaferin A (1) and the individual derivatives. Statistical analyses were conducted using GraphPad Prism 8, and *p* < 0.05 was considered statistically significant. Given the small sample sizes, particularly the limited number of independent cages in the food-intake analysis, the statistical findings should be interpreted as exploratory and require confirmation in larger, appropriately powered studies.

## Author contributions

Z. Y., M. F. and X. L. performed synthetic chemistry and compound characterization. Z. Y., Y. Z. and H. L. performed experiments on biological activity. Z. Y., C. W., T. Y., and S. Y. oversaw experimental design, and Z. Y. conceived the study and wrote the manuscript with final approval from all authors. Z. Y., M. F., and X. L. contributed equally.

## Conflicts of interest

The authors declare no conflicts of interest.

## Supplementary Material

RA-OLF-D6RA01511E-s001

## Data Availability

Data is provided within the supplementary information (SI) files. Supplementary information: chemical characterization of all compounds, NMR (^1^H NMR, ^13^C NMR) and HRMS spectra for related compounds, NMR raw data files for related compounds. See DOI: https://doi.org/10.1039/d6ra01511e.
