# Supplementary material for: Synthesis of withaferin a derivatives and exploratory evaluation of weight reduction and fasting blood glucose in DIO mice
Source: RSC Adv. 2026 Oct 9;16(47):61832–40. doi: 10.1039/d6ra01511e (PMC13650539; doi:10.1039/d6ra01511e)
Supplement: Supplementary file 1 [file RA-016-D6RA01511E-s001.pdf]

Supplementary Information (SI) for RSC Advances.
This journal is © The Royal Society of Chemistry

Supporting Information

Synthesis of Withaferin A Derivatives and Exploratory Evaluation of Weight Reduction and Fasting Blood Glucose in DIO Mice

Zhiming Yan,[‡1] *Mingming Fan,*[‡1] *Xinyi Liang,*[‡1] *Changhong Wei,*[2*] *Yiheng Zhao,*[3] *Hongyu Li,*[3] *Tingzhuang Yi,*[3*] *and Shixiong Yang*[2*]

[1]Pharmaceutical College, Guangxi Medical University, Nanning 530021, Guangxi, PR China

[2]Department of Research & Clinical Laboratory/Department of Administrative Office, The Fifth Affiliated Hospital of Guangxi Medical University & The First People's Hospital of Nanning, Nanning 530022, Guangxi, PR China

[3]Department of Oncology, Affiliated Hospital of YouJiang Medical University For Nationalities, Guangxi Clinical Medical Research Center for Hepatobiliary Diseases, Guangxi Higher Education Engineering Research Center for Gastrointestinal Microecology and Health, Baise City Key Laboratory of Gastrointestinal Microecology and Health, Baise 533000, Guangxi, PR China

[‡] contributed equally.

Corresponding Authors

* Changhong Wei, E-mail: 258894453@qq.com

*Tingzhuang Yi, E-mail: yitingzhuang@ymun.edu.cn

Shixiong Yang, E-mail: 524651906@qq.com

CONTENTS

1: Characteristic data of the compounds..S3

II: ^{1}H and ^{13}C NMR Spectra S9

III: HRMS spectra S20

I: Characteristic data of the compounds

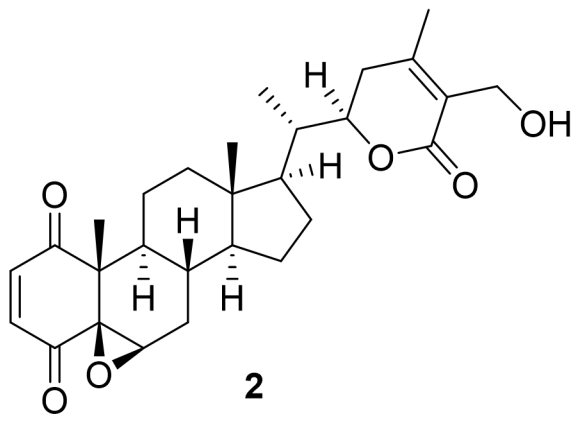

2

Compound **2:** **^{1}H NMR** (600 MHz, Chloroform-*d*) δ 6.91 – 6.82 (m, 2H), 4.50 – 4.25 (m, 3H), 3.42 (d, *J* = 2.6 Hz, 1H), 2.89 (d, *J* = 6.7 Hz, 1H), 2.50 (dd, *J* = 17.8, 13.3 Hz, 1H), 2.16 (dd, *J* = 15.0, 2.9 Hz, 1H), 2.04 (s, 3H), 2.02 – 1.93 (m, 3H), 1.66 (q, *J* = 8.4, 3H), 1.64 – 1.55 (m, 2H), 1.48 – 1.41 (m, 2H), 1.37 (s, 3H), 1.36 – 1.23 (m, 3H), 1.20 – 1.09 (m, 2H), 1.01 (dd, *J* = 6.7, 1.6 Hz, 3H), 0.72 (d, *J* = 1.6 Hz, 3H). **^{13}C NMR** (150 MHz, Chloroform-*d*) δ 202.27, 194.00, 167.12, 152.95, 141.73, 139.28, 125.82, 78.81, 64.09, 63.65, 57.58, 55.71, 52.20, 50.00, 43.78, 42.80, 39.55, 38.88, 30.65, 29.94, 29.76, 27.31, 24.37, 23.58, 20.16, 19.32, 13.46, 11.92. **HRMS (ESI)**: m/z calcd for $C28H37O6^{+}$ $[M + H]^{+}$: 469.2585; found: 469.2580.

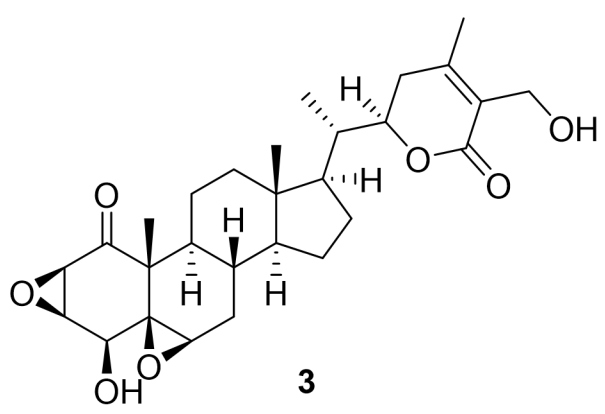

3

Compound **3:** **^{1}H NMR** (600 MHz, Chloroform-*d*) δ 4.44 – 4.31 (m, 3H), 3.91 (d, *J* = 3.7 Hz, 1H), 3.72 (t, *J* = 4.2 Hz, 1H), 3.57 (d, *J* = 4.5 Hz, 1H), 3.10 (d, *J* = 2.1 Hz, 1H), 3.04 – 2.92 (m, 2H), 2.49 (dd, *J* = 17.9, 13.3 Hz, 1H), 2.23 – 2.15 (m, 1H), 2.03 (s, 3H), 2.02 – 1.95 (m, 2H), 1.94 – 1.88 (m, 1H), 1.70 – 1.61 (m, 2H), 1.42 – 1.31 (m, 5H), 1.22 (s, 3H), 1.19 – 1.09 (m, 3H), 1.09 – 1.04 (m, 2H), 0.98 (d, *J* = 6.7 Hz, 3H), 0.65 (s, 3H). **^{13}C NMR** (150 MHz, Chloroform-*d*) δ 205.41, 166.16, 152.14, 124.76, 77.84, 73.99, 62.93, 58.80, 56.52, 55.14, 54.53, 53.92, 50.96, 47.74, 41.78, 39.83, 37.93, 37.88, 30.13, 28.94, 28.65, 26.45, 23.37, 19.25, 19.17, 13.76, 12.49, 10.59. **HRMS (ESI)**: m/z calcd for $C_{28}H_{39}O_{7}^{+}$ $[M + H]^{+}$: 487.2690; found: 487.2687.

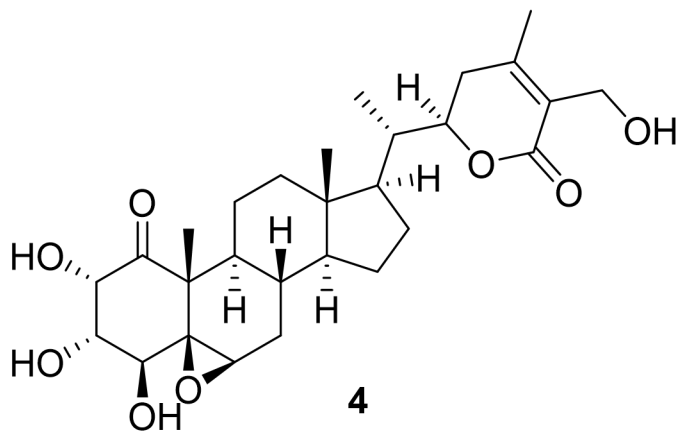

4

Compound **4:** ^{1}**H NMR** (600 MHz, Chloroform-*d*) δ 4.95 (t, *J* = 2.9 Hz, 1H), 4.42 (dt, *J* = 13.3, 3.5 Hz, 1H), 4.35 (s, 2H), 4.31 (t, *J* = 3.5 Hz, 1H), 3.76 (d, *J* = 3.4 Hz, 1H), 3.58 (d, *J* = 3.9 Hz, 1H), 3.28 (d, *J* = 2.6 Hz, 1H), 2.85 (s, 1H), 2.56 (dd, *J* = 17.8, 13.3 Hz, 1H), 2.10 (d, *J* = 14.8 Hz, 1H), 2.03 (s, 3H), 2.01 – 1.91 (m, 3H), 1.62 (d, *J* = 2.5 Hz, 2H), 1.47 (s, 3H), 1.45 – 1.41 (m, 1H), 1.37 – 1.30 (m, 2H), 1.28 – 1.22 (m, 3H), 1.17 – 1.09 (m, 2H), 1.03 (d, *J* = 6.6 Hz, 3H), 0.99 – 0.94 (m, 1H), 0.69 (s, 3H).

^{13}**C NMR** (150 MHz, Chloroform-*d*) δ 211.55, 166.34, 152.45, 124.64, 77.90, 75.59, 71.53, 70.56, 65.07, 63.36, 56.50, 54.96, 51.24, 48.65, 42.15, 41.81, 38.66, 37.95, 30.05, 28.92, 27.56, 26.32, 23.39, 22.18, 19.20, 17.07, 12.42, 10.84. **HRMS (ESI)**: m/z calcd for $C_{28}H_{41}O_8^+$ [M + H]$^+$: 505.2796; found: 505.2791.

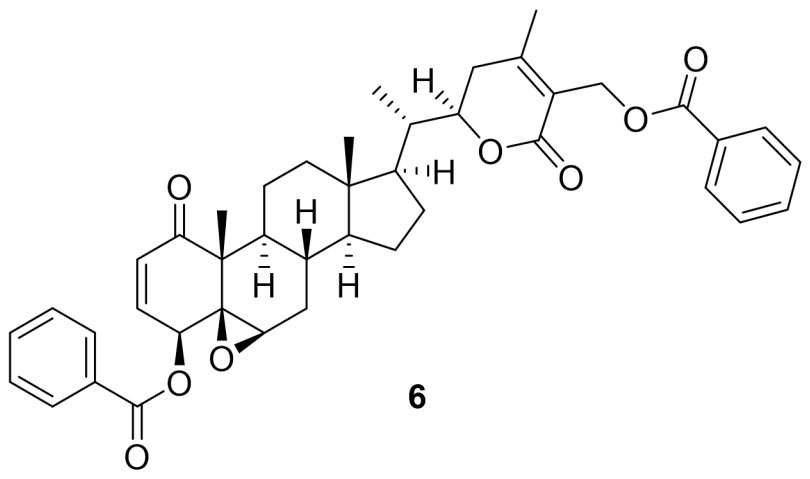

6

Compound **6:** ^{1}**H NMR** (600 MHz, Chloroform-*d*) δ 8.01 (d, *J* = 7.8 Hz, 2H), 7.94 (d, *J* = 7.8 Hz, 2H), 7.57 – 7.51 (m, 2H), 7.41 (q, *J* = 7.4 Hz, 4H), 7.14 (dd, *J* = 9.9, 6.1 Hz, 1H), 6.29 (d, *J* = 9.8 Hz, 1H), 5.14 (s, 2H), 4.93 (d, *J* = 6.1 Hz, 1H), 4.42 (d, *J* = 13.3 Hz, 1H), 3.29 (s, 1H), 2.53 (dd, *J* = 18.0, 13.2 Hz, 1H), 2.21 – 2.16 (m, 1H), 2.13 (s, 3H), 2.07 – 1.91 (m, 3H), 1.74 – 1.59 (m, 3H), 1.51 (s, 3H), 1.40 – 1.28 (m, 4H), 1.14 (dd, *J* = 12.2, 5.5 Hz, 1H), 1.07 (dd, *J* = 12.1, 9.3 Hz, 1H), 1.00 (d, *J* = 6.6 Hz, 3H), 0.93 (dq, *J* = 16.0, 6.8, 5.6 Hz, 2H), 0.86 (t, *J* = 6.9 Hz, 1H), 0.70 (s, 3H). ^{13}**C NMR** (150 MHz, $CDCl_3$) δ 201.29, 166.52, 165.91, 165.36, 157.19, 139.91, 134.19, 133.49, 133.10, 130.10, 129.84, 129.81, 129.40, 128.60, 128.42, 122.14, 78.31, 72.69, 61.26, 60.48, 58.55, 56.19, 52.04, 48.27, 44.30, 42.70, 39.30, 38.89, 31.25, 30.29, 29.79, 29.72,

29.41, 27.43, 24.35, 21.50, 20.83, 16.01, 13.46, 11.69. **HRMS (ESI)**: m/z calcd for $C_{42}H_{47}O_8^+$ [M + H]$^+$: 679.3265; found: 679.3247.

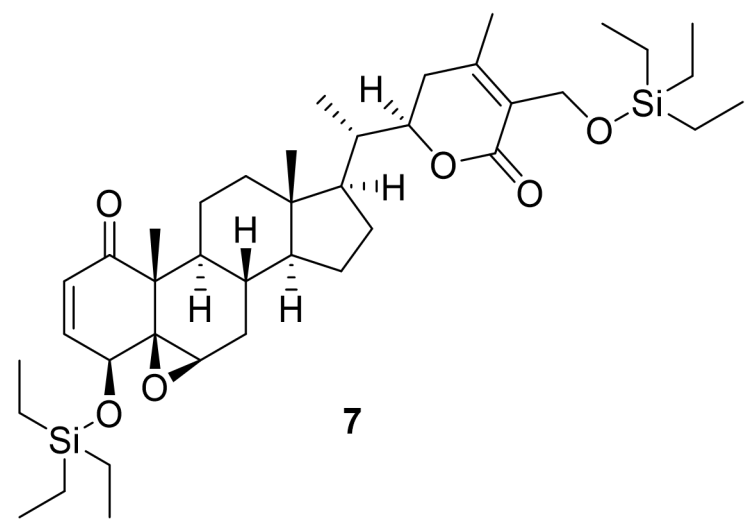

7

Compound **7:** ^{1}**H NMR** (600 MHz, Chloroform-*d*) δ 6.89 (dd, *J* = 10.4, 6.0 Hz, 1H), 6.13 (d, *J* = 9.3 Hz, 1H), 4.49 (d, *J* = 11.7 Hz, 1H), 4.36 (d, *J* = 12.0 Hz, 2H), 3.56 (d, *J* = 6.0 Hz, 1H), 3.06 (s, 1H), 2.44 (dd, *J* = 17.7, 13.4 Hz, 1H), 2.17 – 2.09 (m, 1H), 2.05 (s, 3H), 1.99 – 1.89 (m, 4H), 1.70 – 1.57 (m, 4H), 1.50 – 1.41 (m, 2H), 1.38 (s, 3H), 1.35 (s, 2H), 1.26 (s, 3H), 1.03 (t, *J* = 11.4 Hz, 3H), 0.93 (dq, *J* = 15.0, 8.2 Hz, 18H), 0.66 (s, 3H), 0.61 (q, *J* = 8.1 Hz, 6H), 0.58 – 0.54 (m, 6H). ^{13}**C NMR** (150 MHz, $CDCl_3$) δ 202.30, 165.89, 154.68, 143.74, 132.18, 126.01, 78.23, 71.22, 63.51, 59.85, 56.73, 56.26, 52.07, 48.20, 44.43, 42.65, 39.37, 38.88, 32.02, 31.59, 31.54, 30.22, 30.16, 29.90, 29.79, 29.45, 29.41, 27.40, 24.40, 22.78, 21.22, 20.56, 16.33, 14.21, 13.43, 11.61, 6.86, 6.73, 4.96, 4.43. **HRMS (ESI)**: m/z calcd for $C_{40}H_{66}NaO_6Si_2^+$ [M + Na]$^+$: 721.4290; found: 721.5064.

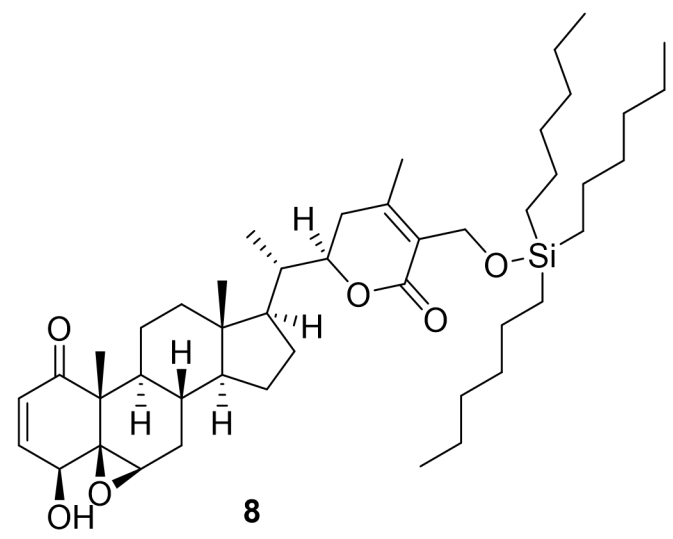

8

Compound **8:** ^{1}**H NMR** (600 MHz, Chloroform-*d*) δ 6.92 (dd, *J* = 10.0, 5.9 Hz, 1H), 6.19 (d, *J* = 10.0 Hz, 1H), 4.48 (d, *J* = 11.7 Hz, 1H), 4.36 (t, *J* = 13.2 Hz, 2H), 3.74 (d, *J* = 5.9 Hz, 1H), 3.22 (s, 1H), 2.45 (dd, *J* = 17.7, 13.2 Hz, 1H), 2.14 (dt, *J* = 15.2, 2.9 Hz, 1H), 2.05 (s, 3H), 1.96 (ddt, *J* = 21.0, 13.1, 3.7 Hz, 3H), 1.81 (dt, *J* = 14.4, 3.7 Hz, 1H), 1.70 – 1.59 (m, 2H), 1.53 – 1.43 (m, 2H), 1.40 (s, 3H), 1.27 (m, 30H), 1.17 – 1.03 (m, 3H), 0.97 (d, *J* = 6.7 Hz, 3H), 0.86 (t, *J* = 6.8 Hz, 9H), 0.69 (s, 3H), 0.60 – 0.59 (m,

6H). **^{13}C NMR** (150 MHz, Chloroform-*d*) δ 202.40, 165.89, 154.55, 142.07, 132.44, 126.06, 78.21, 70.03, 64.01, 62.55, 56.78, 56.19, 52.13, 47.82, 44.26, 42.68, 39.45, 38.91, 33.45, 31.68, 31.29, 30.18, 29.89, 27.41, 24.39, 23.25, 22.74, 22.19, 20.64, 17.47, 14.27, 13.64, 13.46, 11.72. **HRMS (ESI)**: m/z calcd for $C_{46}H_{77}O_6Si^+$ $[M + H]^+$: 753.5484; found: 753.5481.

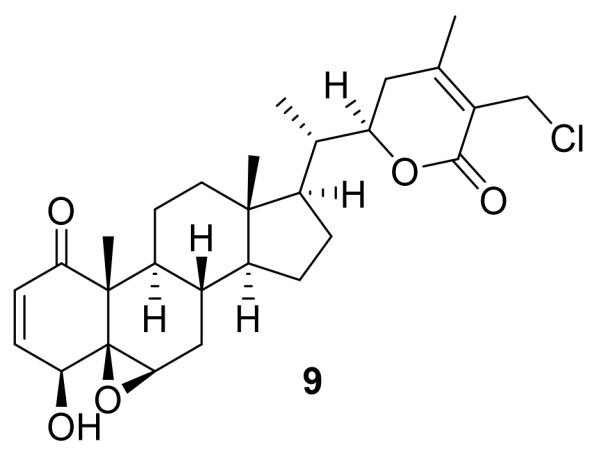

9

Compound **9:** **^{1}H NMR** (600 MHz, Chloroform-*d*) δ 6.48 (dd, *J* = 10.4, 2.5 Hz, 1H), 6.01 (dd, *J* = 10.4, 2.1 Hz, 1H), 4.49 – 4.37 (m, 3H), 3.74 (s, 1H), 3.14 (d, *J* = 4.7 Hz, 1H), 2.51 (dd, *J* = 18.0, 13.1 Hz, 1H), 2.36 – 2.28 (m, 1H), 2.10 (s, 3H), 2.03 – 1.97 (m, 2H), 1.95 – 1.88 (m, 1H), 1.70 – 1.63 (m, 3H), 1.44 – 1.33 (m, 4H), 1.26 (s, 3H), 1.25 (dd, *J* = 4.4, 2.7 Hz, 3H), 1.11 – 1.06 (m, 2H), 0.97 (d, *J* = 6.7 Hz, 3H), 0.68 (s, 3H). **^{13}C NMR** (150 MHz, Chloroform-*d*) δ 200.25, 164.78, 156.22, 142.94, 127.86, 124.22, 78.26, 66.27, 58.60, 57.34, 55.44, 51.88, 45.98, 43.29, 39.49, 39.09, 38.82, 37.22, 35.18, 30.20, 27.37, 24.09, 22.80, 20.69, 18.55, 13.43, 11.94, 10.06. **HRMS (ESI)**: m/z calcd for $C_{28}H_{38}ClO_5^+$ $[M + H]^+$: 489.2402; found: 489.2397.

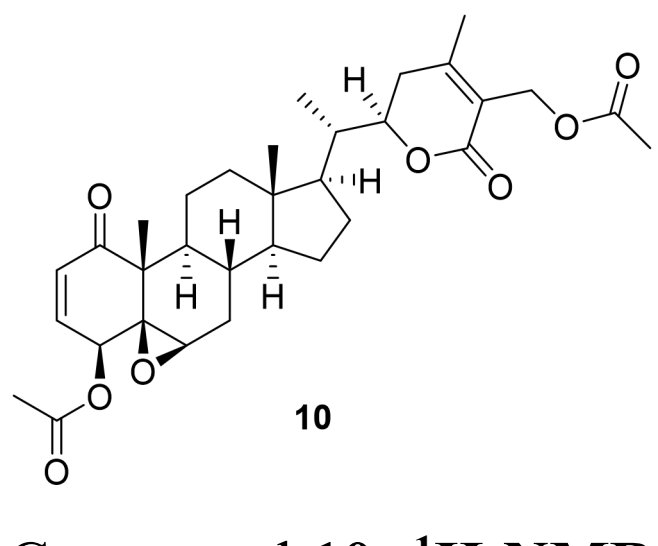

10

Compound **10:** **^{1}H NMR** (600 MHz, Chloroform-*d*) δ 7.02 (ddd, *J* = 9.9, 6.1, 1.0 Hz, 1H), 6.24 (dd, *J* = 9.8, 1.0 Hz, 1H), 4.93 – 4.79 (m, 2H), 4.65 (d, *J* = 6.1 Hz, 1H), 4.39 (dt, *J* = 13.2, 3.5 Hz, 1H), 3.21 (s, 1H), 2.50 (dd, *J* = 17.9, 13.2 Hz, 1H), 2.16 (dt, *J* = 14.9, 2.7 Hz, 1H), 2.06 (s, 3H), 2.04 (s, 6H), 2.02 – 1.98 (m, 2H), 1.96 – 1.92 (m, 1H), 1.70 – 1.61 (m, 3H), 1.50 – 1.43 (m, 2H), 1.38 (s, 3H), 1.15 (dd, *J* = 12.3, 5.5 Hz, 1H),

1.08 – 1.02 (m, 2H), 0.98 (d, *J* = 6.6 Hz, 3H), 0.94 – 0.89 (m, 1H), 0.88 – 0.84 (m, 1H), 0.69 (s, 3H). **^{13}C NMR** (150 MHz, Chloroform-*d*) δ 201.30, 171.03, 170.23, 165.40, 157.10, 139.92, 134.01, 121.99, 78.30, 72.28, 61.11, 60.39, 58.13, 56.20, 52.02, 48.24, 44.25, 42.69, 39.29, 38.88, 31.24, 30.22, 29.69, 27.43, 24.36, 21.39, 21.04, 20.93, 20.72, 15.81, 13.45, 11.69. **HRMS (ESI)**: m/z calcd for $C_{32}H_{43}O_8^+$ $[M + H]^+$: 555.2952; found: 555.2950.

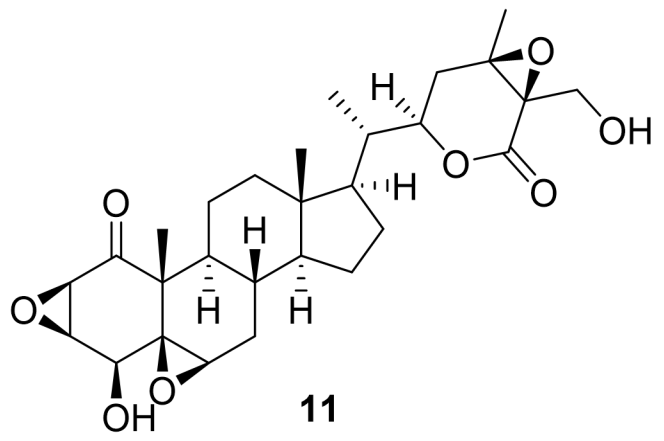

11

Compound **11:** **^{1}H NMR** (600 MHz, Chloroform-*d*) δ 4.55 (dt, *J* = 11.4, 3.7 Hz, 1H), 4.13 (d, *J* = 12.5 Hz, 1H), 3.91 (d, *J* = 3.8 Hz, 1H), 3.79 (d, *J* = 12.6 Hz, 1H), 3.72 (t, *J* = 4.1 Hz, 1H), 3.57 (d, *J* = 4.5 Hz, 1H), 3.11 (d, *J* = 1.4 Hz, 1H), 2.21 – 2.16 (m, 1H), 2.05 – 1.96 (m, 2H), 1.93 – 1.86 (m, 2H), 1.73 (ddd, *J* = 12.9, 6.7, 3.8 Hz, 1H), 1.68 – 1.62 (m, 1H), 1.56 (s, 3H), 1.41 – 1.31 (m, 6H), 1.21 (s, 3H), 1.18 – 1.10 (m, 3H), 1.03 (dd, *J* = 12.2, 9.1 Hz, 3H), 0.90 (d, *J* = 6.6 Hz, 3H), 0.63 (s, 3H). **^{13}C NMR** (150 MHz,Chloroform-*d*) δ 205.41, 169.25, 75.86, 74.02, 62.94, 62.37, 59.69, 59.31, 58.83, 55.12, 54.51, 53.92, 51.00, 47.73, 41.79, 39.82, 37.91, 37.61, 30.11, 28.65, 28.28, 26.37, 23.33, 19.25, 16.55, 13.77, 12.14, 10.59.**HRMS (ESI)**: m/z calcd for $C_{28}H_{39}O_8^+$ $[M + H]^+$: 503.2639; found: 503.2634.

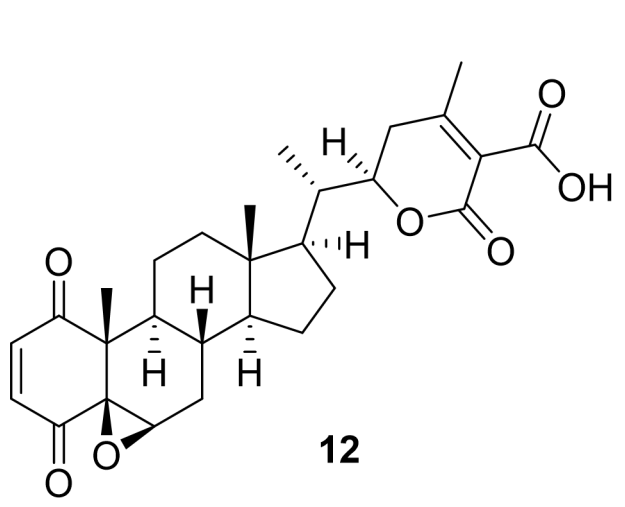

12

Compound **12:** **^{1}H NMR** (600 MHz, Chloroform-*d*) δ 6.92 – 6.80 (m, 2H), 4.48 (dt, *J* = 13.5, 3.4 Hz, 1H), 3.43 (d, *J* = 2.5 Hz, 1H), 2.72 (t, *J* = 16.4 Hz, 1H), 2.32 (dd, *J* = 14.9, 6.9 Hz, 1H), 2.25 – 2.14 (m, 2H), 2.08 – 1.95 (m, 4H), 1.71 – 1.56 (m, 6H), 1.48

– 1.41 (m, 3H), 1.38 (s, 3H), 1.03 (d, *J* = 6.7 Hz, 3H), 0.87 (t, *J* = 7.1 Hz, 3H), 0.73 (s, 3H). **^{13}C NMR** (150 MHz, Chloroform-*d*) δ 202.28, 193.93, 141.71, 139.33, 130.15, 130.02, 78.96, 70.67, 64.08, 63.62, 55.67, 52.05, 50.00, 43.76, 42.90, 39.55, 38.59, 30.63, 27.36, 24.36, 23.60, 22.83, 19.36, 14.27, 13.40, 11.96. **HRMS (ESI)**: m/z calcd for $C_{28}H_{35}O_7^+$ $[M + H]^+$: 483.2377; found: 483.2370.

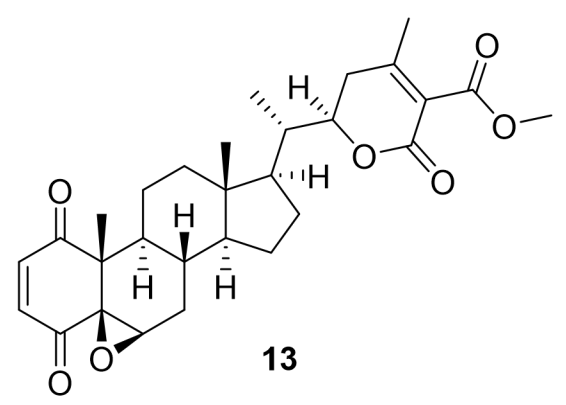

13

Compound **13:** **^{1}H NMR** (600 MHz, Chloroform-*d*) δ 6.89 – 6.82 (m, 2H), 4.45 (dt, *J* = 13.1, 3.4 Hz, 1H), 3.85 (s, 3H), 3.42 (d, *J* = 2.5 Hz, 1H), 2.52 (dd, *J* = 18.0, 13.0 Hz, 1H), 2.21 (t, *J* = 7.7 Hz, 1H), 2.16 (d, *J* = 15.1 Hz, 1H), 2.08 (s, 3H), 2.07 – 1.94 (m, 6H), 1.68 – 1.57 (m, 3H), 1.46 – 1.41 (m, 2H), 1.37 (s, 3H), 1.01 (d, *J* = 6.6 Hz, 3H), 0.87 (t, *J* = 6.9 Hz, 3H), 0.71 (s, 3H).**^{13}C NMR** (150 MHz, $CDCl_3$) δ 202.26, 193.98, 165.59, 162.45, 157.84, 141.72, 139.28, 123.46, 78.94, 64.07, 63.63, 55.70, 52.67, 52.18, 49.99, 43.77, 42.82, 39.54, 38.79, 30.64, 27.33, 24.37, 23.57, 22.81, 21.64, 19.32, 14.25, 13.41, 11.91.**HRMS (ESI)**: m/z calcd for $C_{29}H_{37}O_7^+$ $[M + H]^+$: 497.2534; found: 497.2527.

II: ^{1}H and ^{13}C NMR Spectra

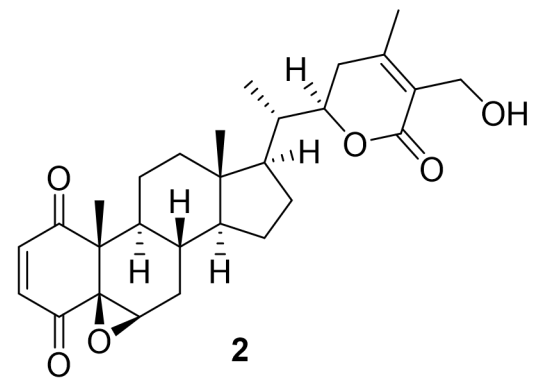

2

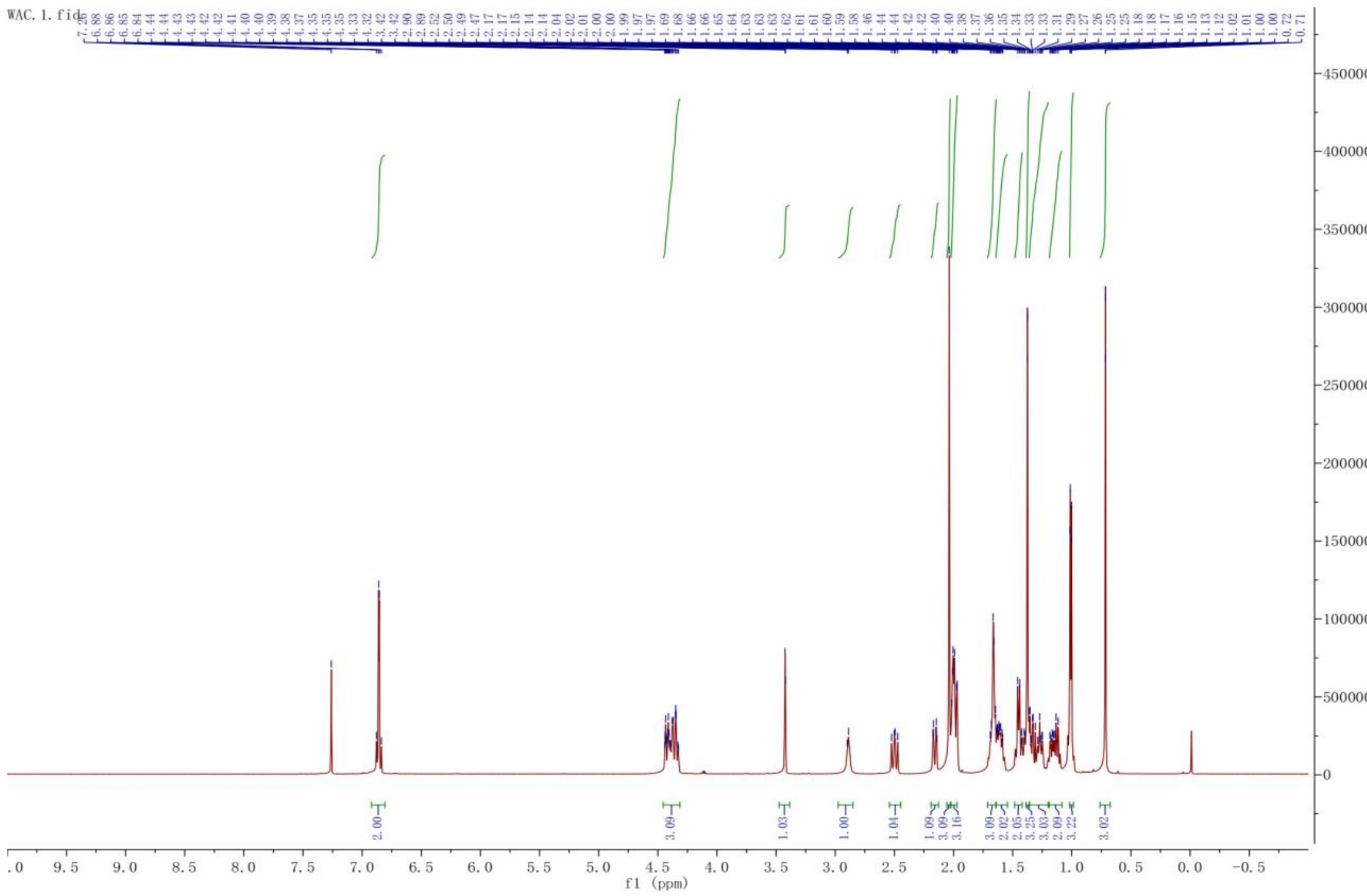

Figure S1. ^{1}H NMR (600 MHz, $CDCl_3$) spectrum of compound **2**

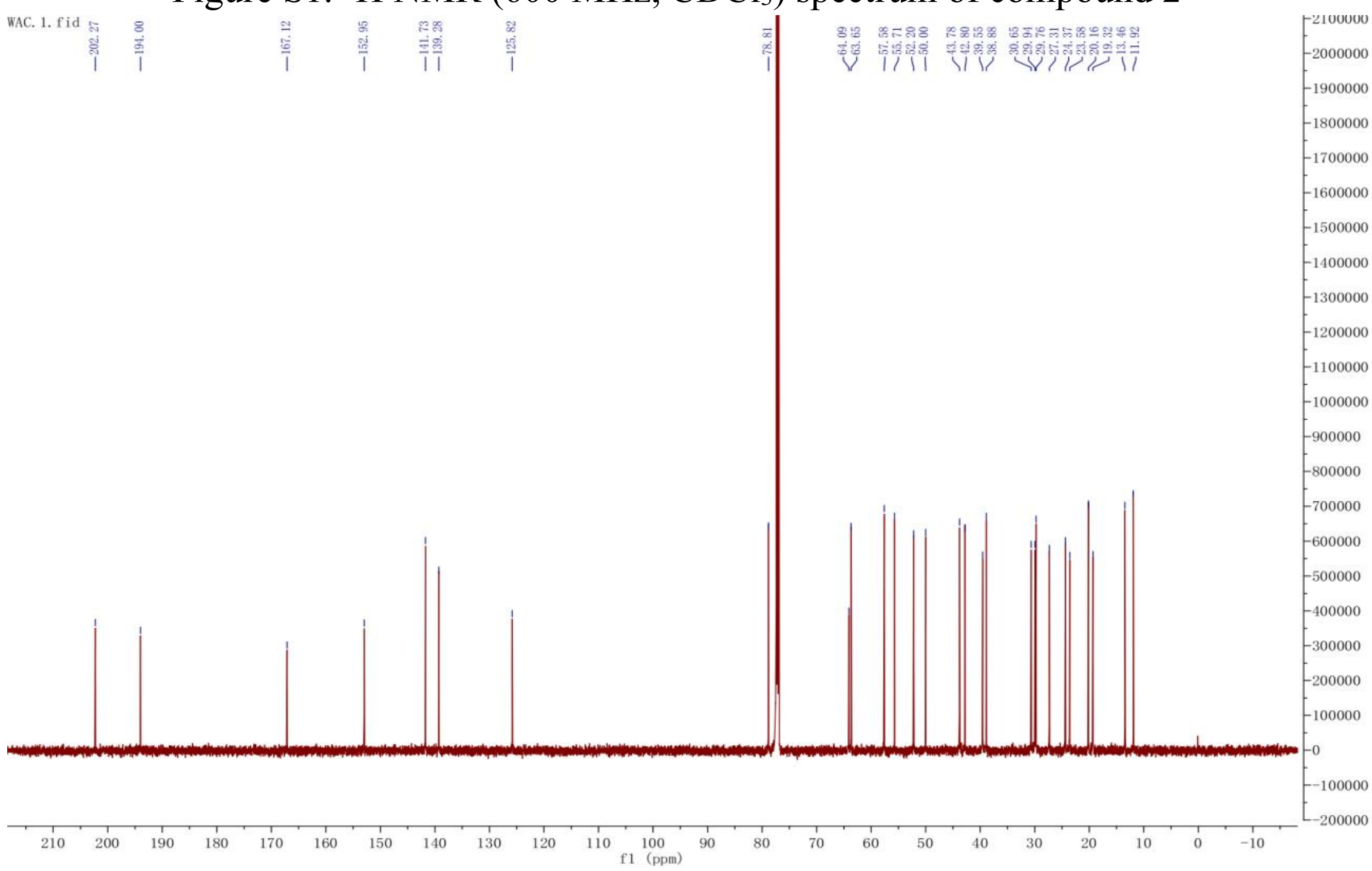

Figure S2. ^{13}C NMR (150 MHz, $CDCl_3$) spectrum of compound **2**

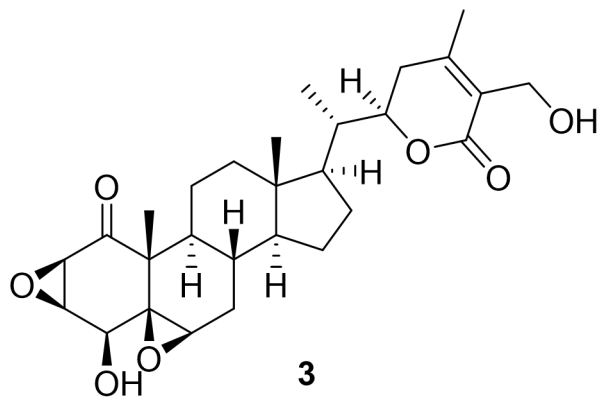

3

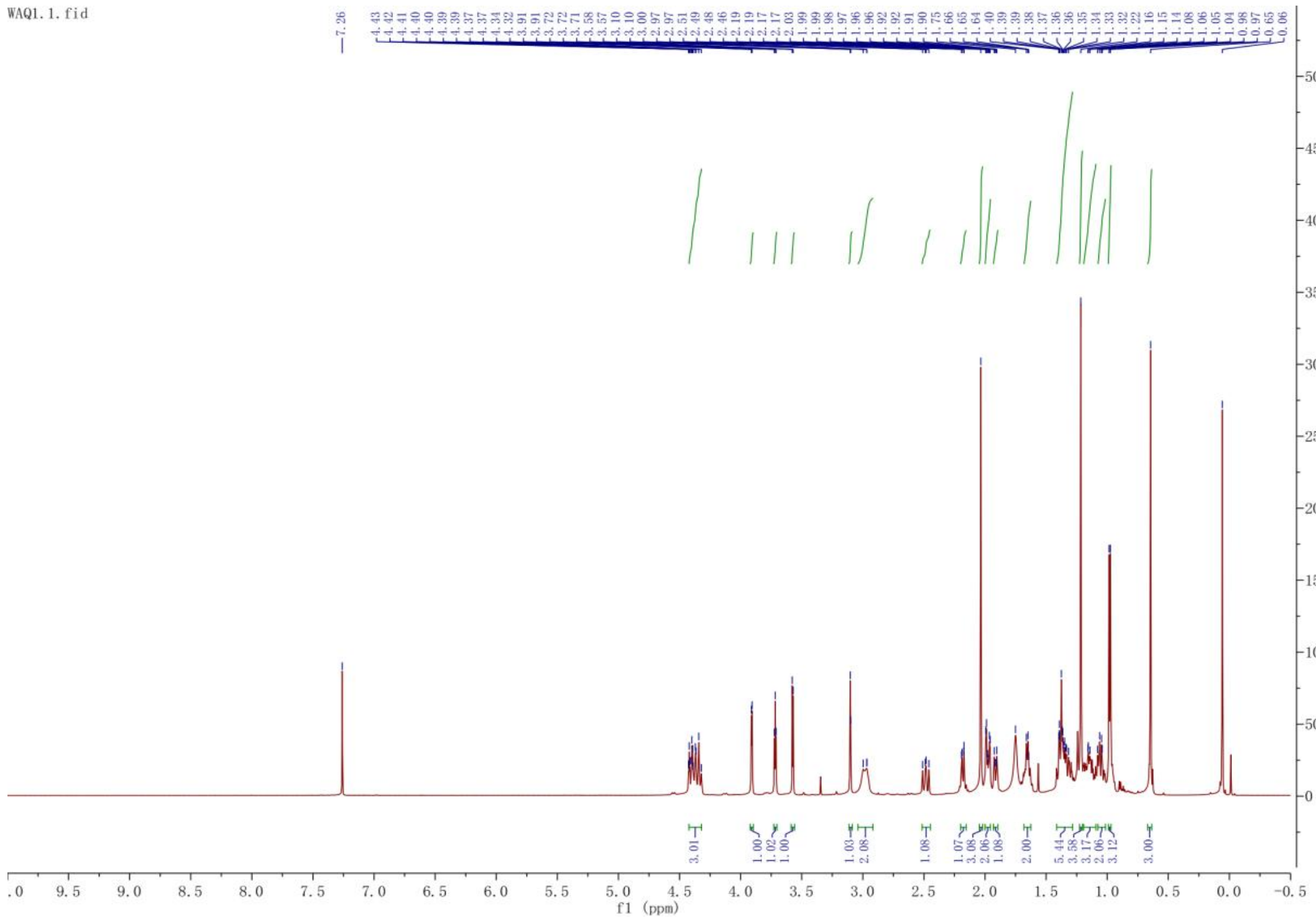

Figure S3. ^{1}H NMR (600 MHz, $CDCl_3$) spectrum of compound **3**

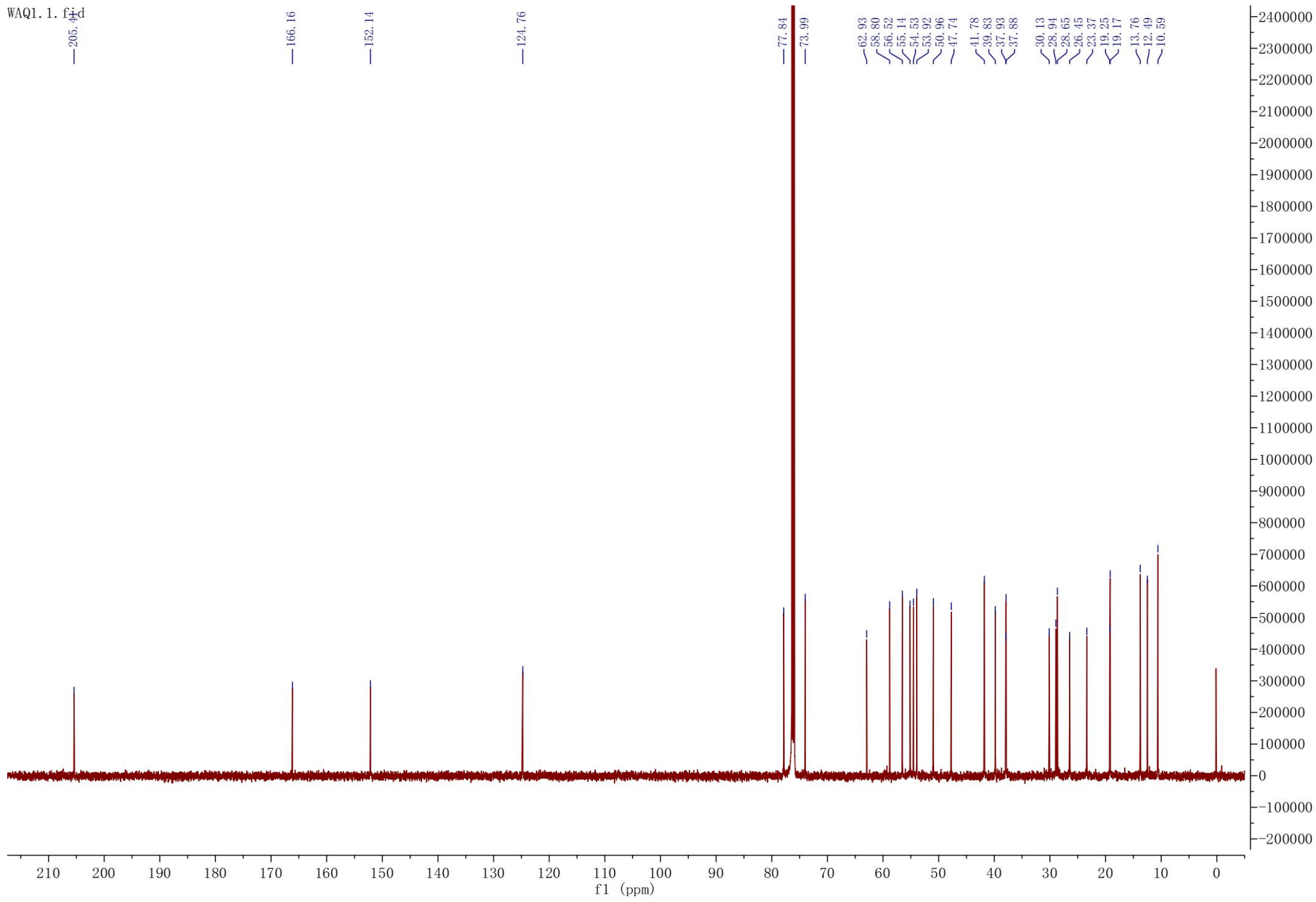

Figure S4. ^{13}C NMR (150 MHz, $CDCl_3$) spectrum of compound **3**

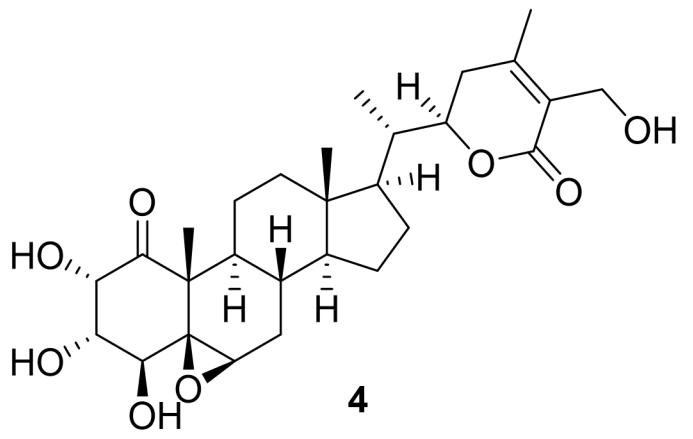

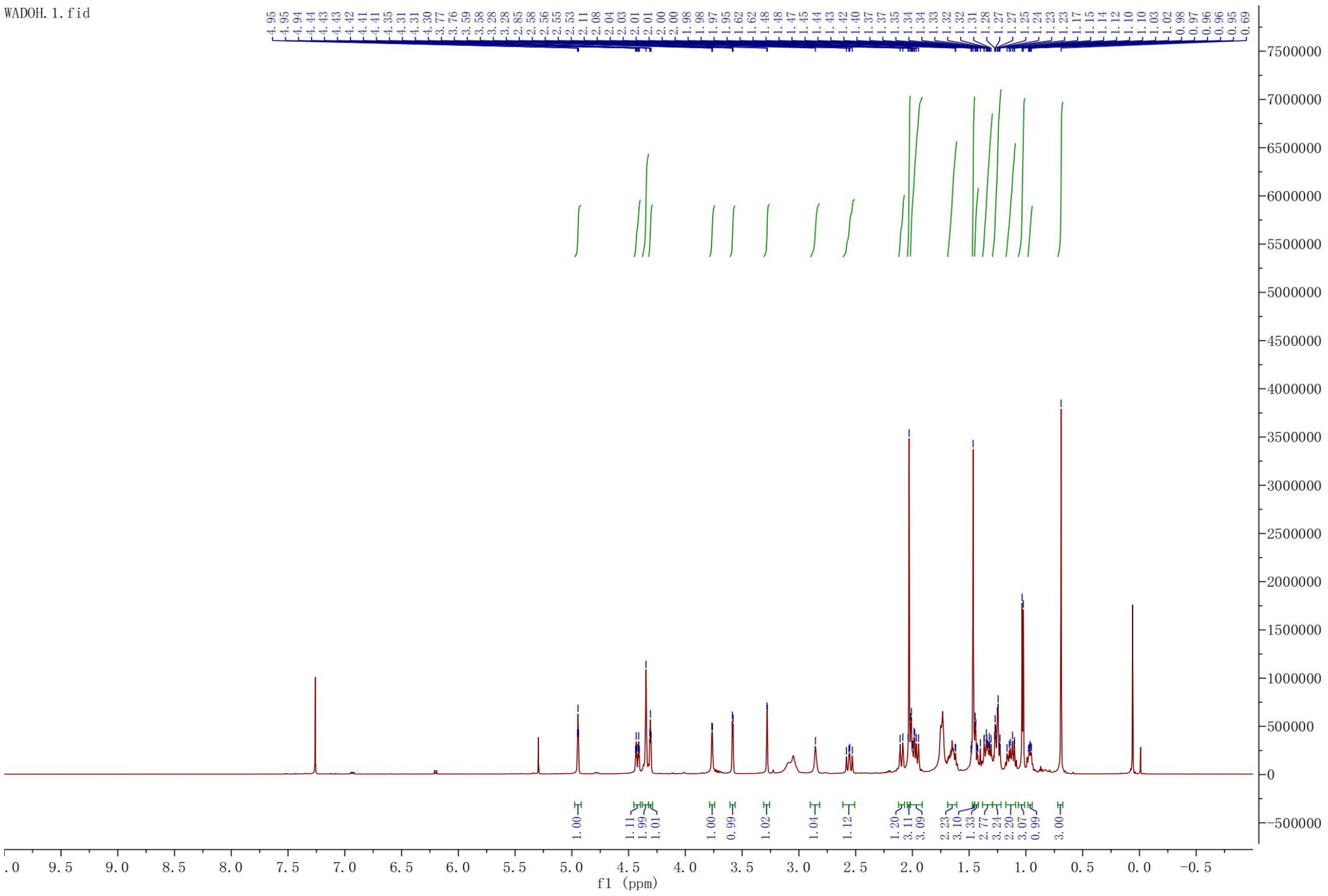

Figure S5. ^{1}H NMR (600 MHz, $CDCl_3$) spectrum of compound **4**

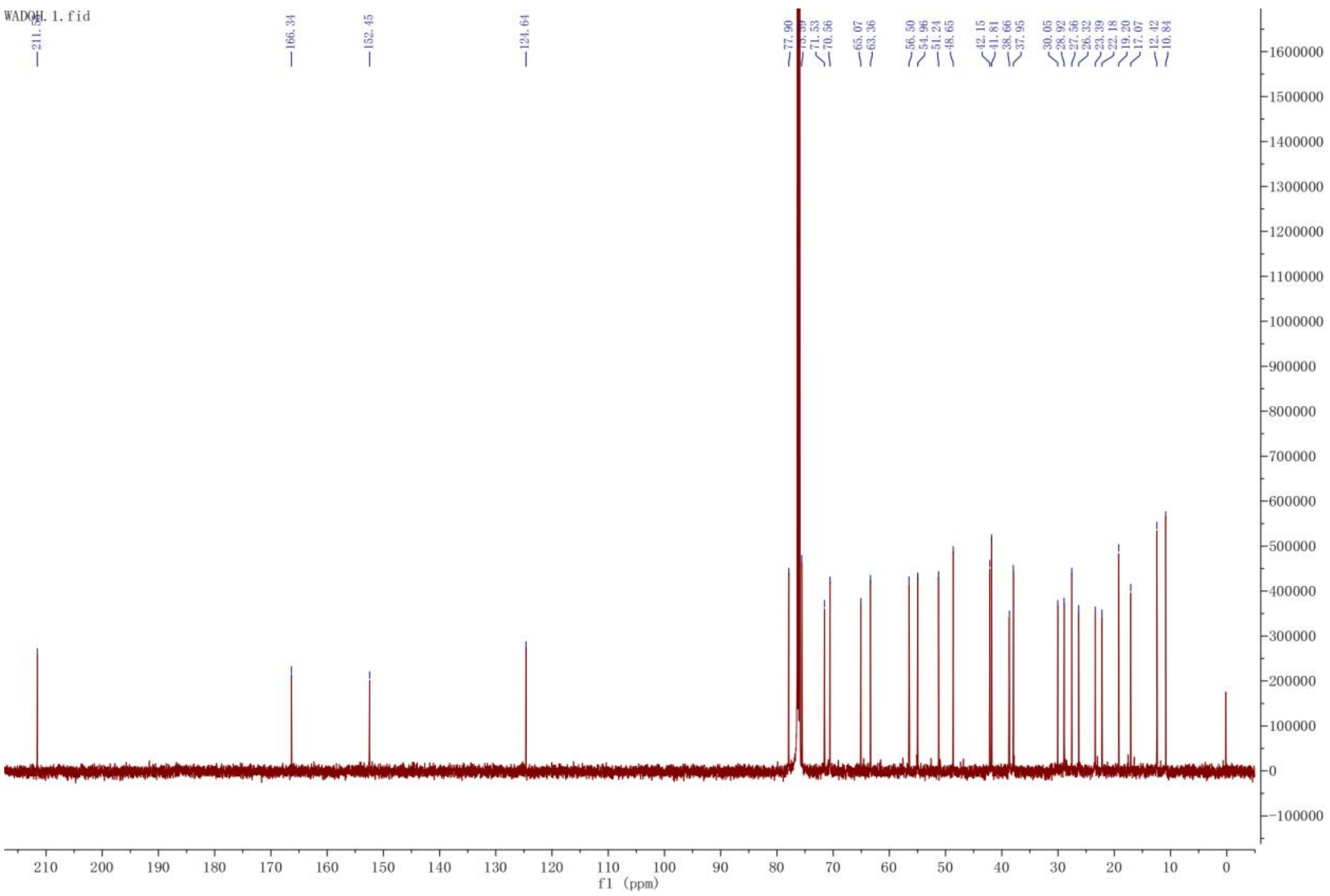

Figure S6. ^{13}C NMR (150 MHz, $CDCl_3$) spectrum of compound **4**

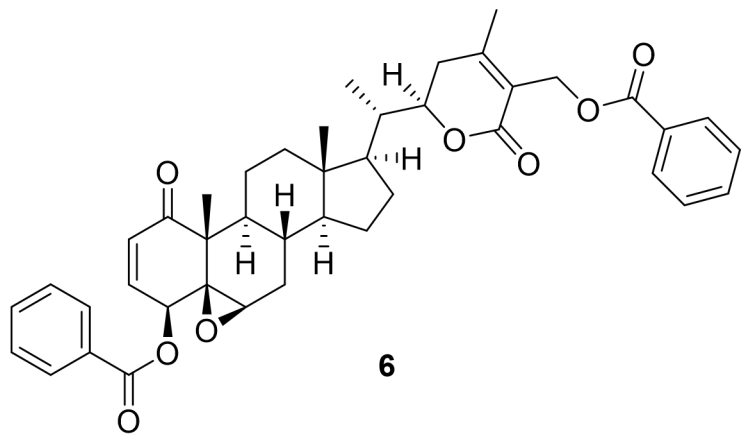

6

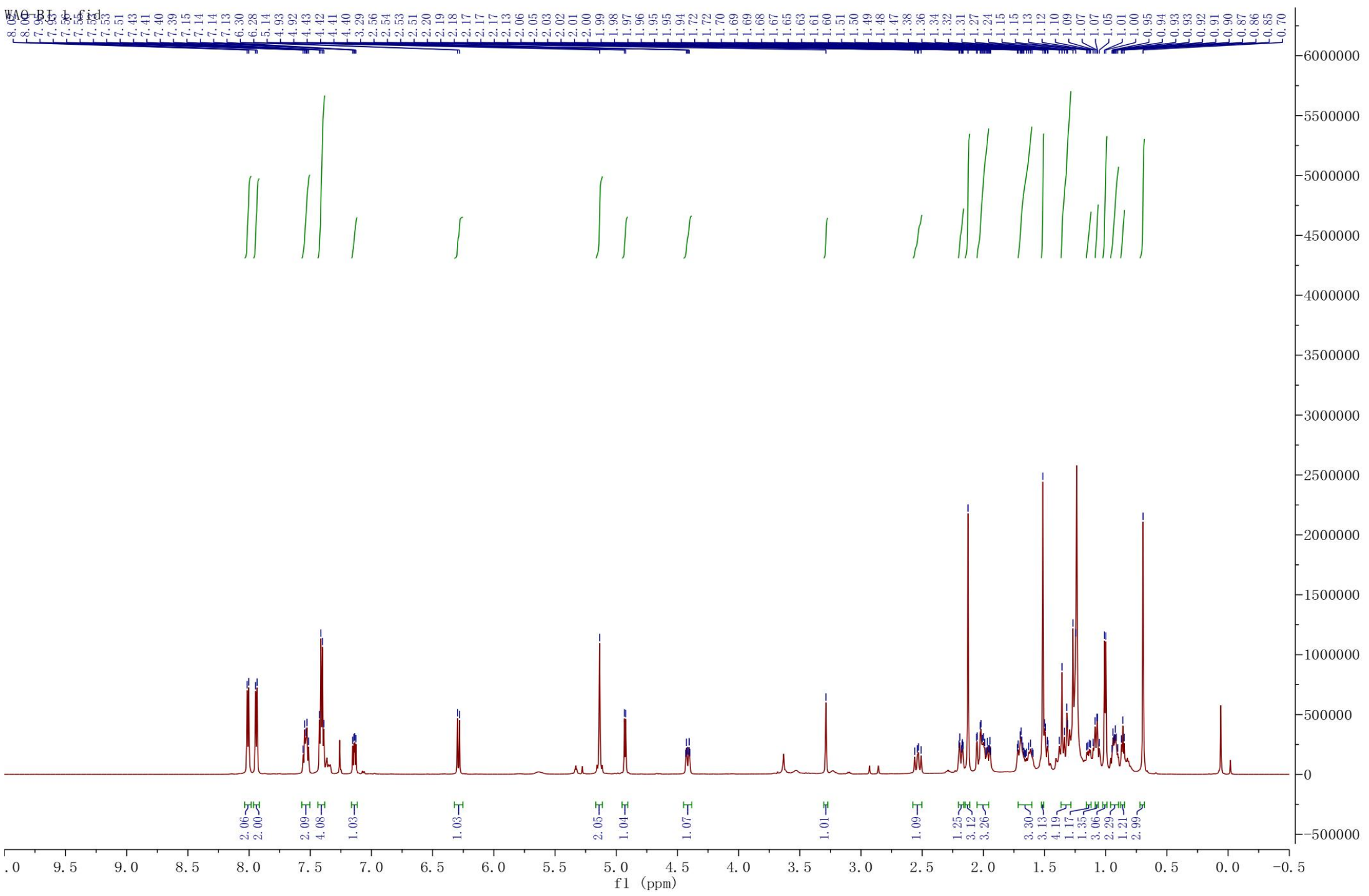

Figure S7. ^{1}H NMR (600 MHz, $CDCl_3$) spectrum of compound **6**

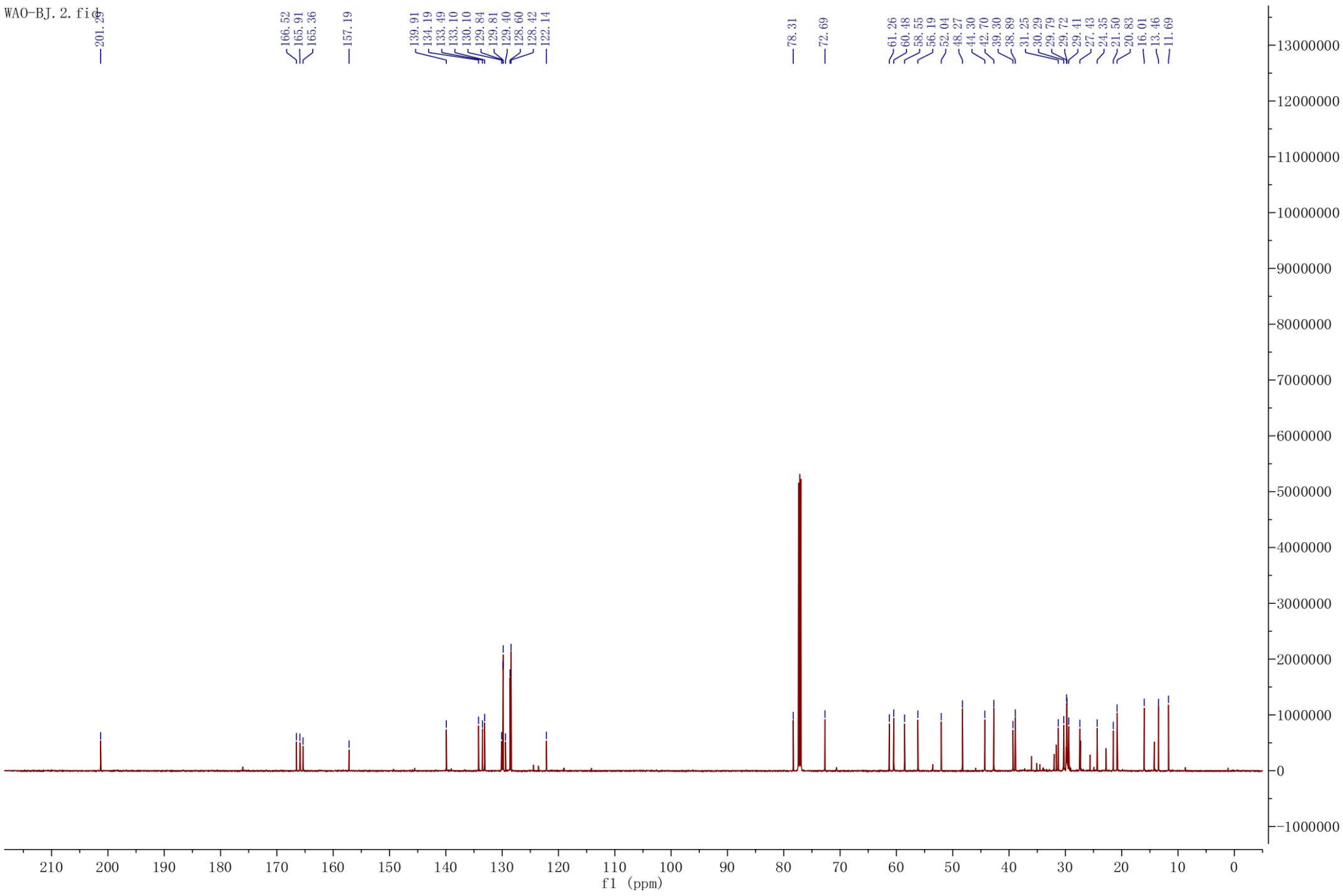

Figure S8. ^{13}C NMR (150 MHz, $CDCl_3$) spectrum of compound **6**

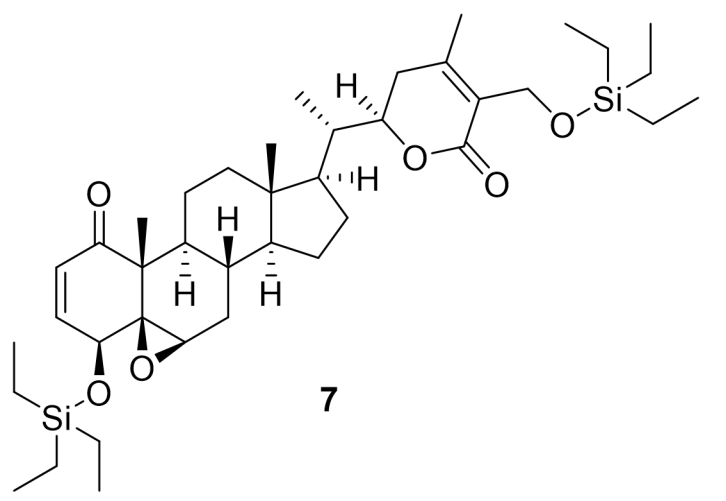

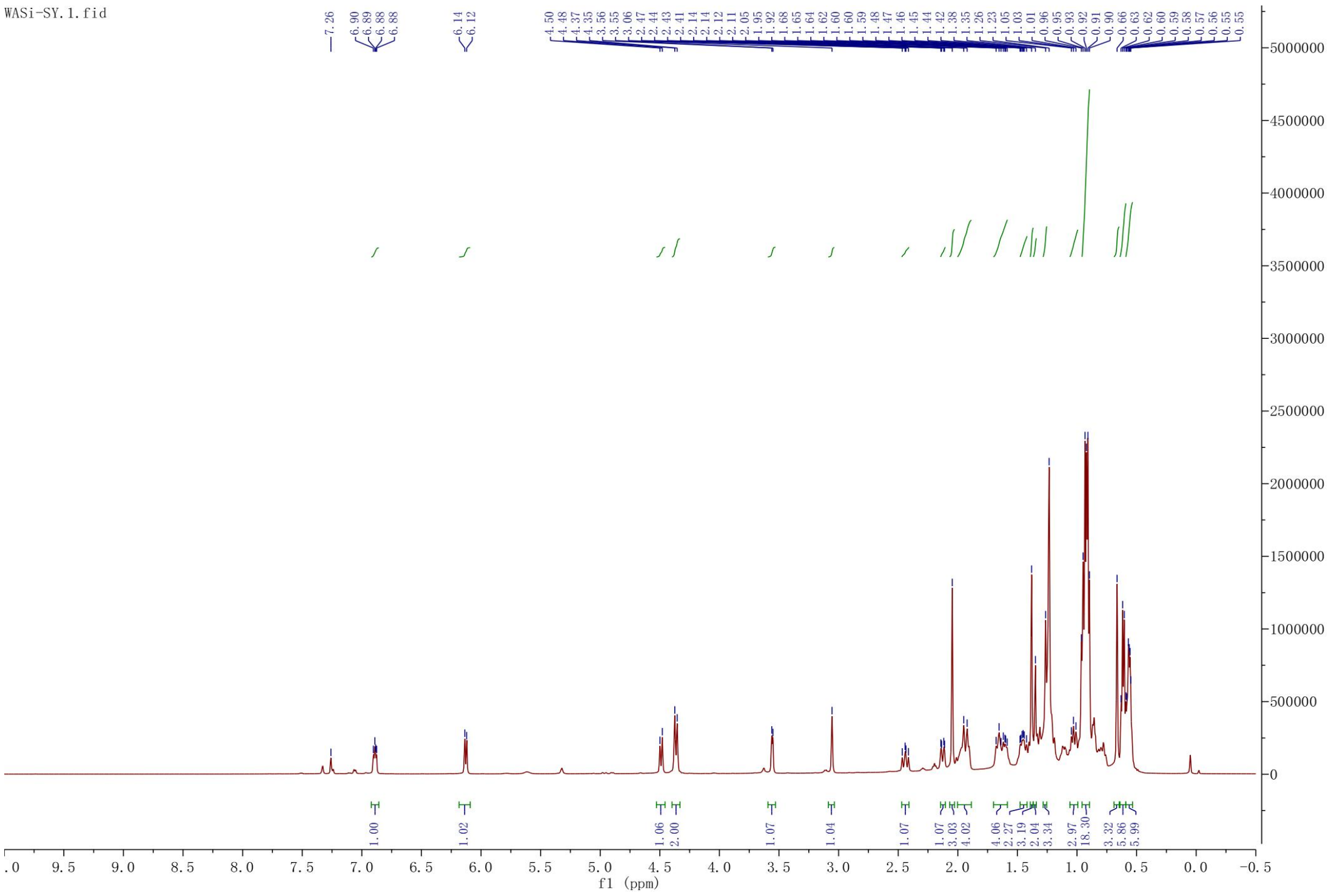

Figure S9. ^{1}H NMR (600 MHz, $CDCl_3$) spectrum of compound **7**

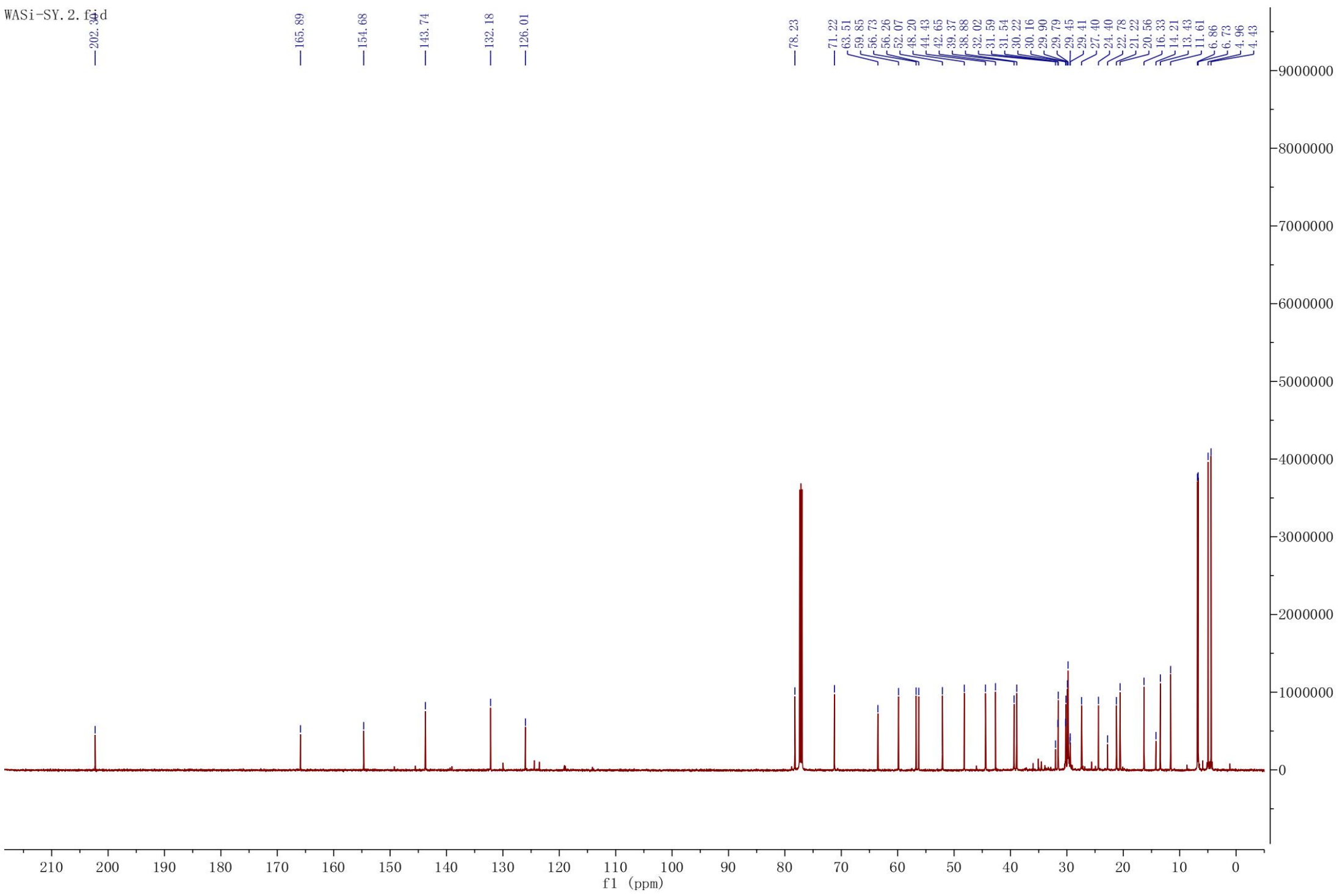

Figure S10. ^{13}C NMR (150 MHz, $CDCl_3$) spectrum of compound **7**

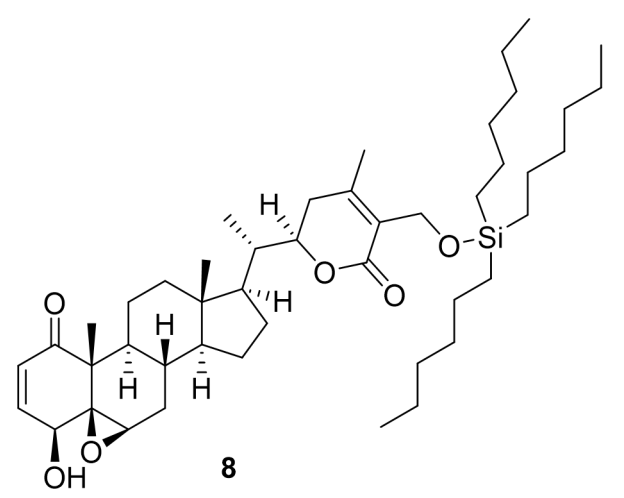

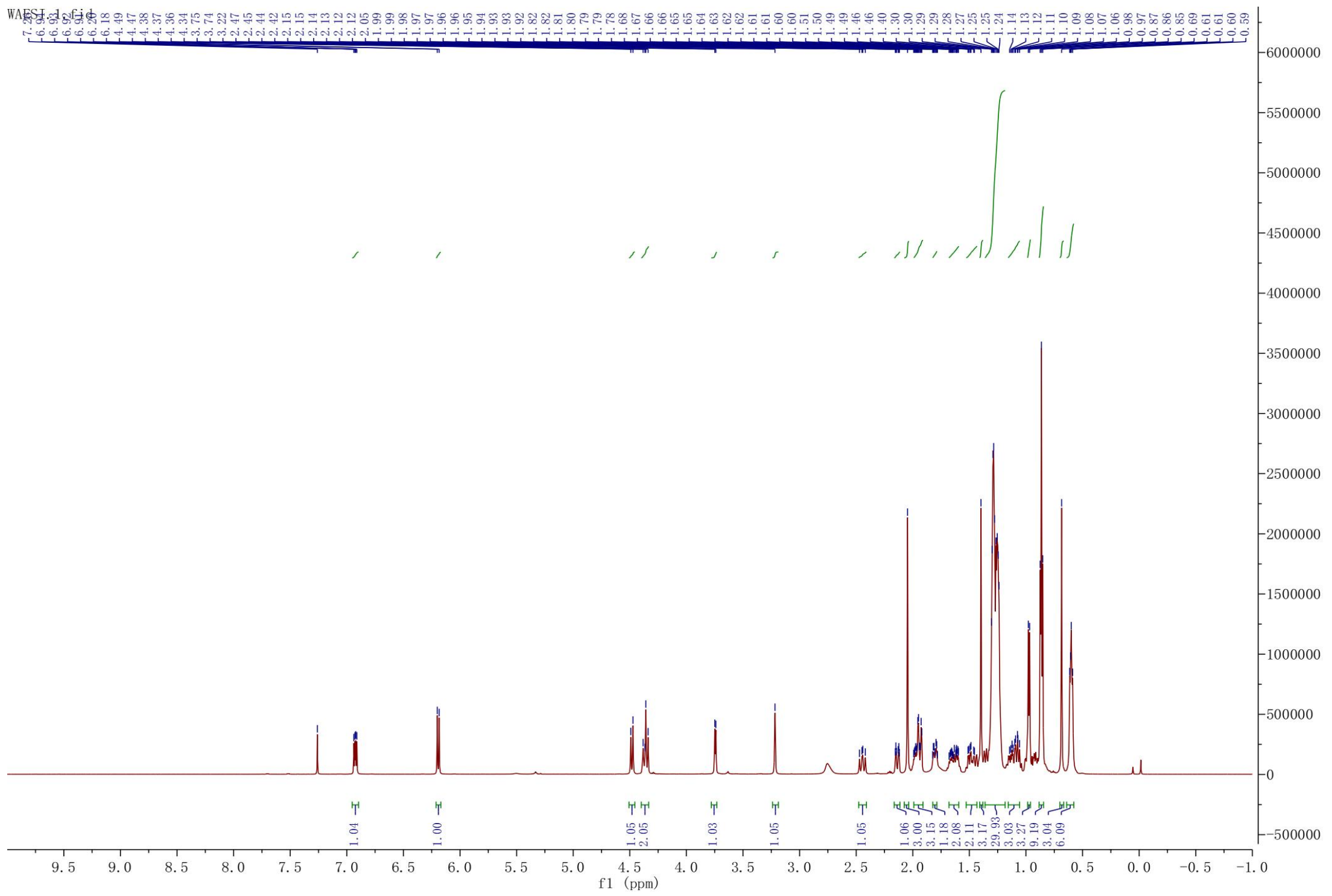

Figure S11. ^{1}H NMR (600 MHz, $CDCl_3$) spectrum of compound **8**

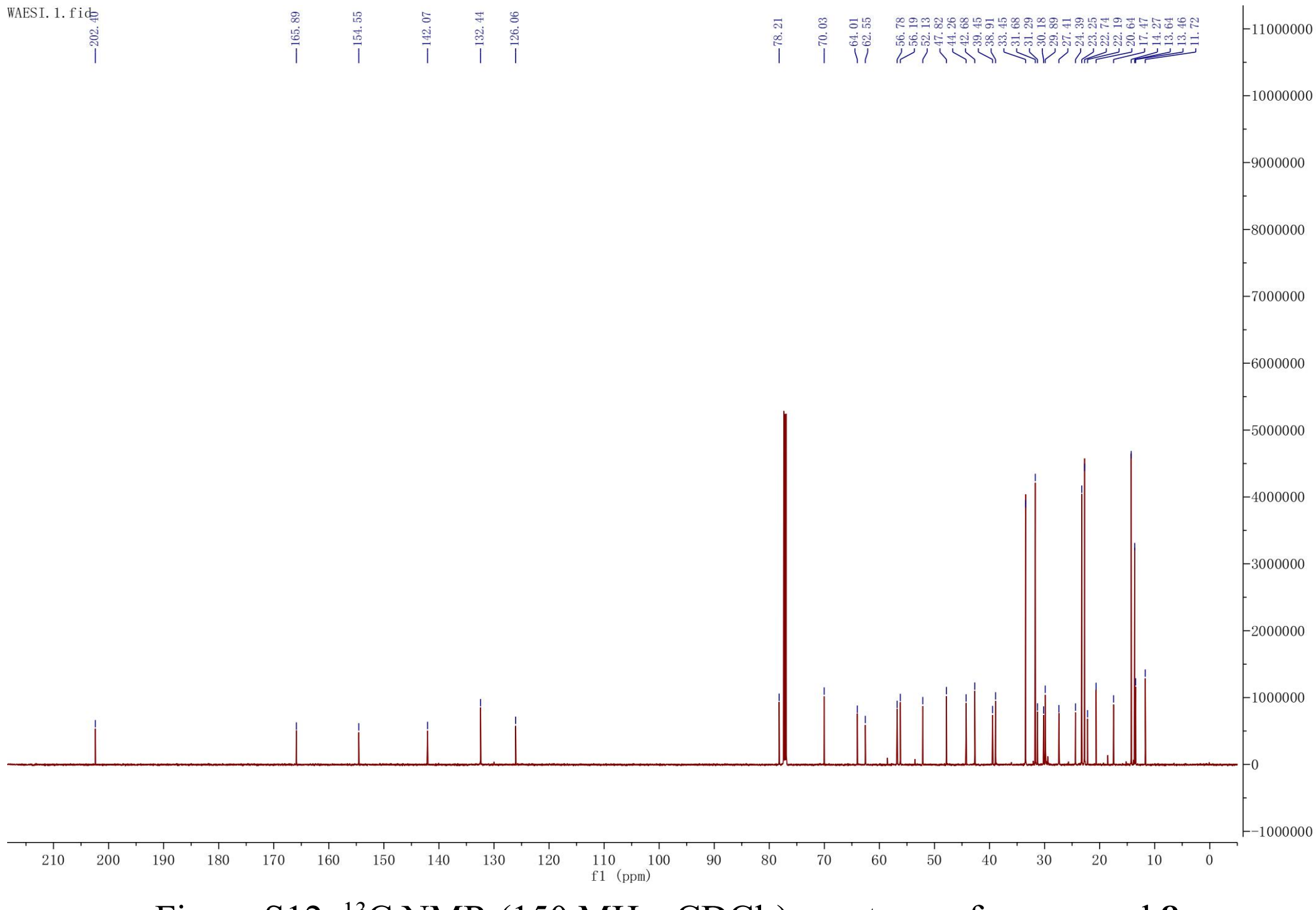

Figure S12. ^{13}C NMR (150 MHz, $CDCl_3$) spectrum of compound **8**

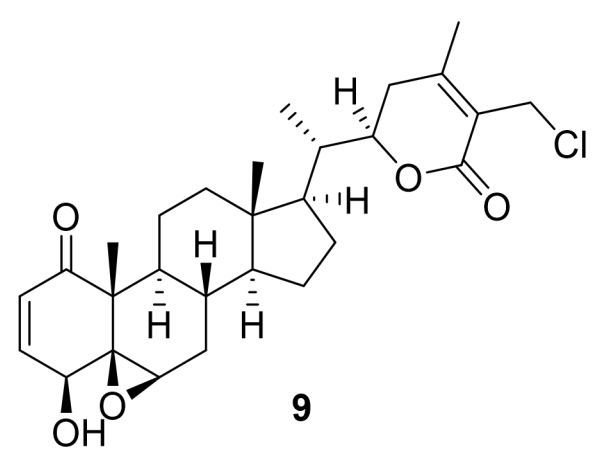

9

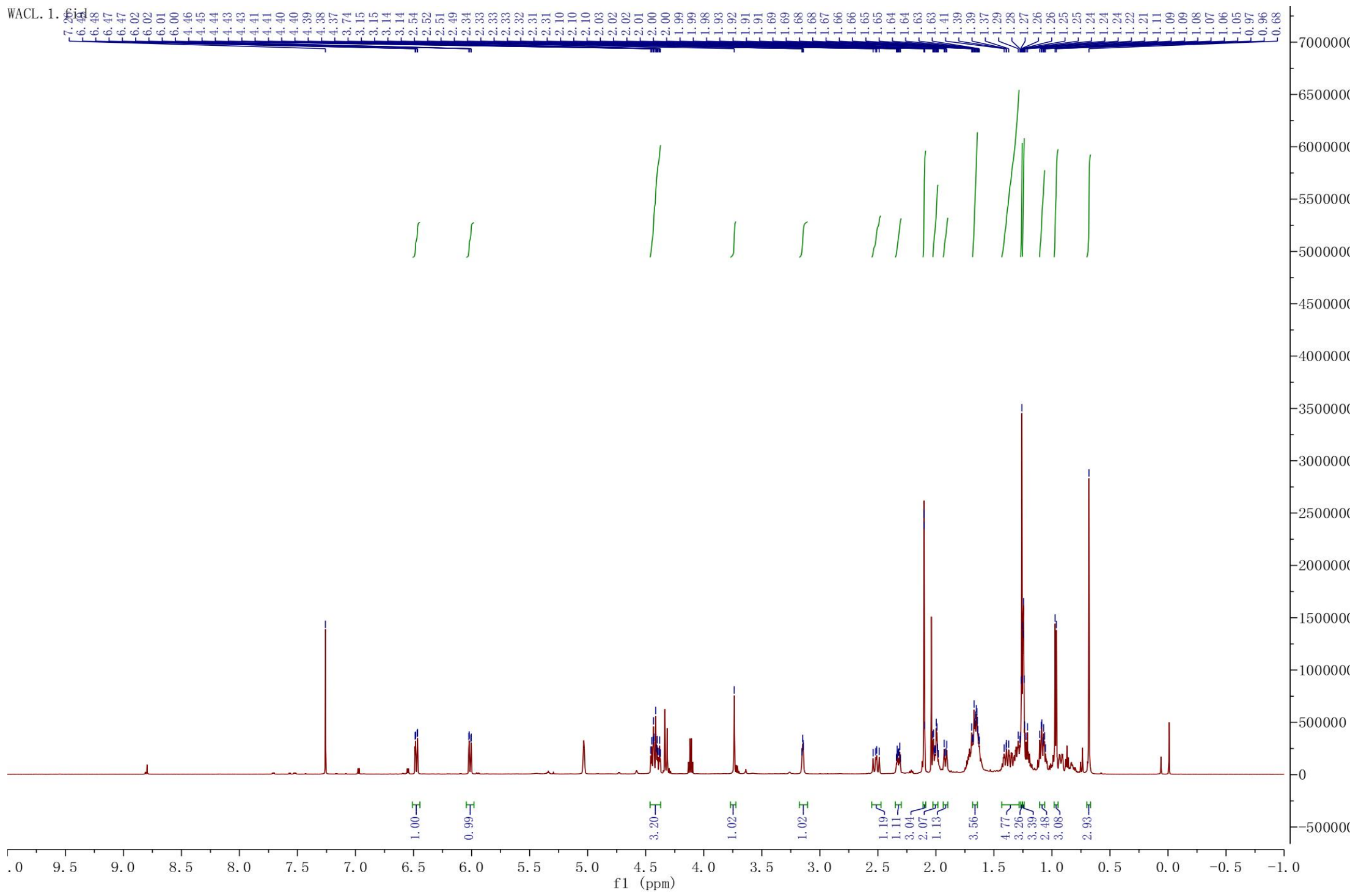

Figure S13. ^{1}H NMR (600 MHz, $CDCl_3$) spectrum of compound **9**

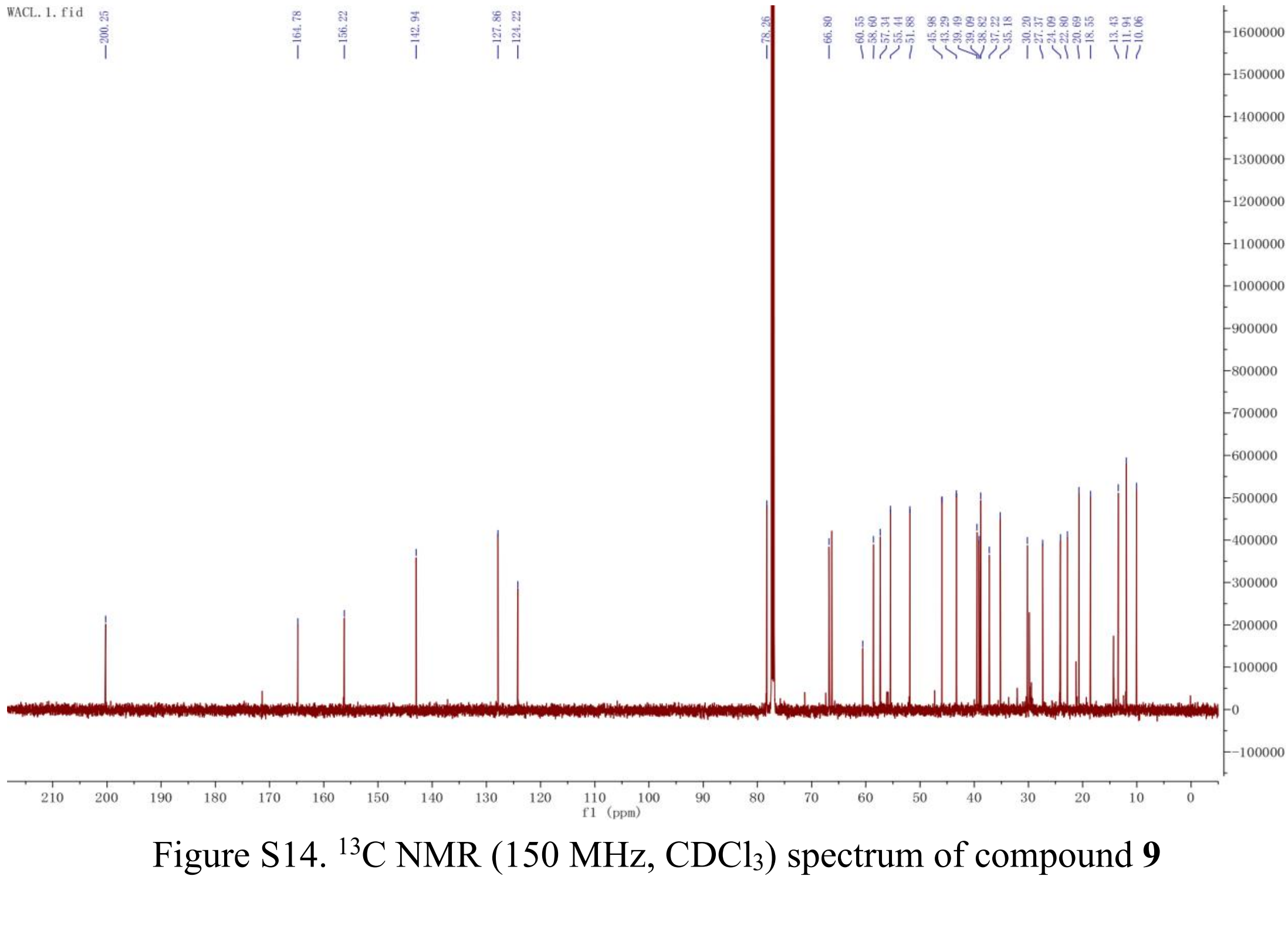

Figure S14. ^{13}C NMR (150 MHz, $CDCl_3$) spectrum of compound **9**

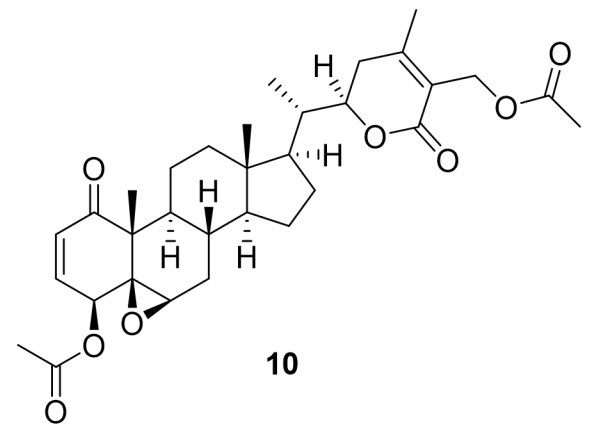

10

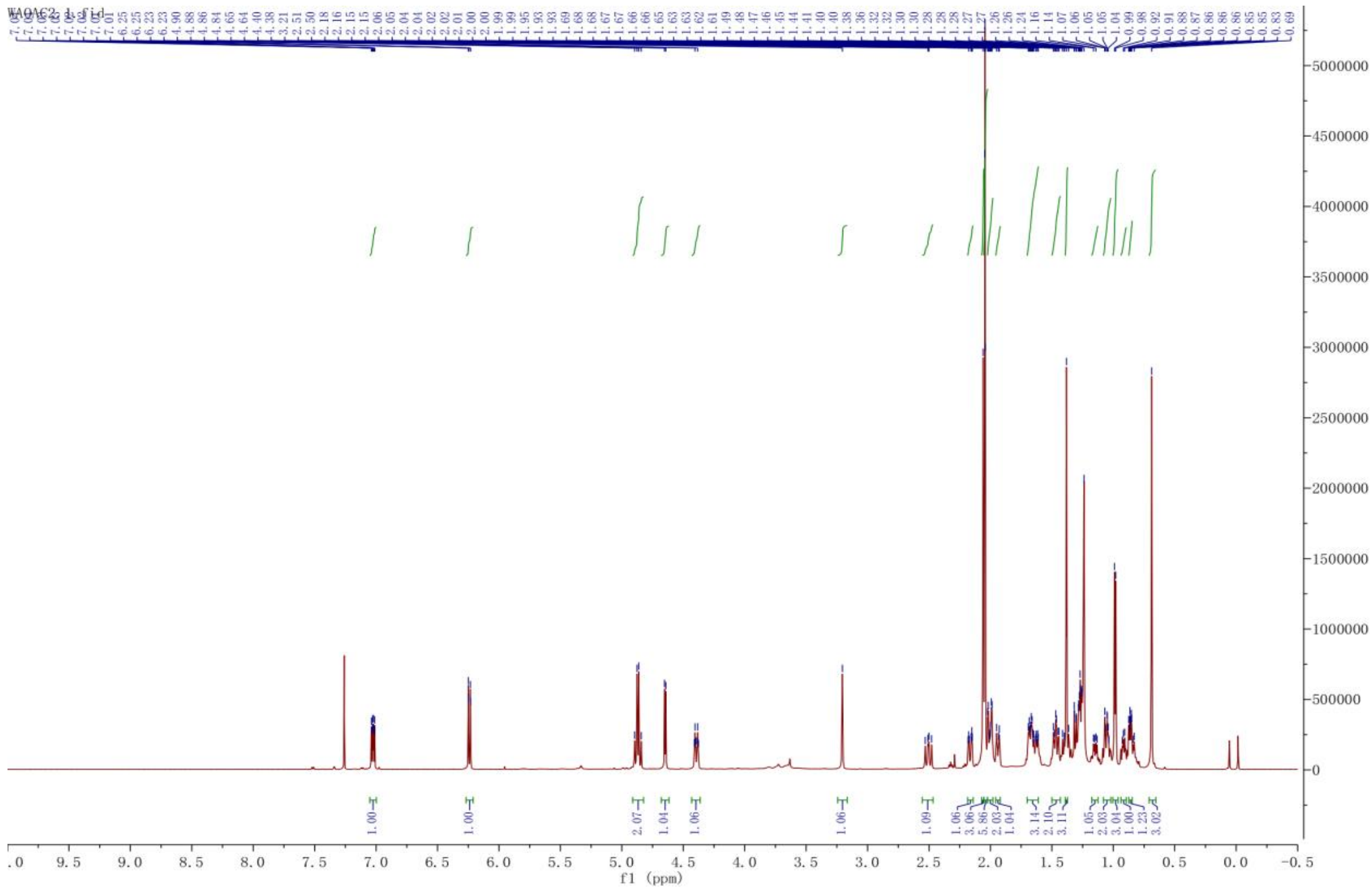

Figure S15. ^{1}H NMR (600 MHz, $CDCl_3$) spectrum of compound **10**

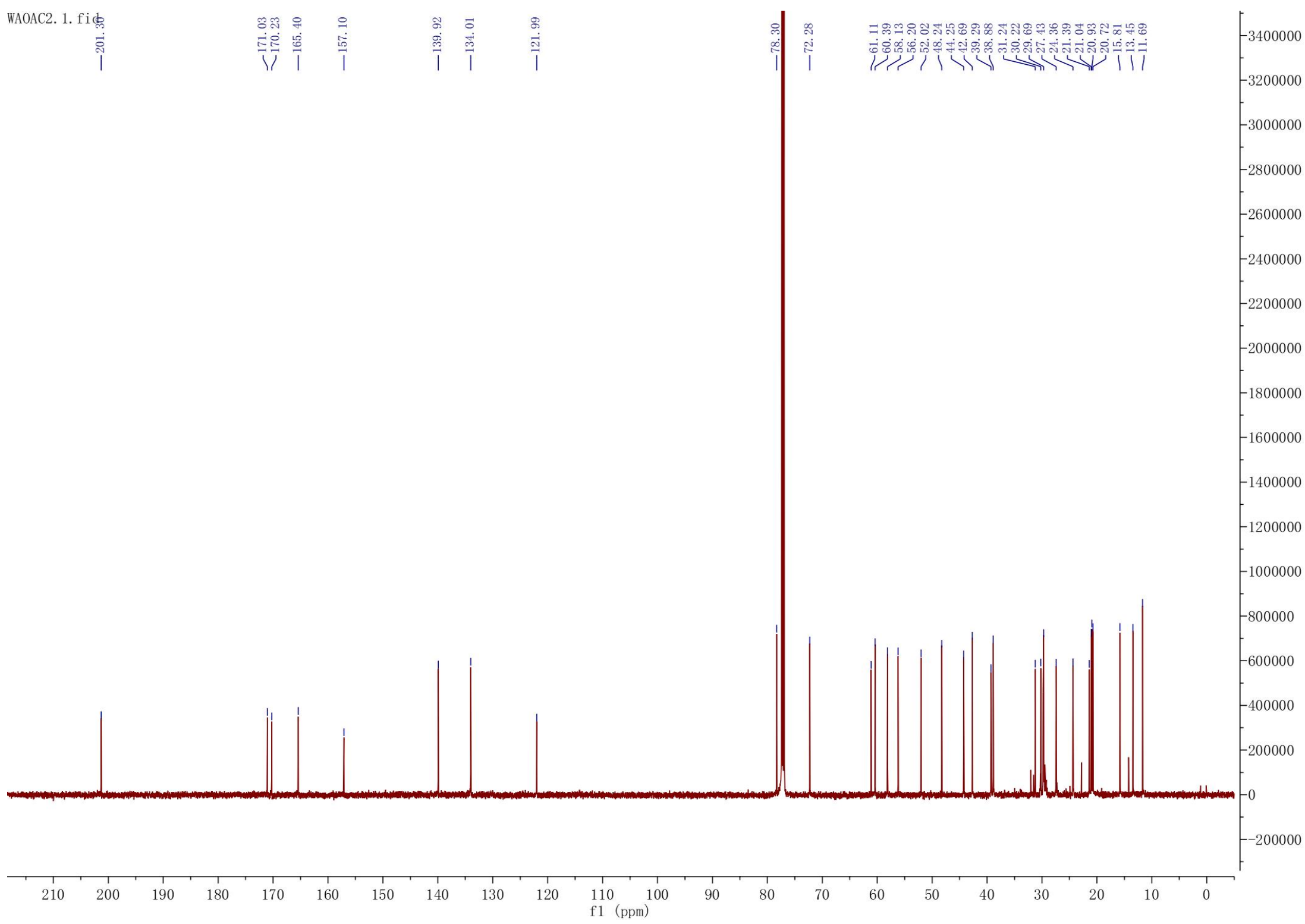

Figure S16. ^{13}C NMR (150 MHz, $CDCl_3$) spectrum of compound **10**

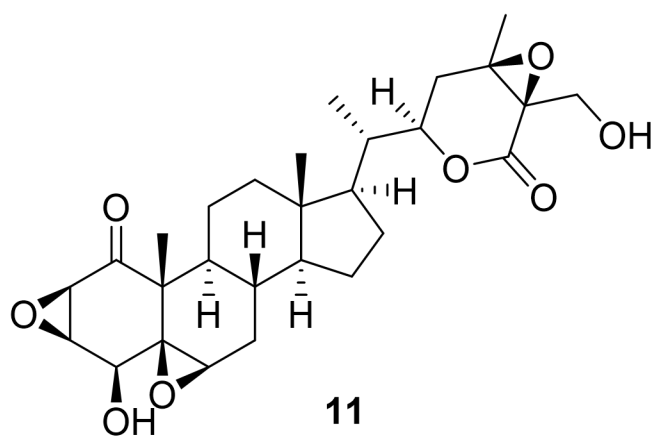

11

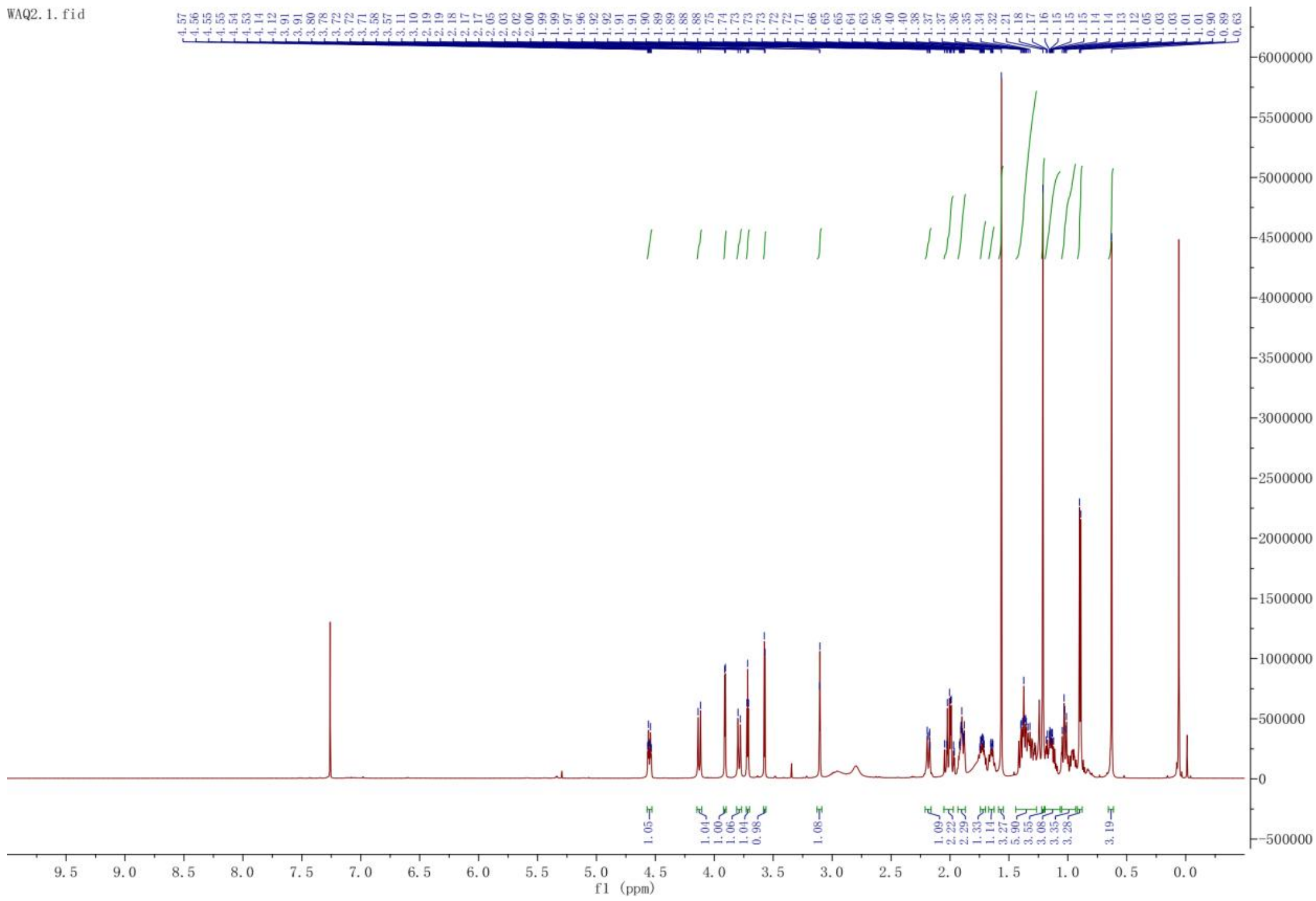

Figure S17. ^{1}H NMR (600 MHz, $CDCl_3$) spectrum of compound **11**

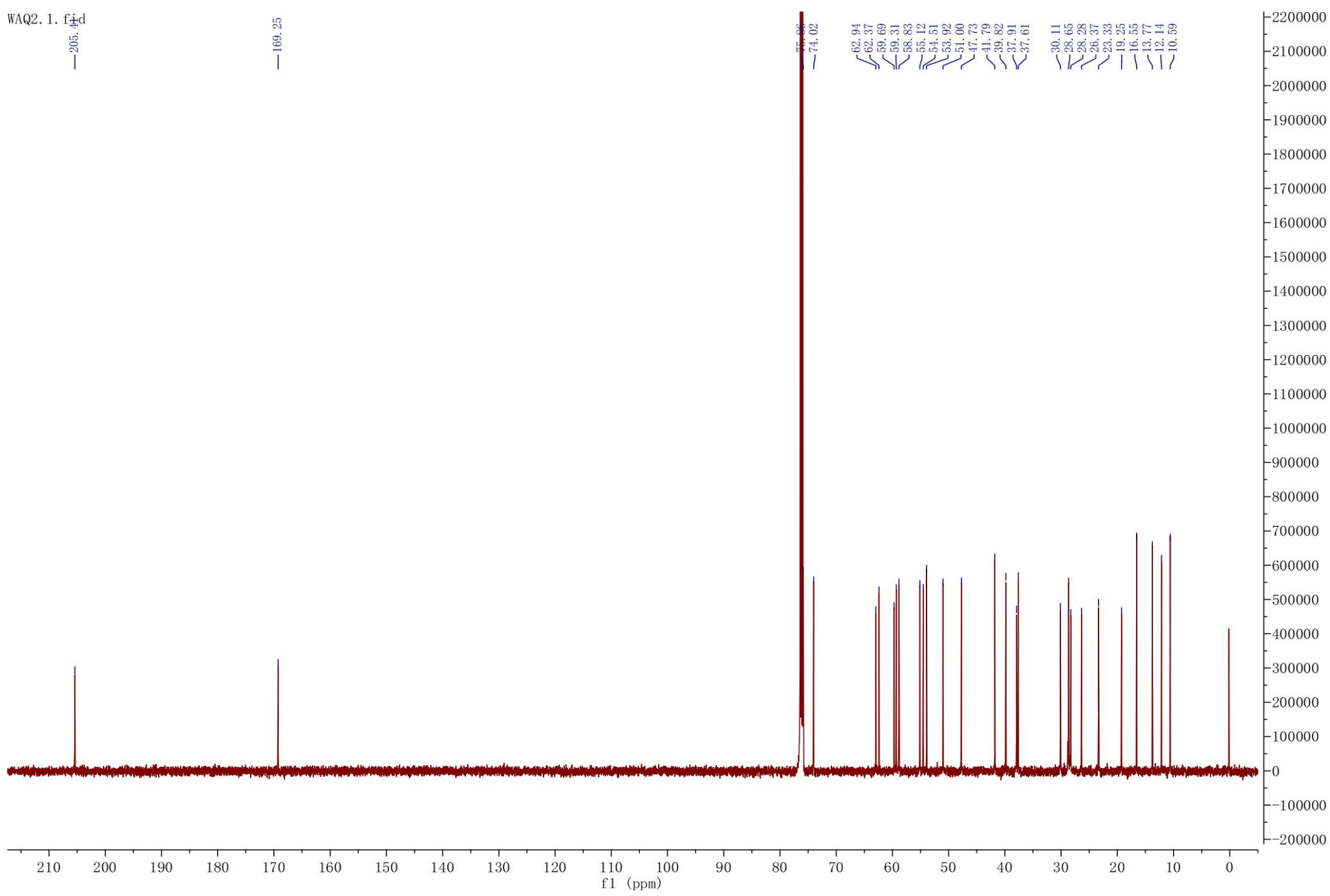

Figure S18. ^{13}C NMR (150 MHz, $CDCl_3$) spectrum of compound **11**

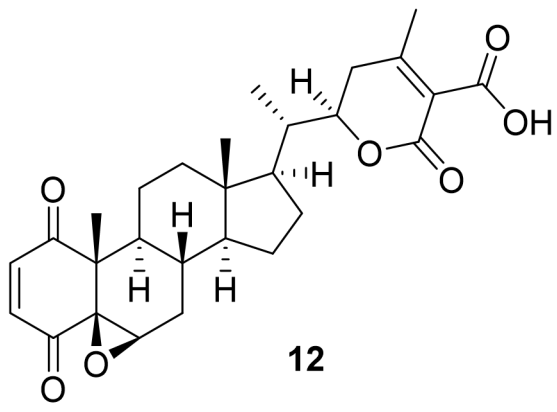

12

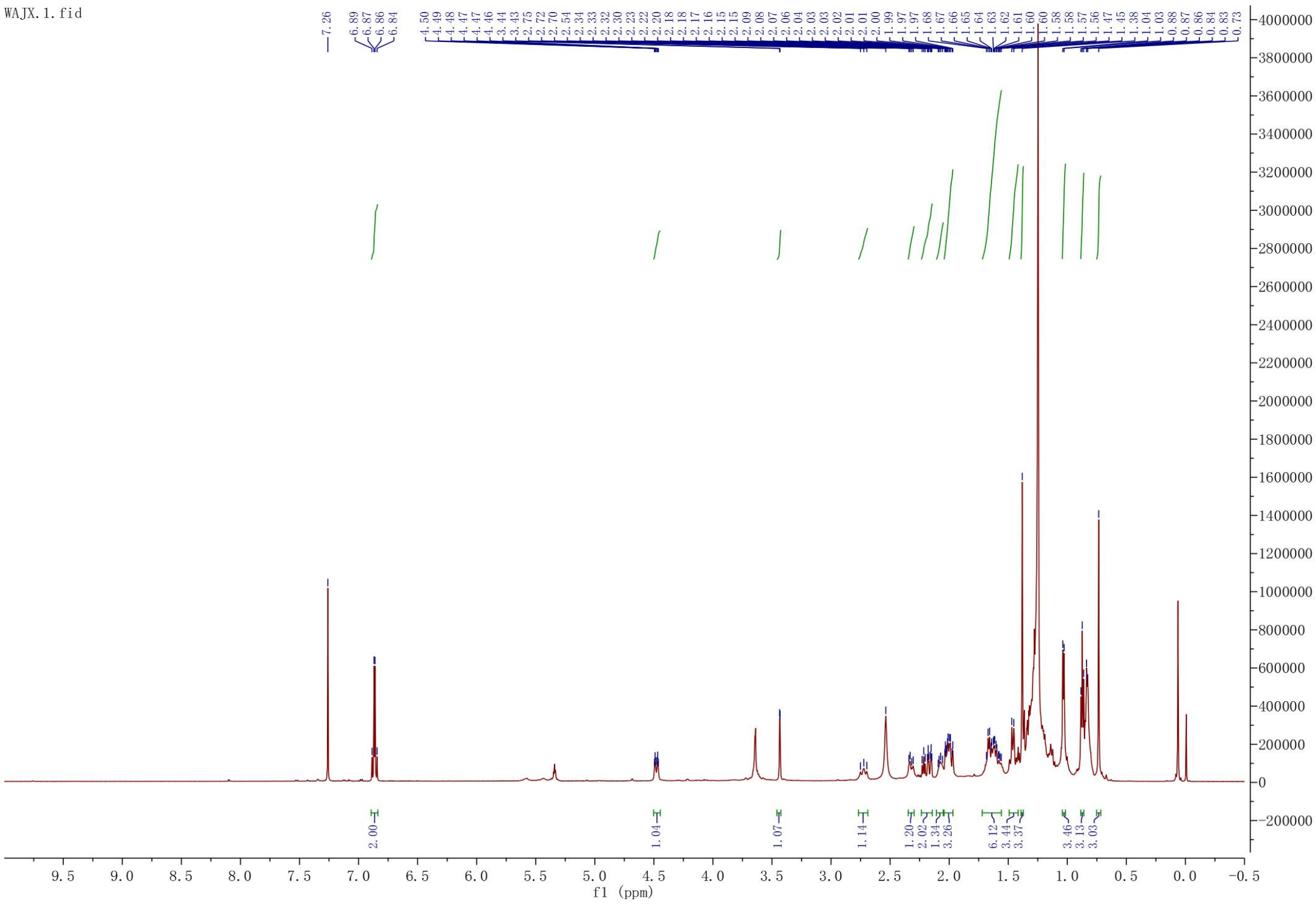

Figure S19. ^{1}H NMR (600 MHz, $CDCl_3$) spectrum of compound **12**

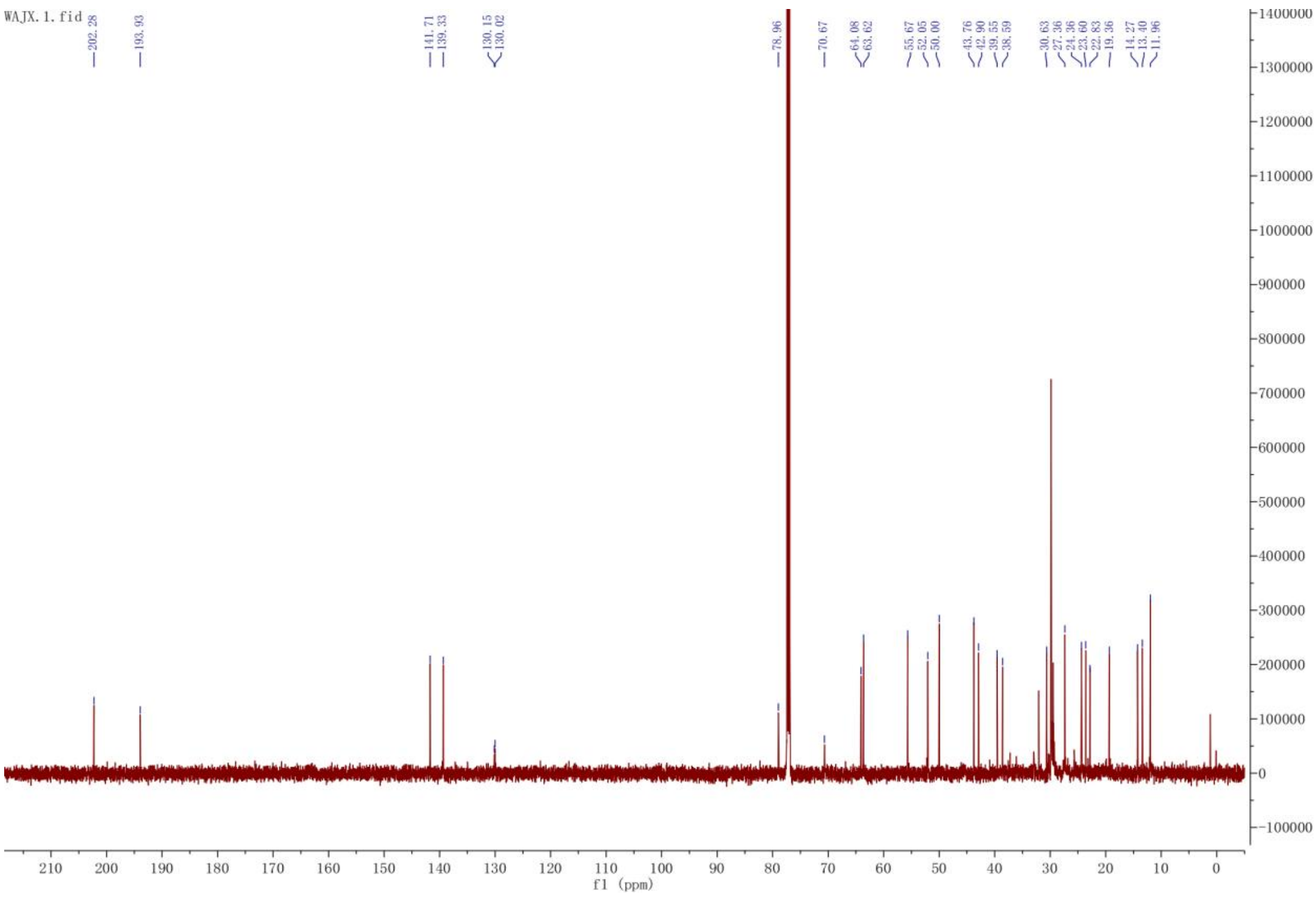

Figure S20. ^{13}C NMR (150 MHz, $CDCl_3$) spectrum of compound **12**

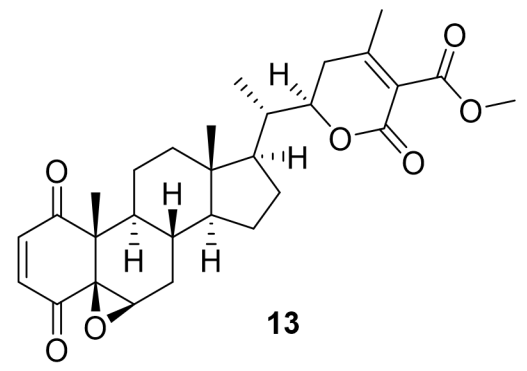

13

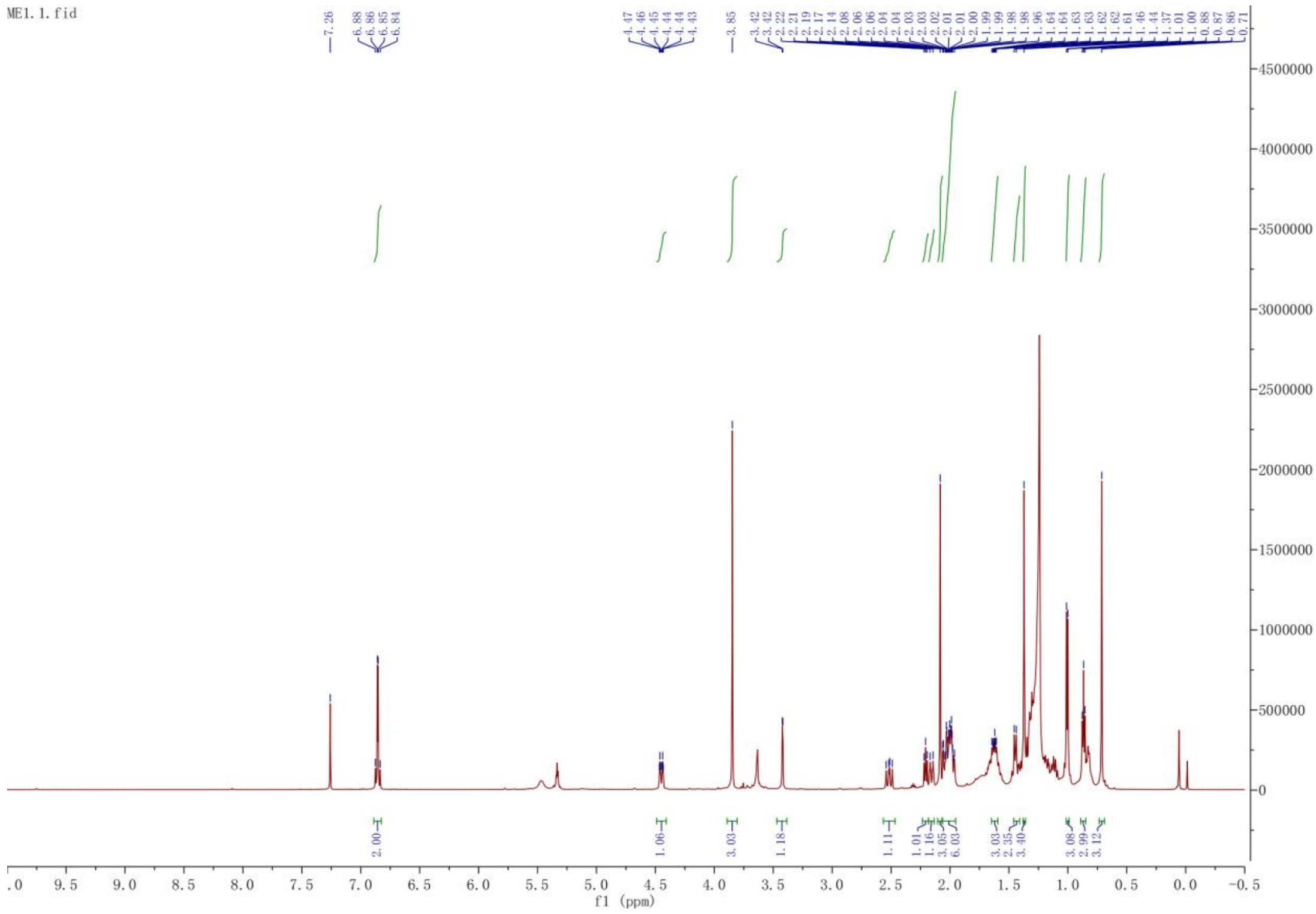

Figure S21. ^{1}H NMR (600 MHz, $CDCl_3$) spectrum of compound **13**

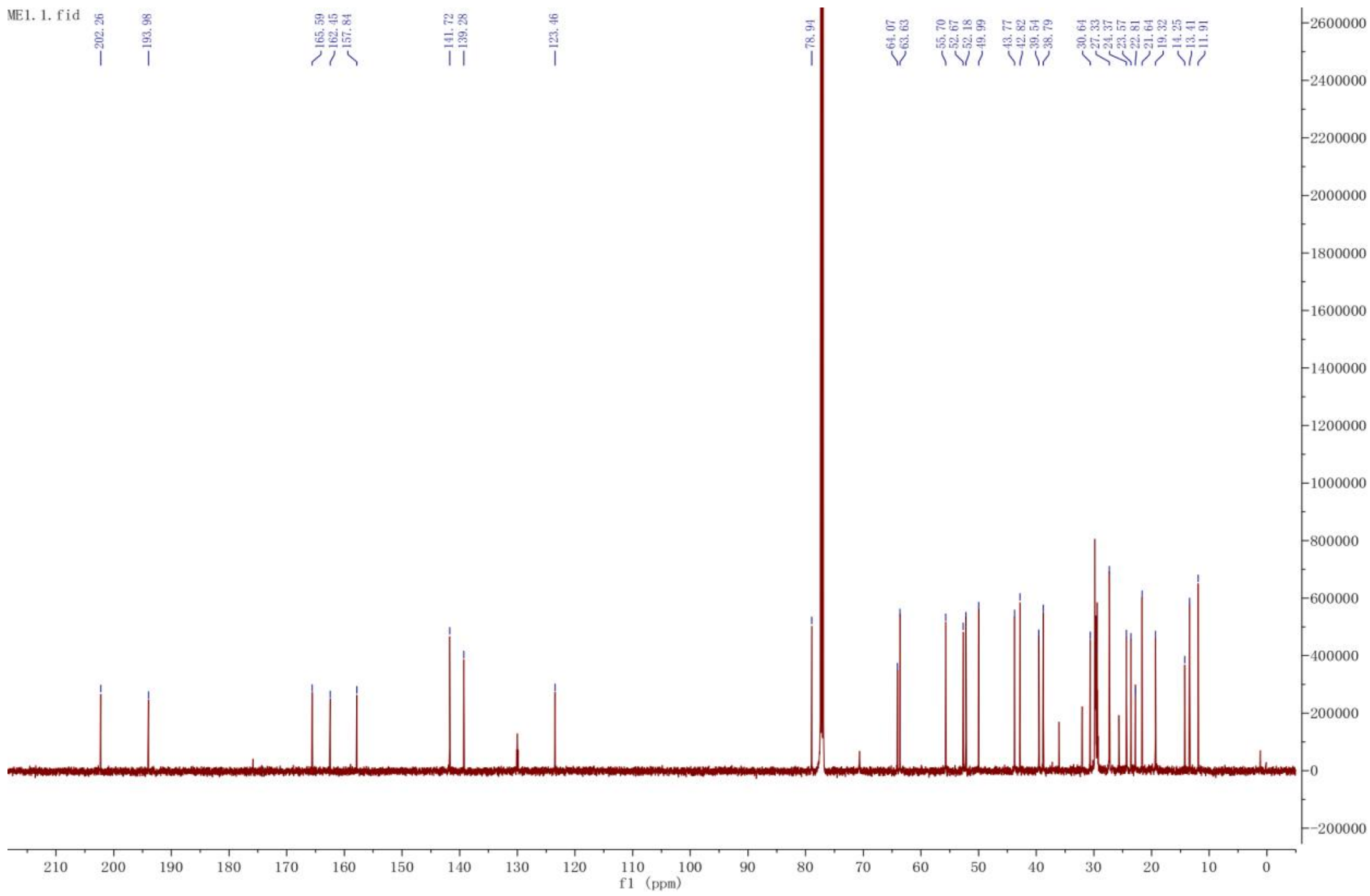

Figure S22. ^{13}C NMR (150 MHz, $CDCl_3$) spectrum of compound **13**

III: HRMS Spectra

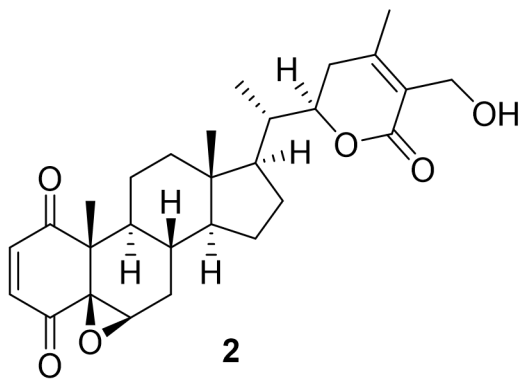

2

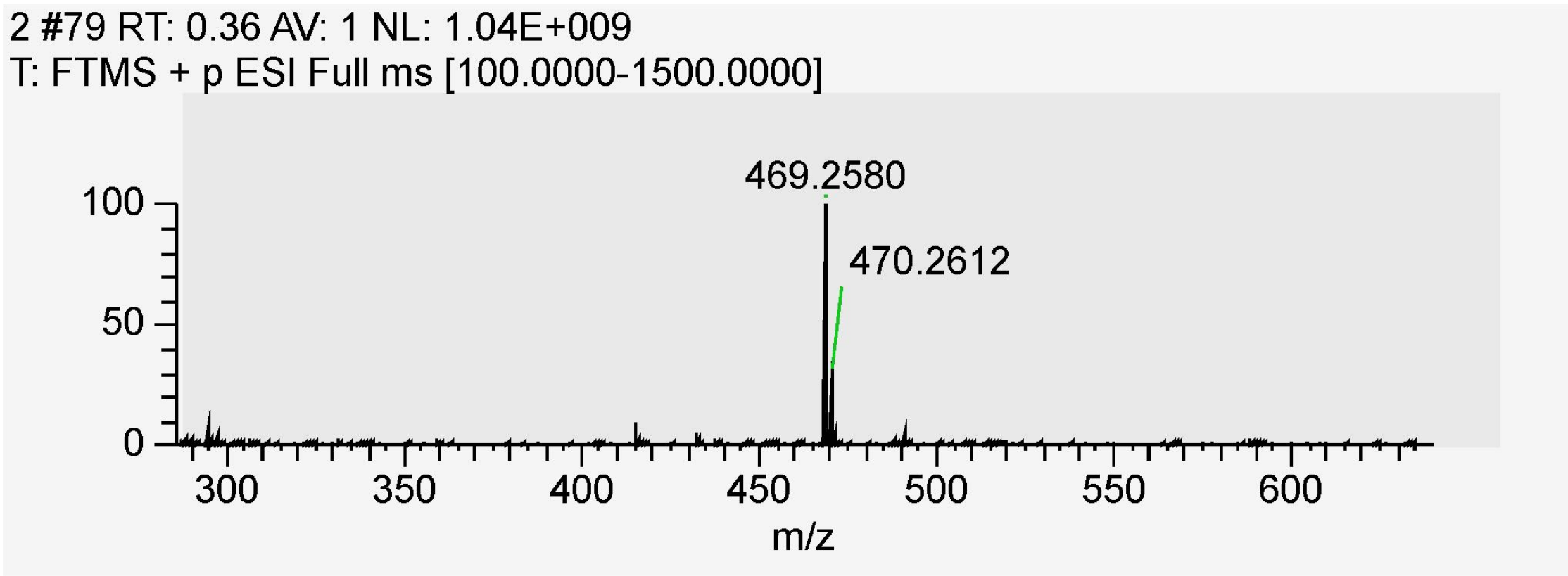

Figure S23. HRMS spectrum of compound **2**

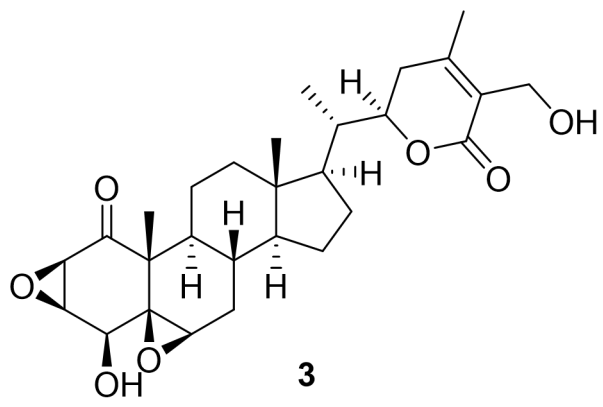

3

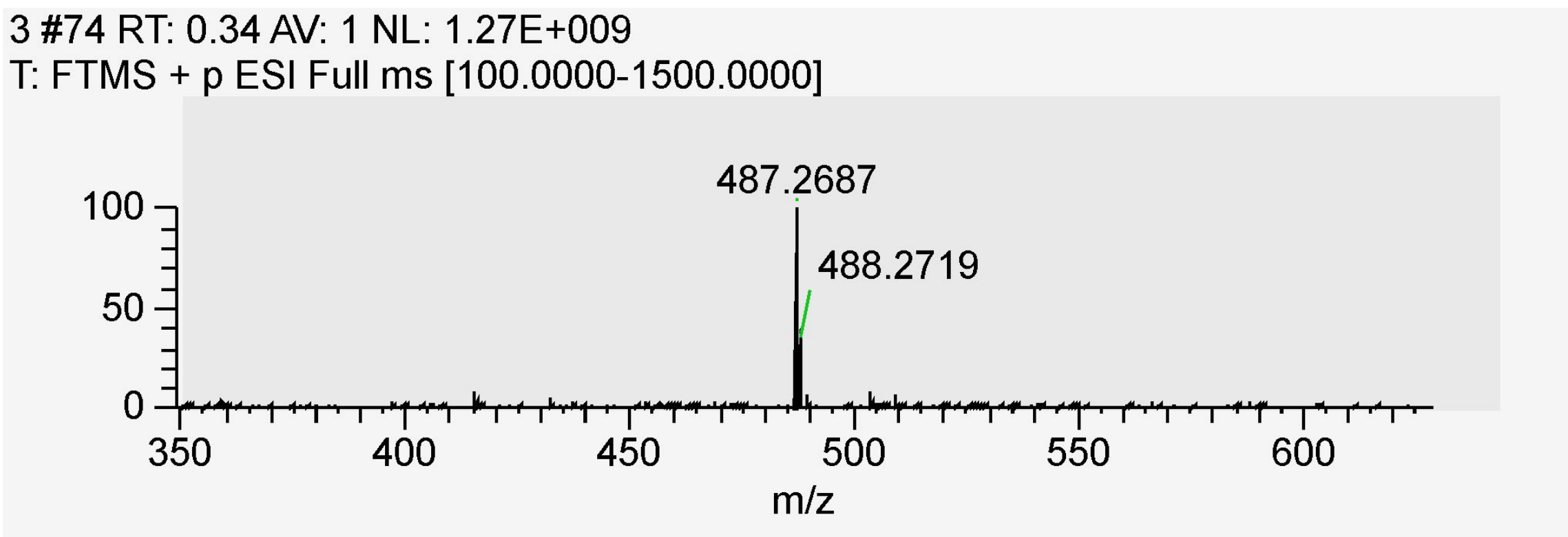

Figure S24. HRMS spectrum of compound **3**

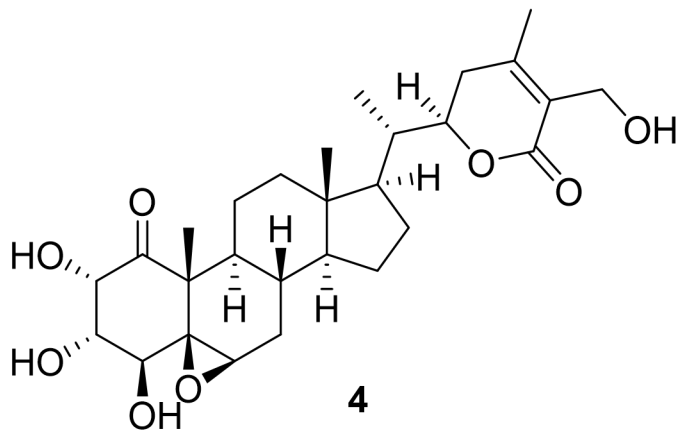

4

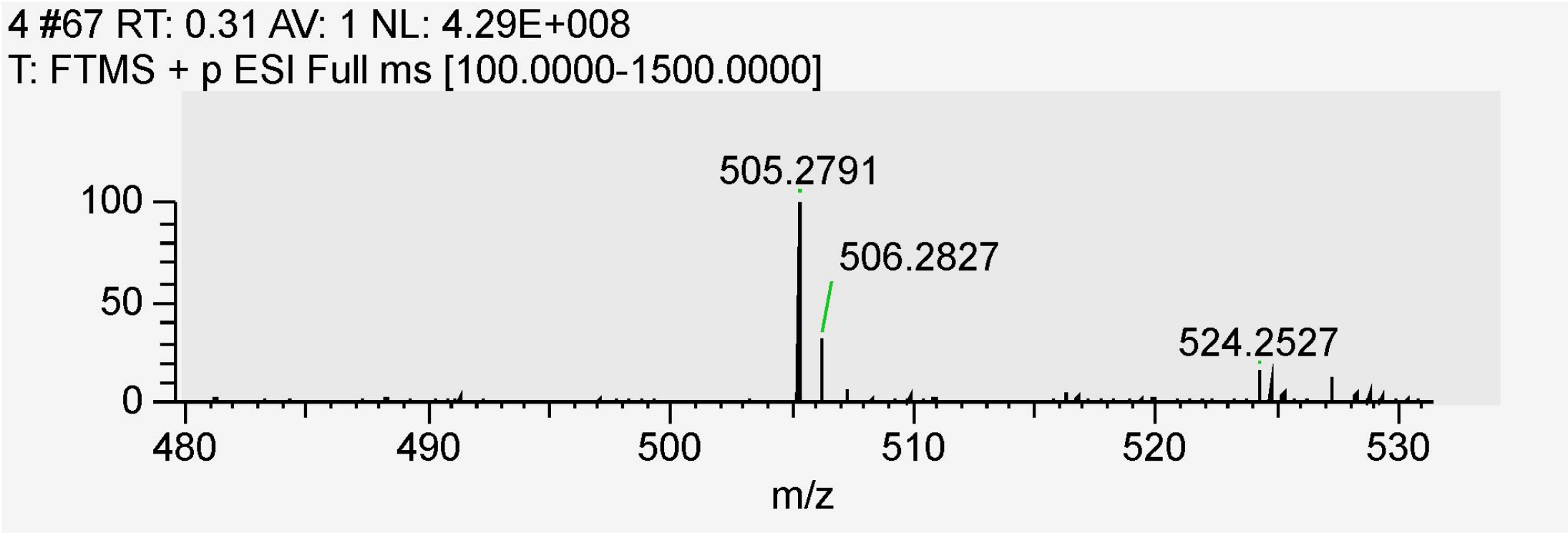

Figure S25. HRMS spectrum of compound **4**

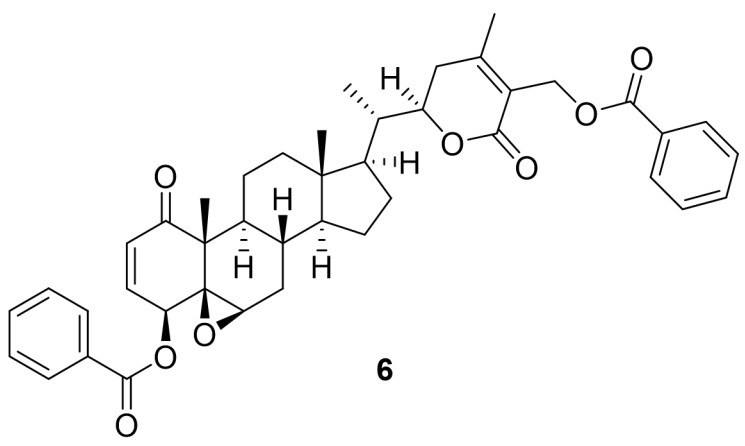

6

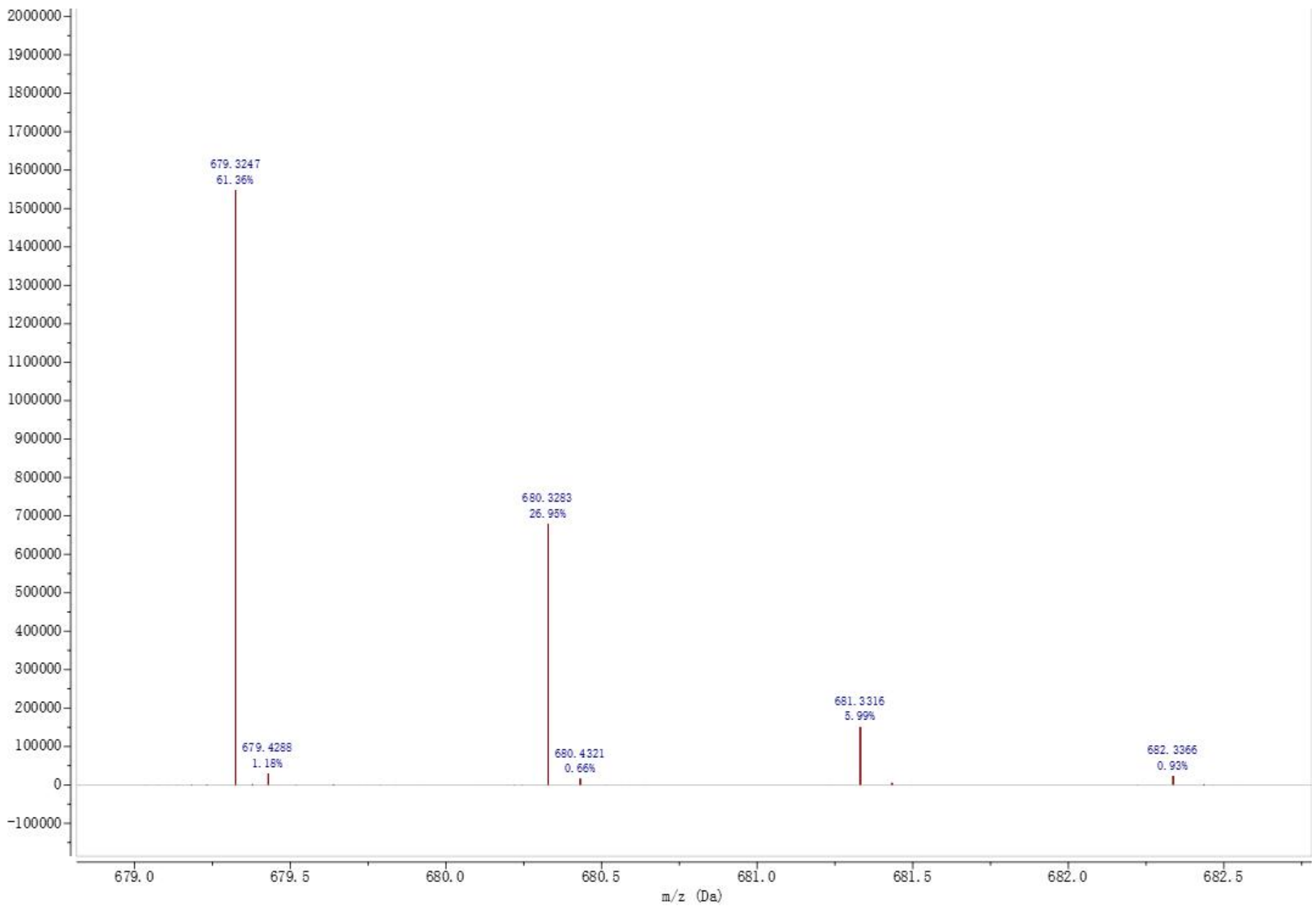

Figure S26. HRMS spectrum of compound **6**

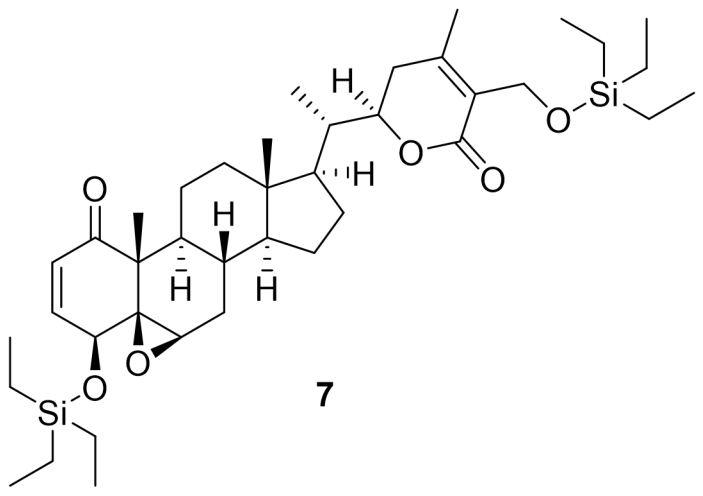

7

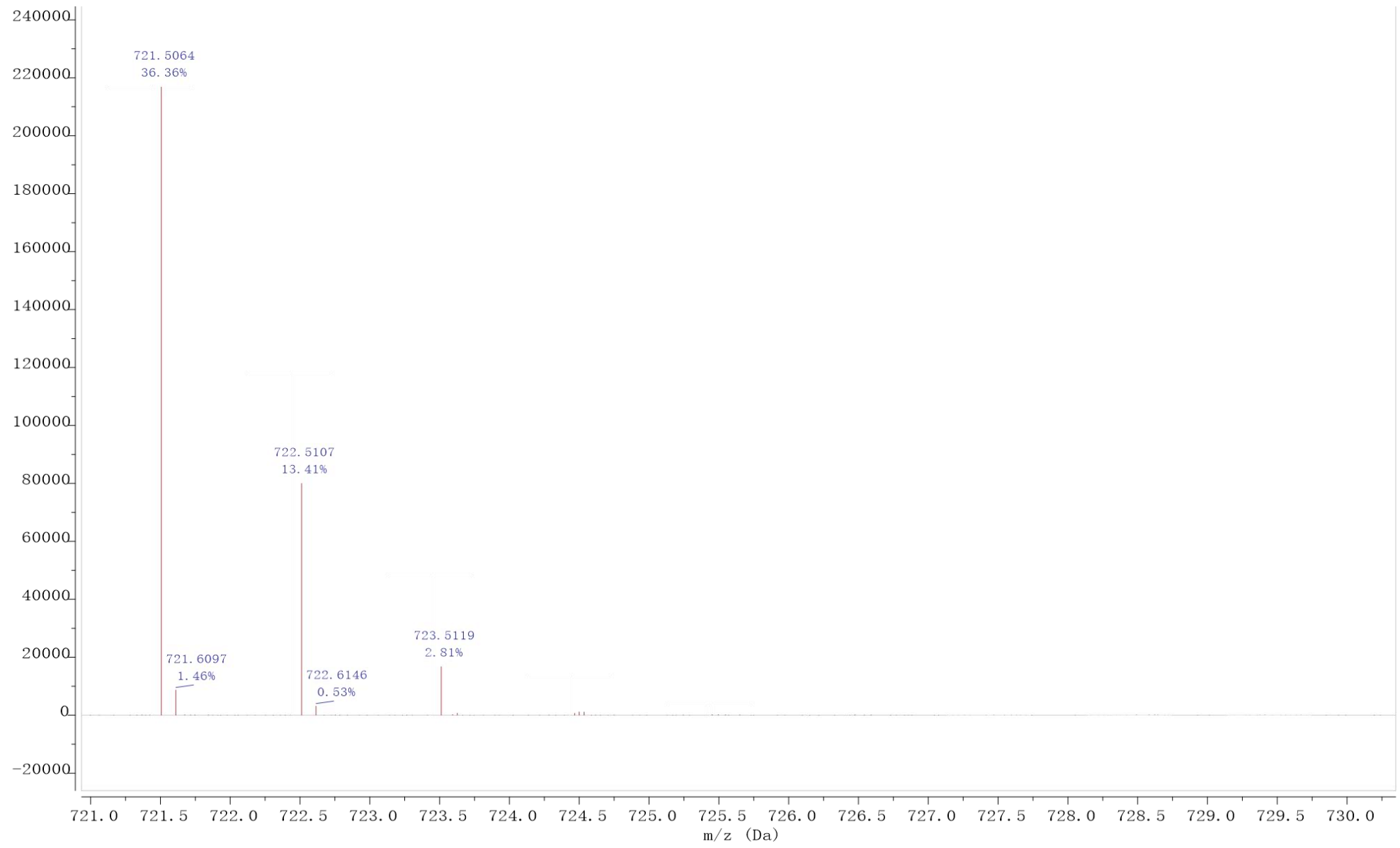

Figure S27. HRMS spectrum of compound **7**

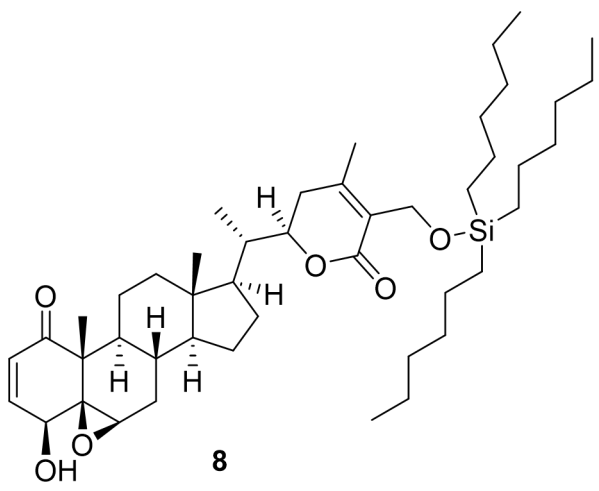

8

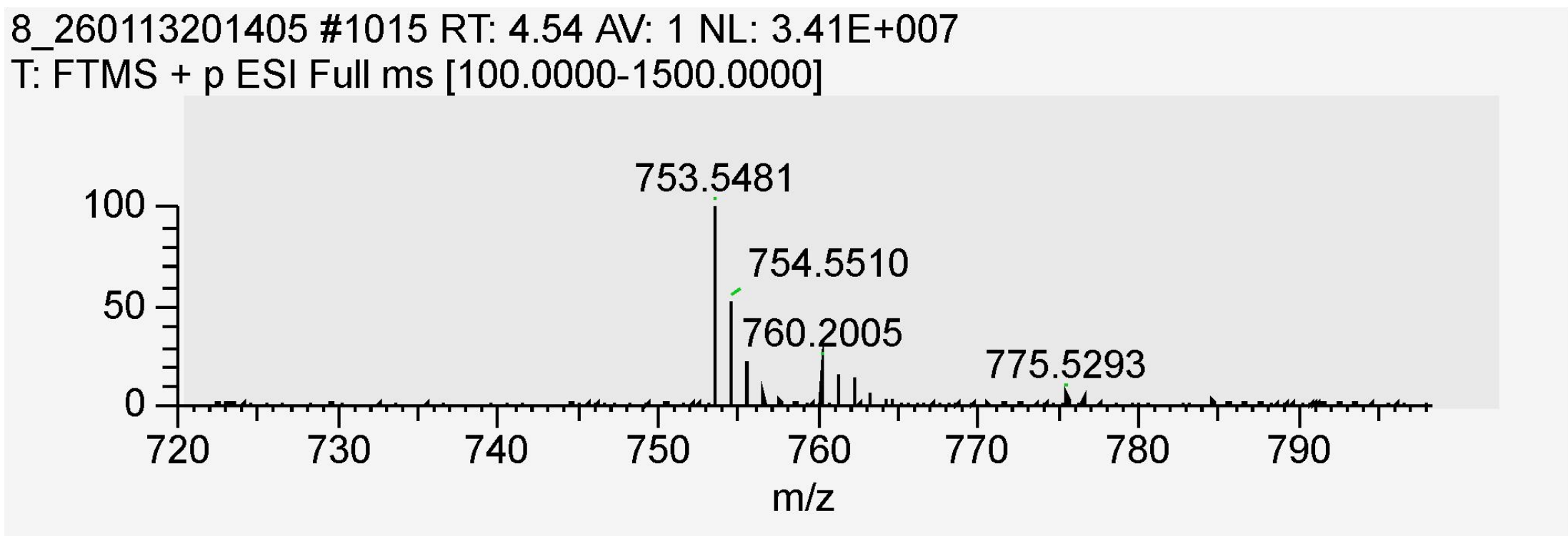

Figure S28. HRMS spectrum of compound **8**

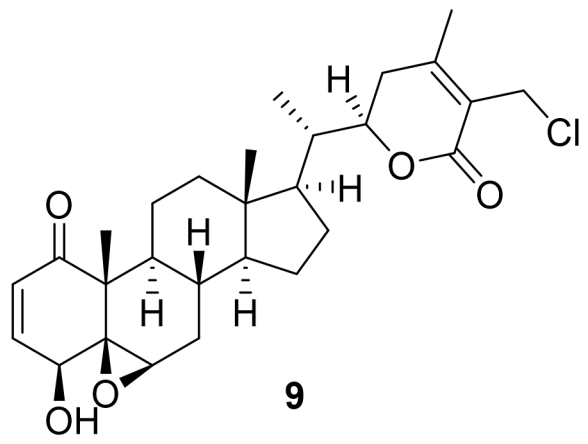

9

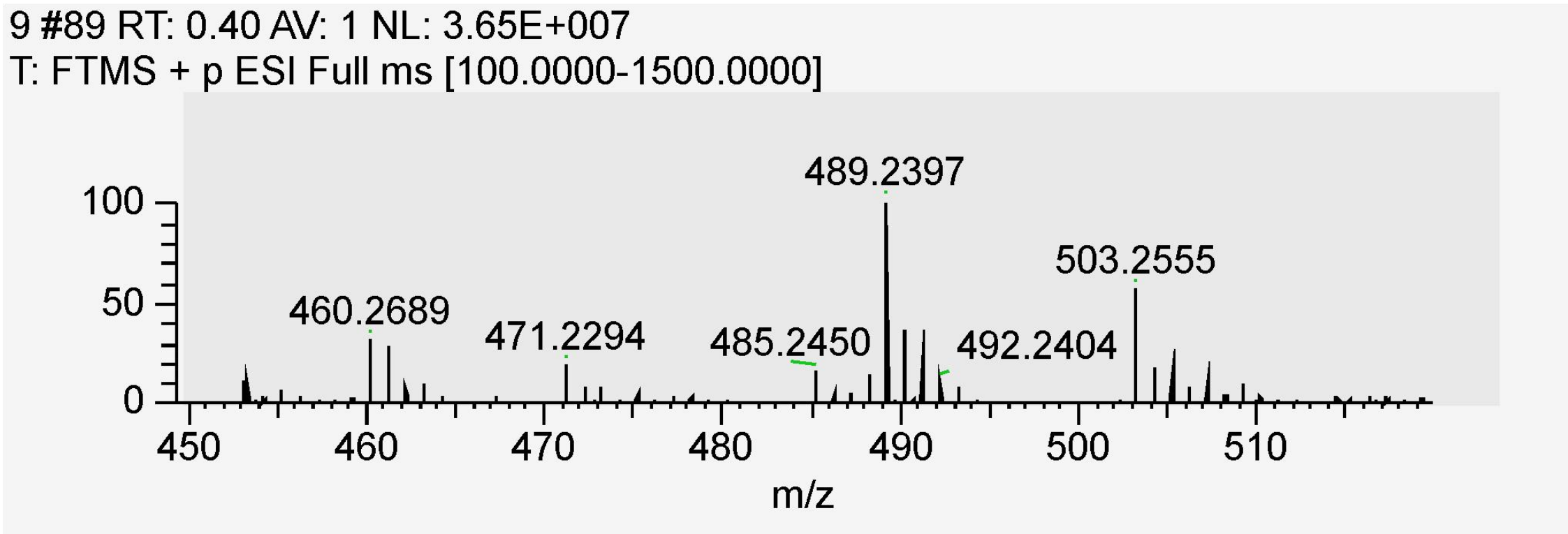

Figure S29. HRMS spectrum of compound **9**

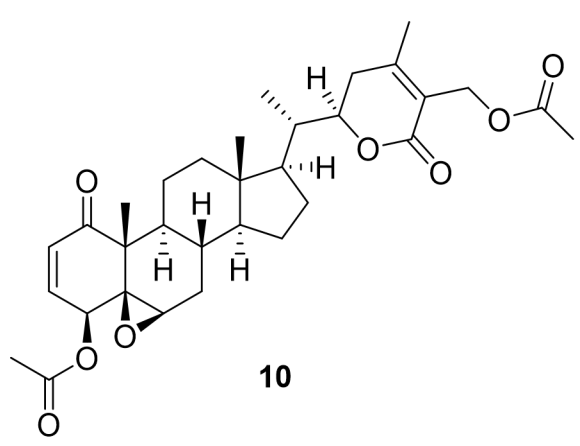

10

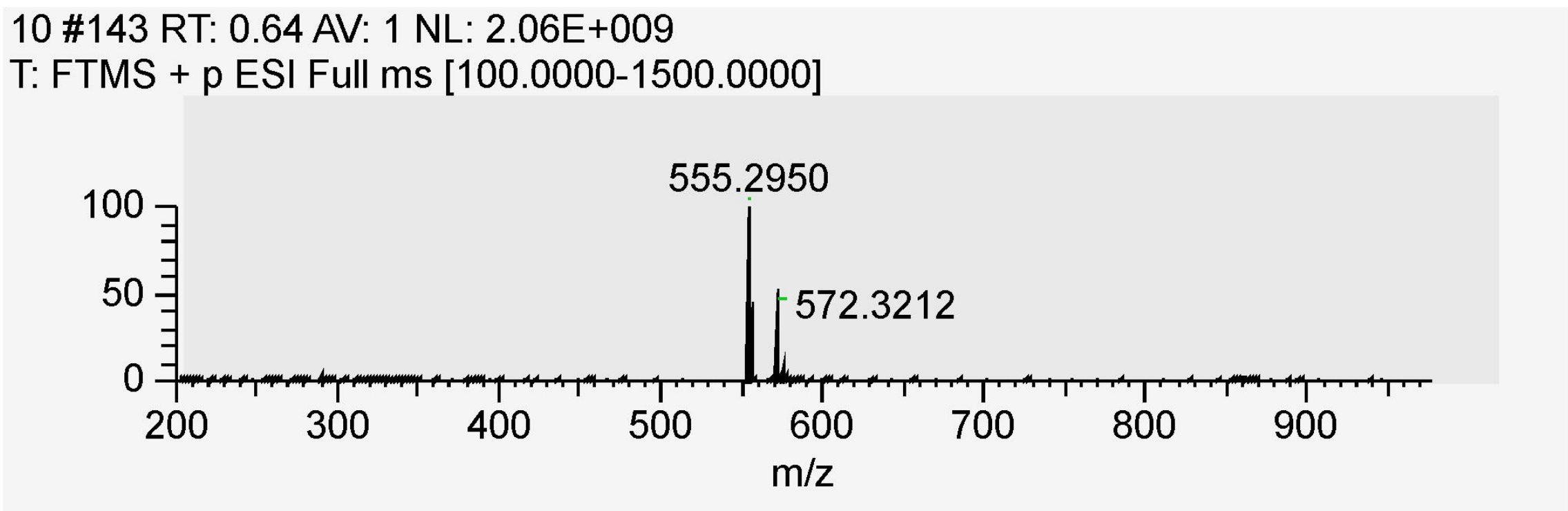

Figure S30. HRMS spectrum of compound **10**

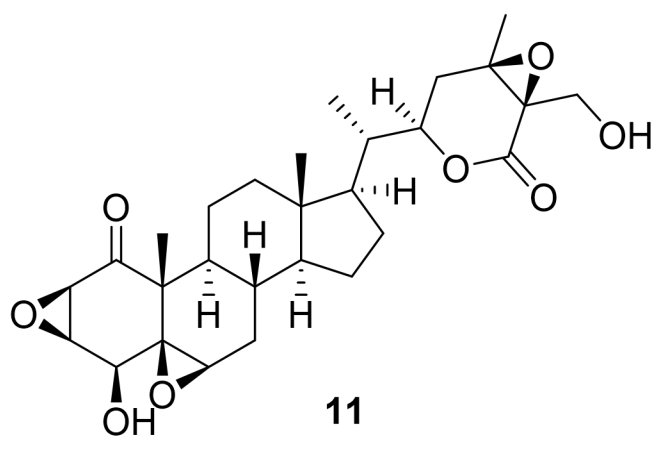

11

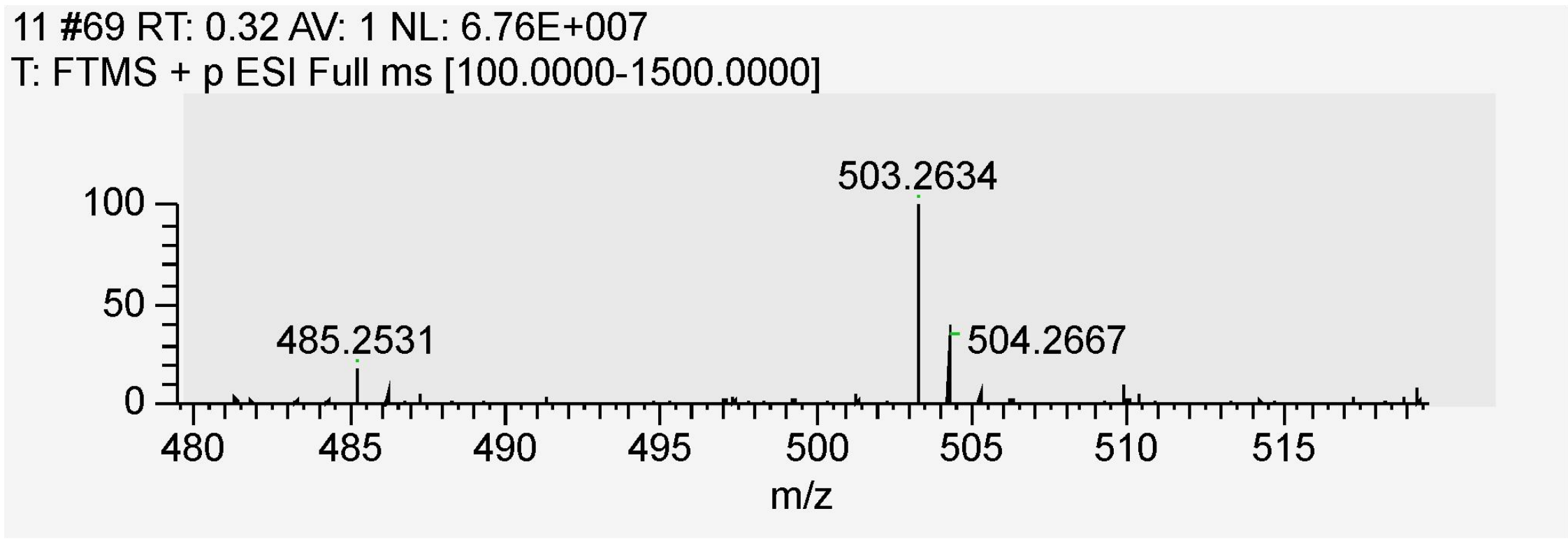

Figure S31. HRMS spectrum of compound **11**

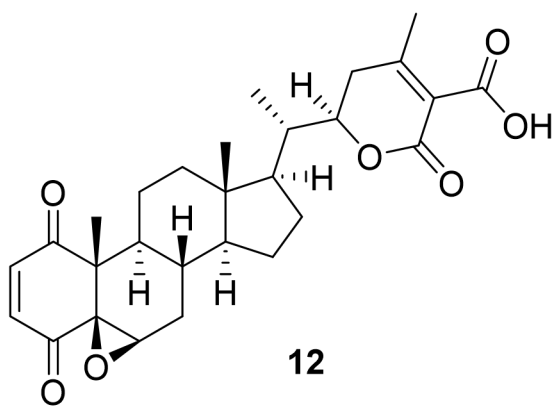

12

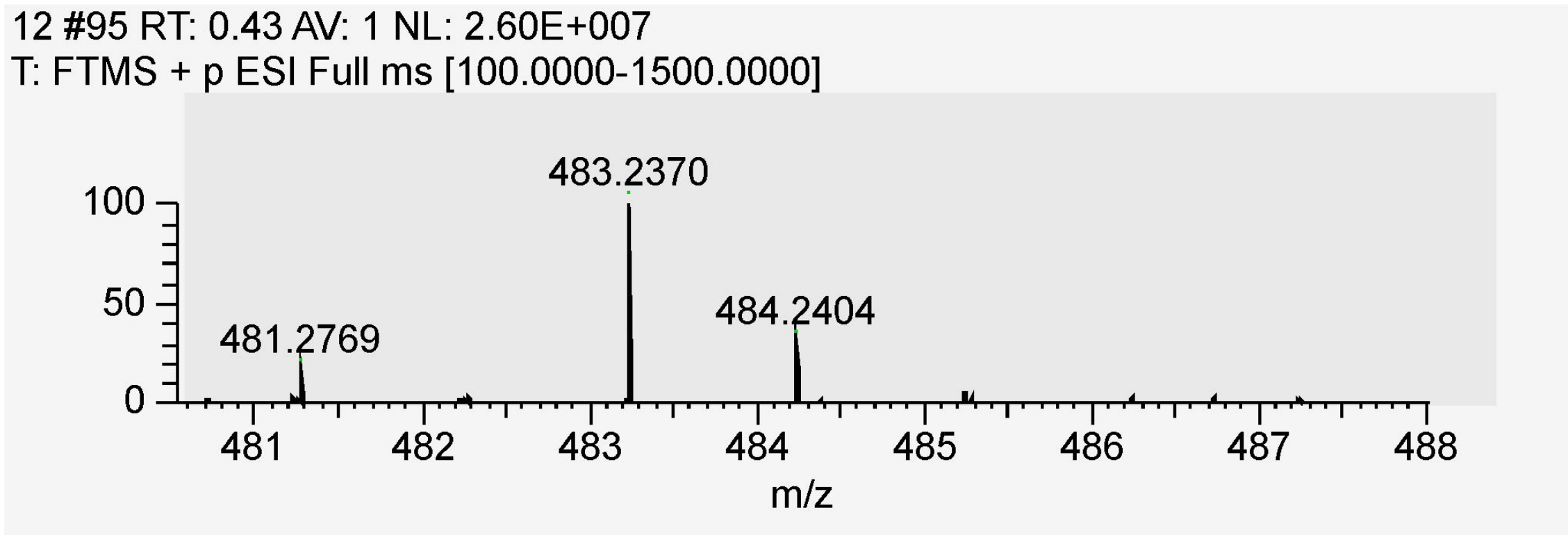

Figure S32. HRMS spectrum of compound **12**

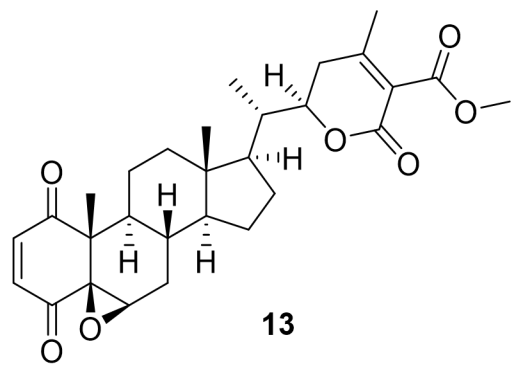

13

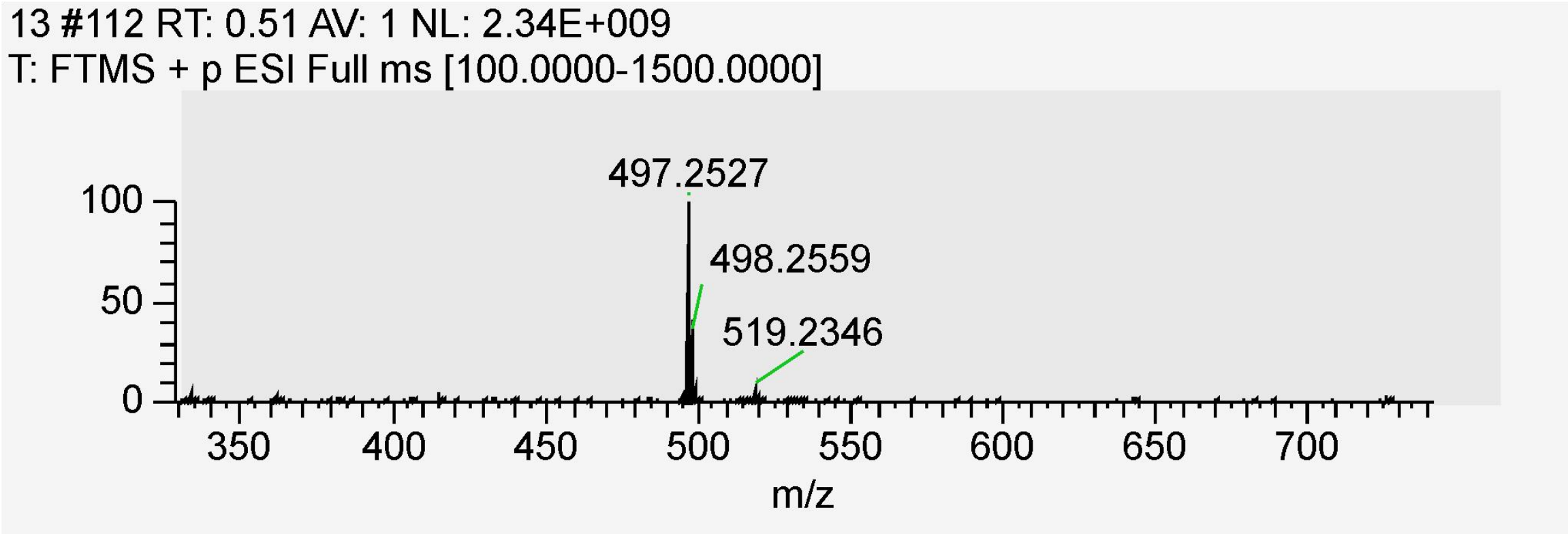

Figure S33. HRMS spectrum of compound **13**
